# Supplementary material for: Systems-level analyses of protein-protein interaction network dysfunctions via epichaperomics identify cancer-specific mechanisms of stress adaptation
Source: Nat Commun. 2023 Jun 23;14:3742. doi: 10.1038/s41467-023-39241-7 (PMC10290137; doi:10.1038/s41467-023-39241-7)
Supplement: Supplementary file 1 — Supplementary Information [file 41467_2023_39241_MOESM1_ESM.pdf]

Supplementary Information

Systems-level analysis of protein-protein interaction network alterations through epichaperomics identify cancer-specific mechanisms of stress adaptation

Rodina et al.

Contains:

Supplementary Figures 1 through 30

Supplementary Notes 1, 2

Supplementary References

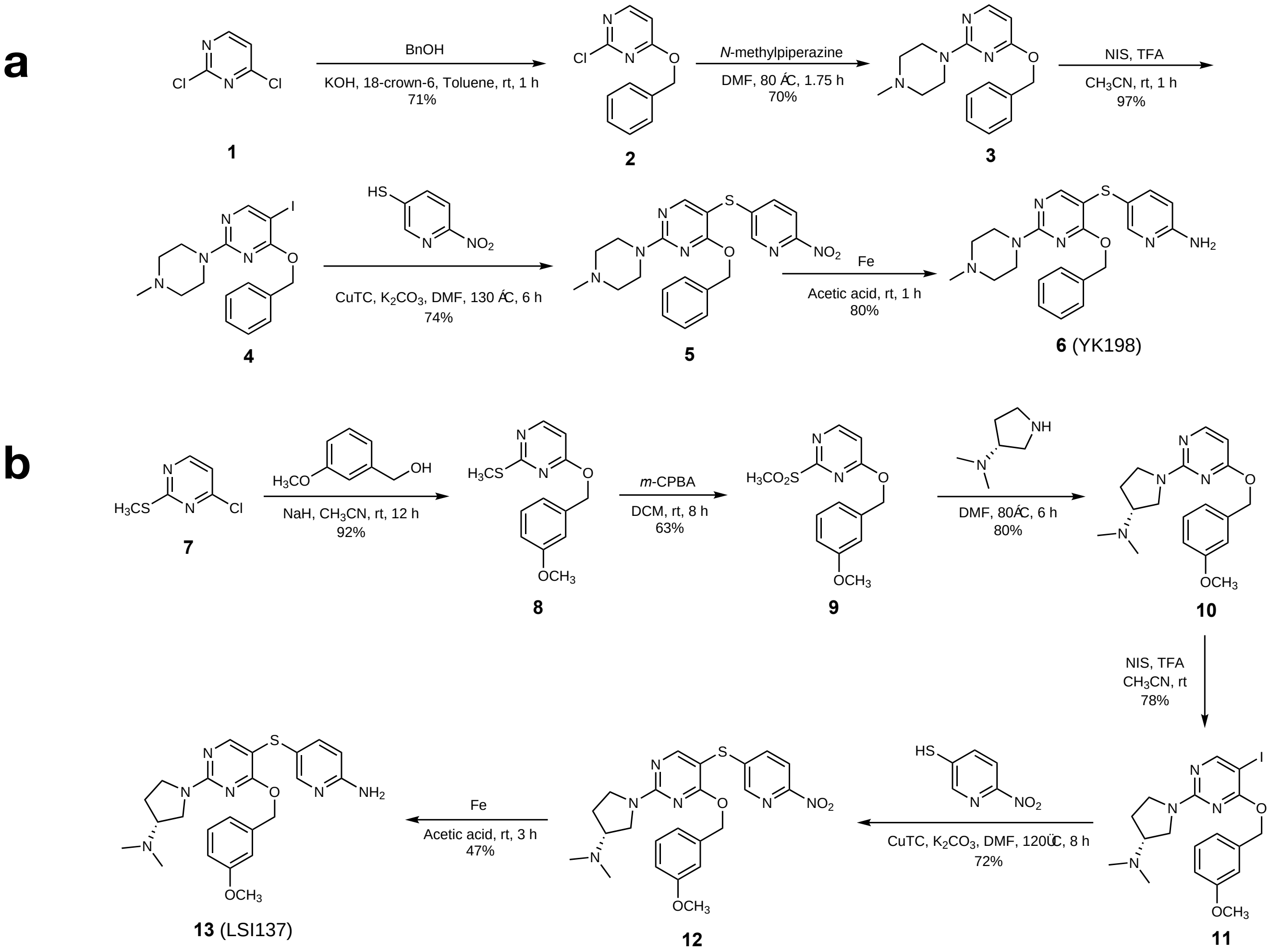

Supplementary Figure 1. Synthetic scheme for the preparation of YK198 and LSI137 probes. a Synthesis of YK198 (**6**) commenced with the reaction of compound **1** with benzyl alcohol (BnOH), followed by treating the resulting compound **2** with N-methylpiperazine to yield intermediate **3**. Compound **3** was then iodinated with NIS to yield **4**. The C-S coupling of **4** with the indicated phenyl thiol was achieved by CuTC and K_2CO_3 catalysis to obtain compound **5**. The nitro group of **5** was reduced in the presence of Fe and acetic acid to give YK198 in 80% yield. **b** Synthesis of LSI137 started from compound **7**. Sequential reaction of **7** with m-methoxybenzyl alcohol followed by S-oxidation with m-CPBA resulted in the intermediate **9**. Heating of **9** with (R)-N,N-dimethylpyrrolidin-3-amine in dimethylformamide (DMF) yielded compound **10**. This was then iodinated to yield **11**, which was then coupled with 6-nitropyridine-3-thiol in the presence of CuTC and K_2CO_3 to result in compound **12**. Reduction of **12** with iron and acetic acid led to LSI137 in 47% yield. NIS, N-iodosuccinimide; TFA, trifluoroacetic acid; CuTC, copper(I)thiophene-2-carboxylate; m-CPBA, meta-chloroperoxybenzoic acid; rt, room temperature. See Supplementary Note 1 for detailed description of probe synthesis, purification and characterization.

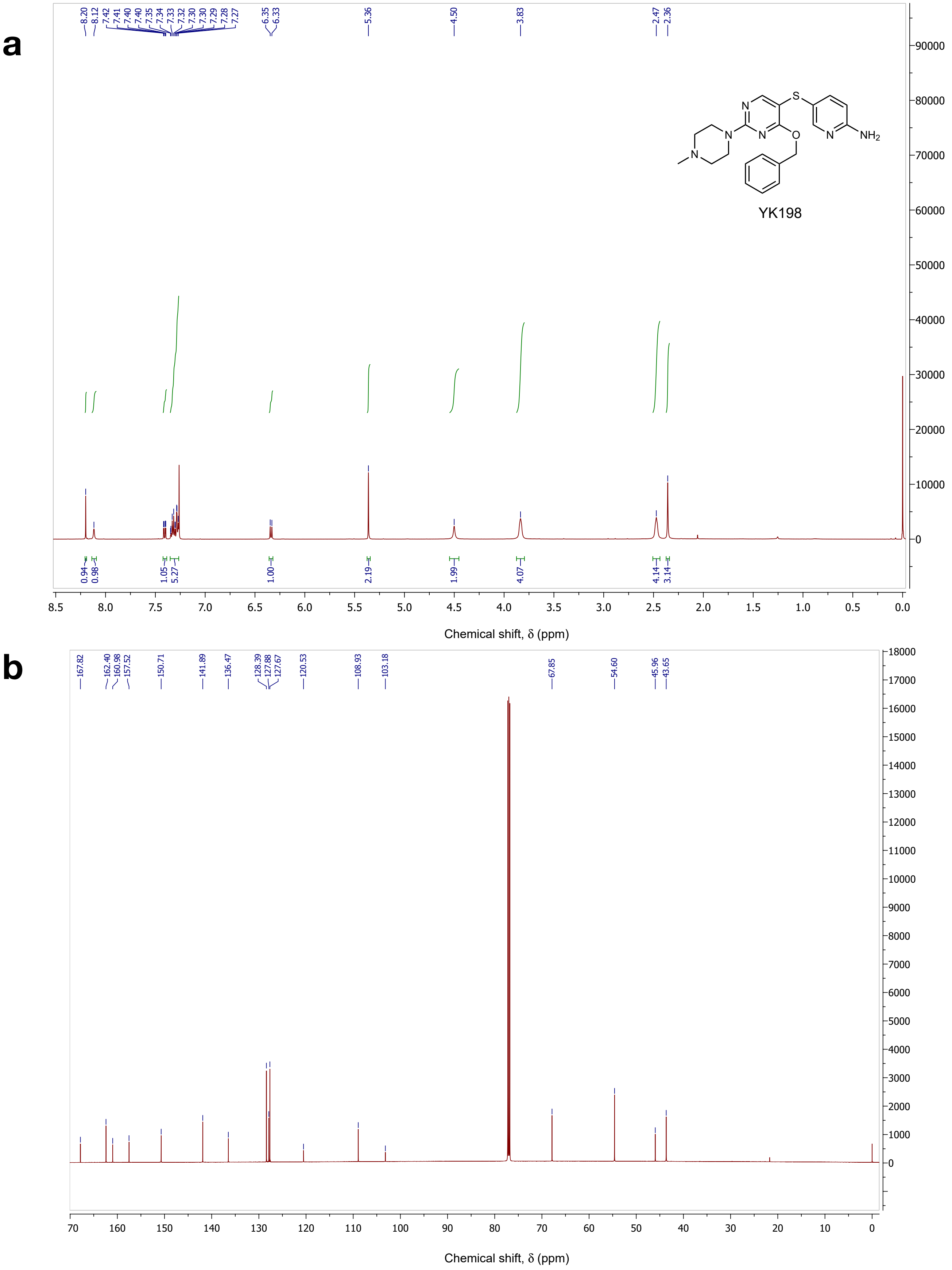

Supplementary Fig. 2. Structural characterization of YK198 by nuclear magnetic resonance spectroscopy. a ^{1}H NMR spectra in $CDCl_3$. **b** ^{13}C NMR spectra in $CDCl_3$.

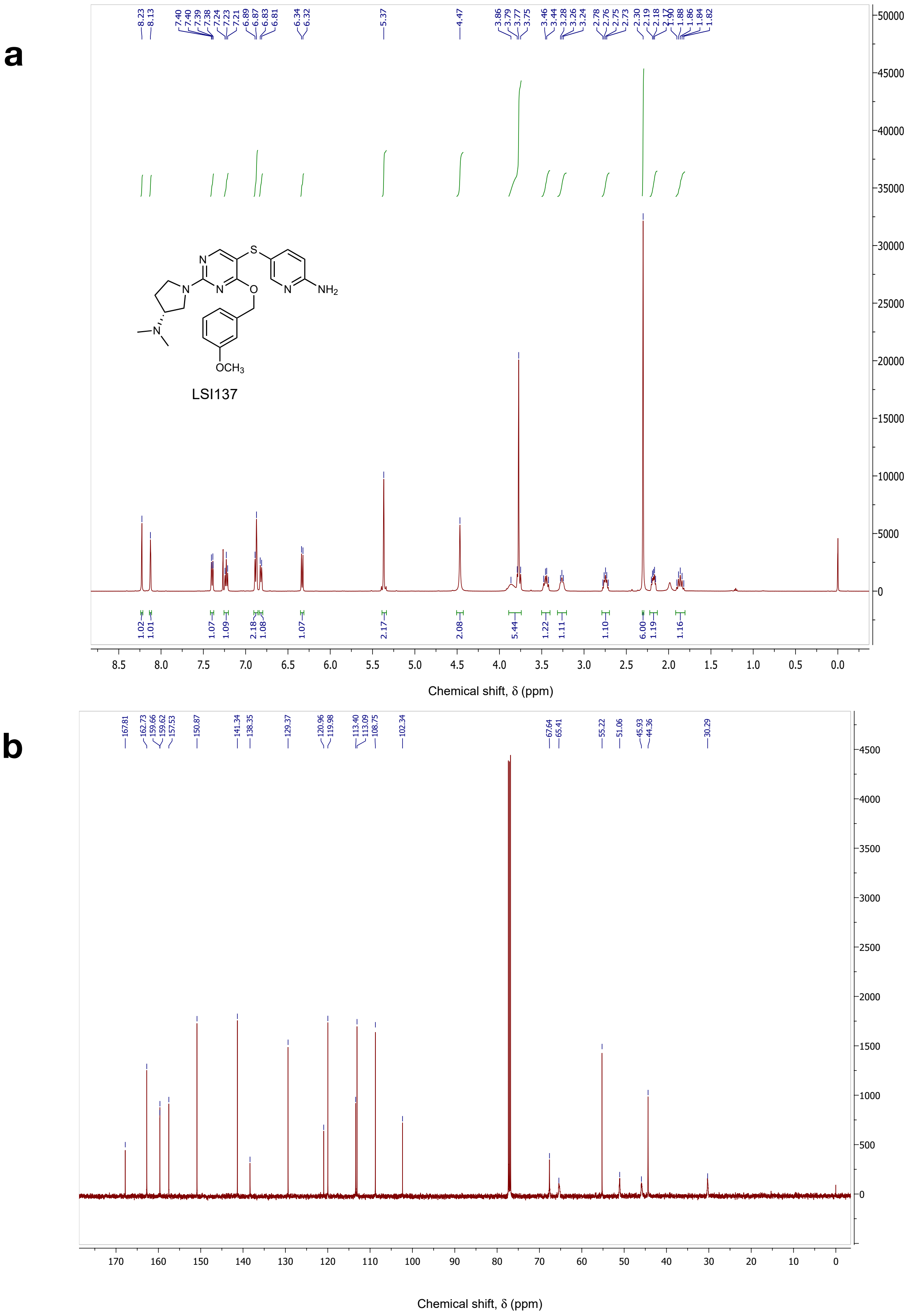

Supplementary Fig. 3. Structural characterization of LSI137 by nuclear magnetic resonance spectroscopy. a ^{1}H NMR spectra in $CDCl_3$. **b** ^{13}C NMR spectra in $CDCl_3$.

a

b

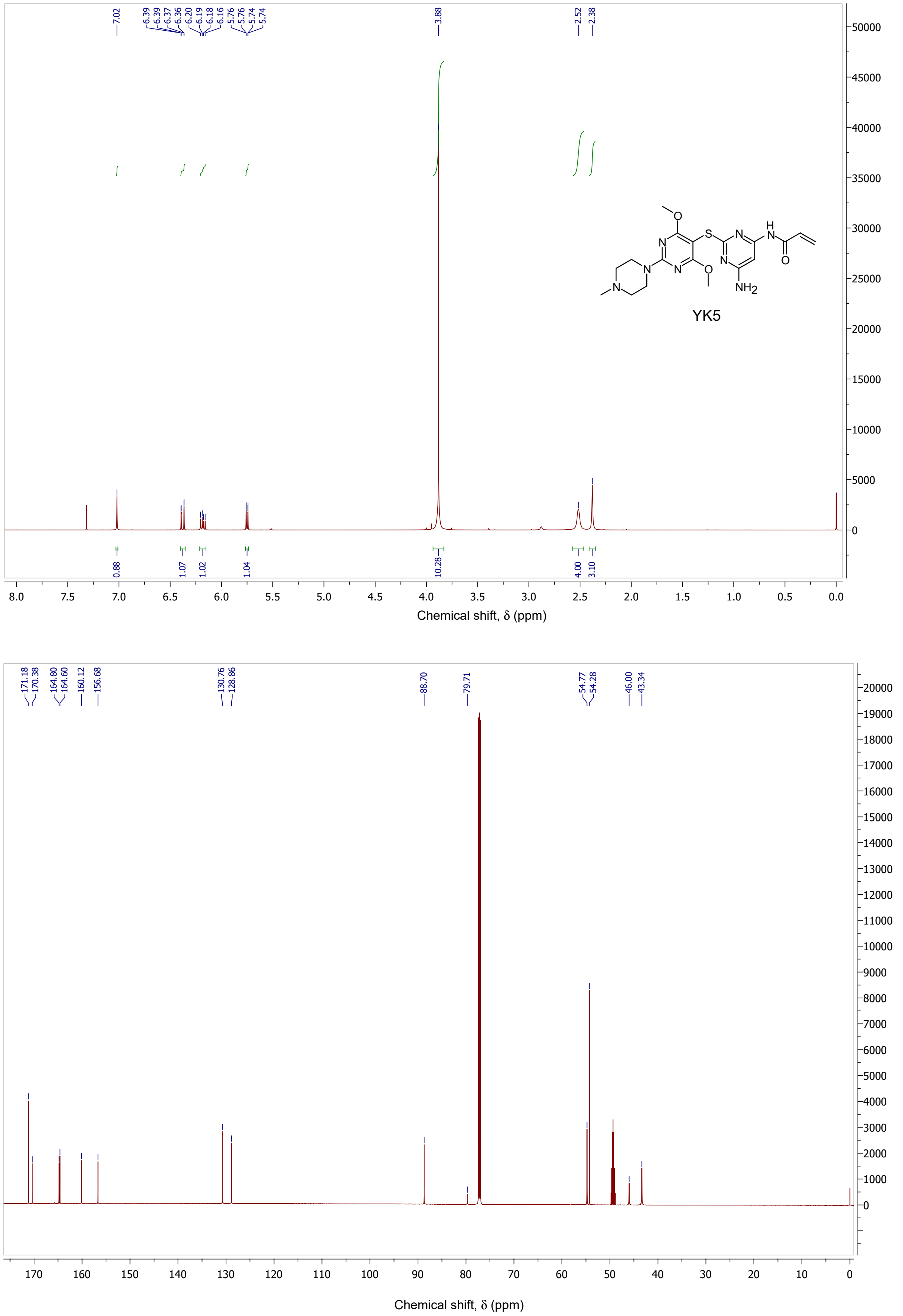

Supplementary Fig. 4. Structural characterization of YK5 by nuclear magnetic resonance spectroscopy. a ^{1}H NMR spectra in $CDCl_3/CD_3OD$. **b** ^{13}C NMR spectra in $CDCl_3/CD_3OD$.

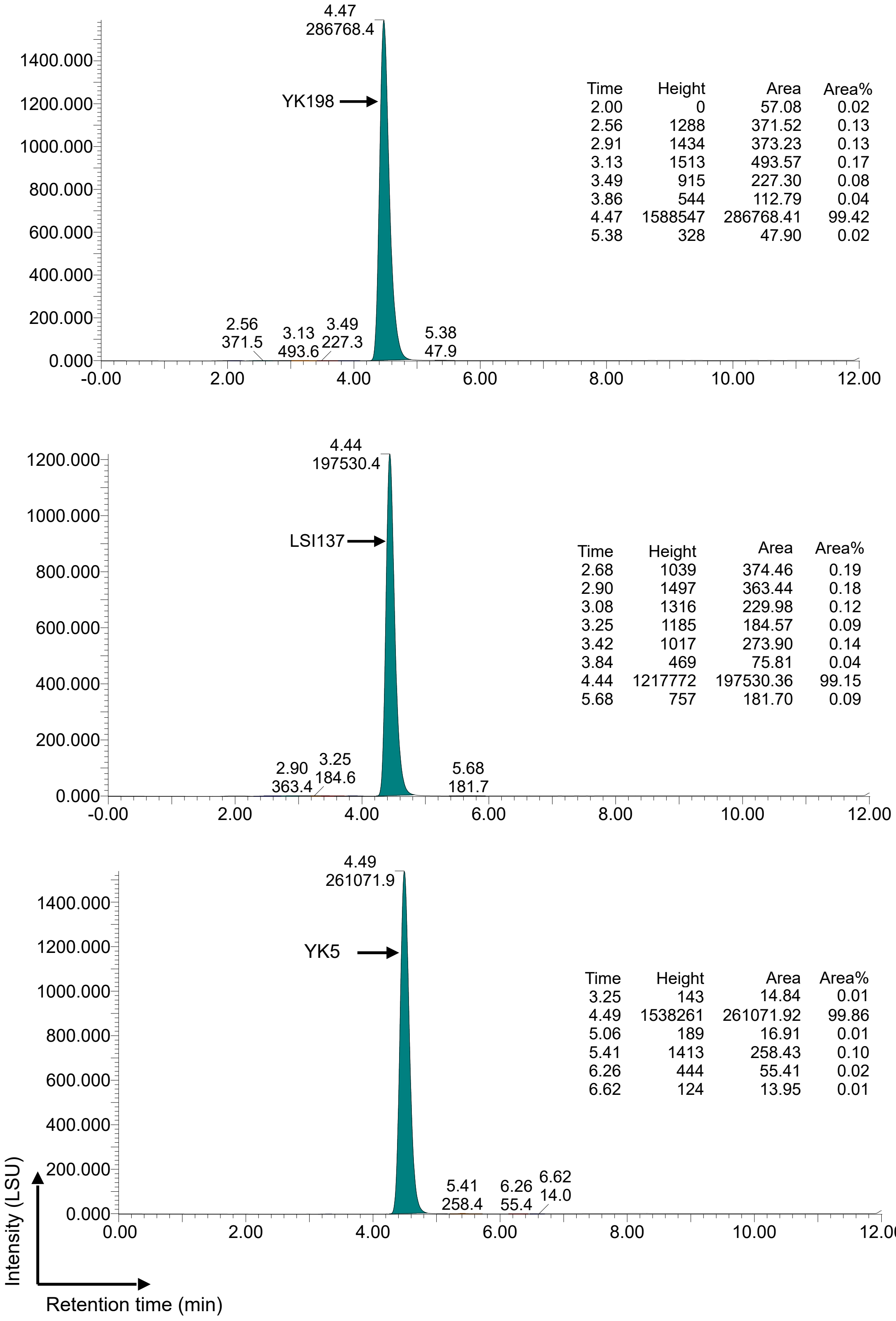

Time	Height	Area	Area%
2.00	0	57.08	0.02
2.56	1288	371.52	0.13
2.91	1434	373.23	0.13
3.13	1513	493.57	0.17
3.49	915	227.30	0.08
3.86	544	112.79	0.04
4.47	1588547	286768.41	99.42
5.38	328	47.90	0.02

Time	Height	Area	Area%
2.68	1039	374.46	0.19
2.90	1497	363.44	0.18
3.08	1316	229.98	0.12
3.25	1185	184.57	0.09
3.42	1017	273.90	0.14
3.84	469	75.81	0.04
4.44	1217772	197530.36	99.15
5.68	757	181.70	0.09

Time	Height	Area	Area%
3.25	143	14.84	0.01
4.49	1538261	261071.92	99.86
5.06	189	16.91	0.01
5.41	1413	258.43	0.10
6.26	444	55.41	0.02
6.62	124	13.95	0.01

Supplementary Fig. 5. HPLC traces for purity evaluation of YK198, LSI137 and YK5. Chromatogram, retention time and peak integration are shown. See Supplementary Note 1 for chromatographic method description.

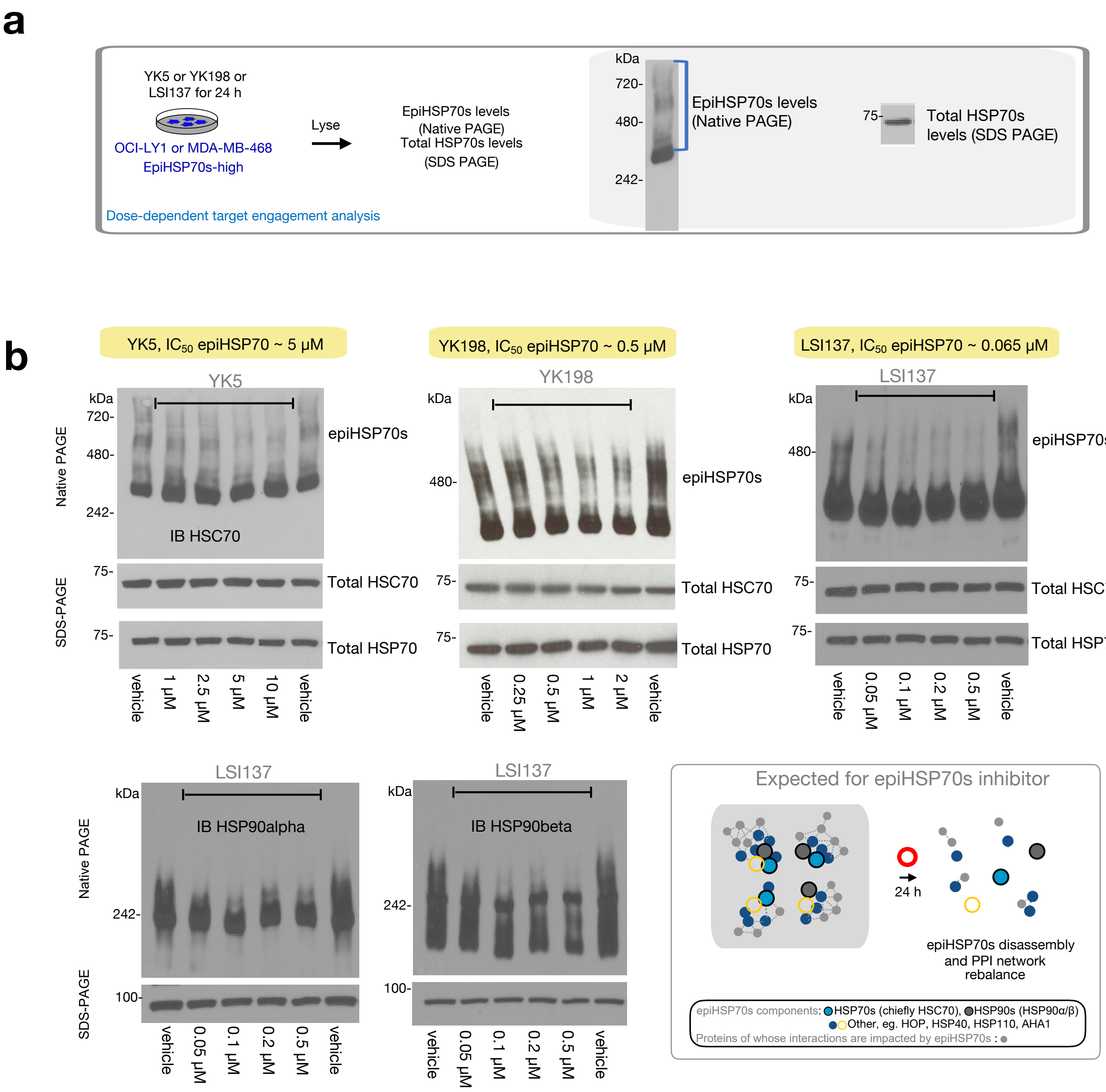

Supplementary Figure 6. Characterization of the epiHSP70s probes.
a Experimental design to evaluate the dose-dependent epiHSP70s target engagement, and in-cell activity, of the YK probes. **b** As in (a) for epiHSP70s and for total HSP70s and HSP90s chaperone levels. Gel images representative of three (n = 3) independent experiments. Graph, individual values (n = 3). HSC70, heat shock cognate 70; HSP90, heat shock protein 90; HOP, HSP-organizing protein; AHA1, activator of Hsp90 ATPase homolog 1; IB, immunoblot; PPI, protein-protein interaction. Source data are provided as a Source Data file.

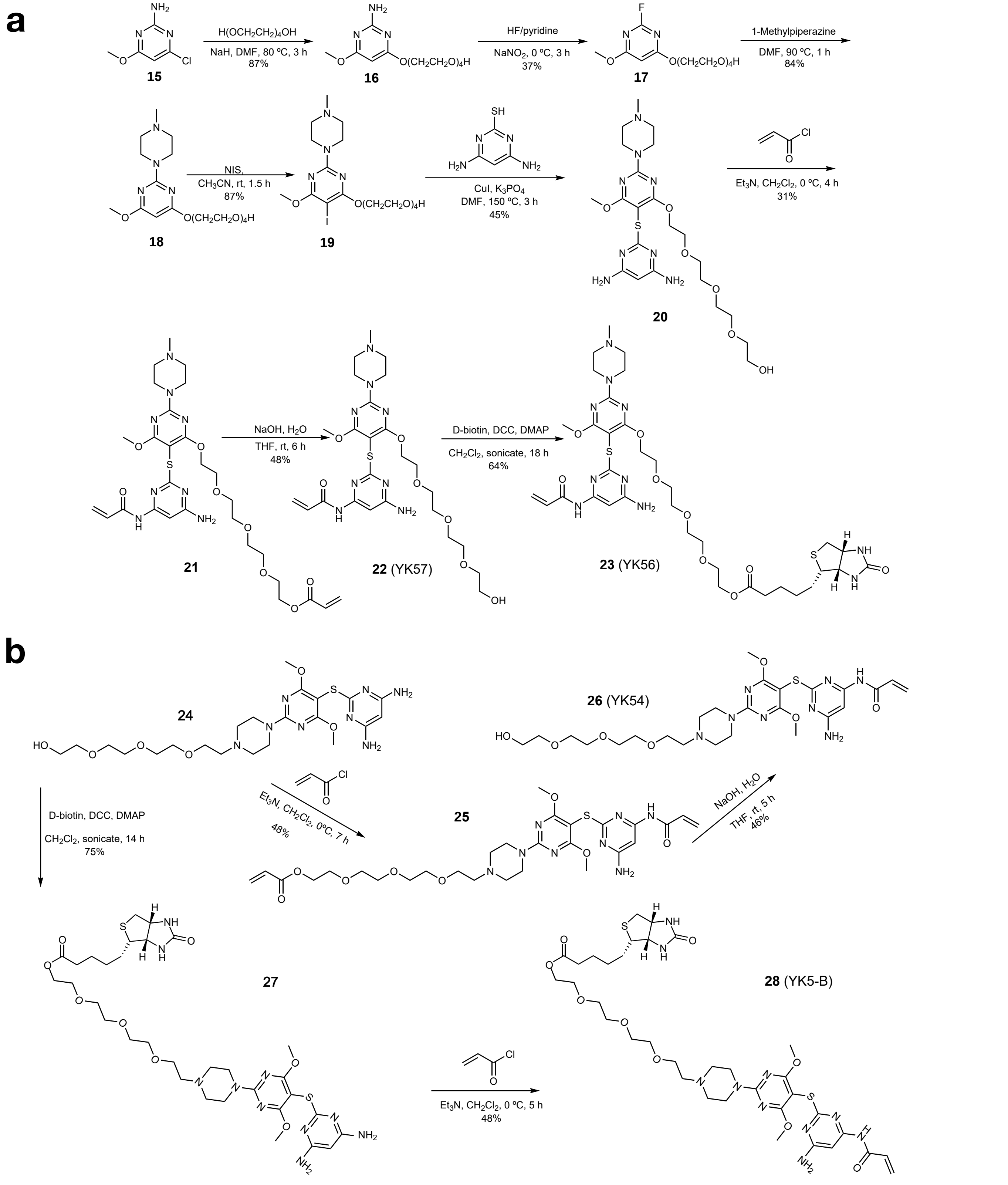

Supplementary Figure 7. Synthetic scheme for the preparation of the biotinylated YK probes and their precursors.

a Synthesis of YK56 and its precursor YK57. **b** Synthesis of YK-5B and its precursor YK54. DCC, N,N'-Dicyclohexylcarbodiimide; DMAP, 4-Dimethylaminopyridine; DMF, Dimethylformamide; NIS, N-Iodosuccinimide; THF, Tetrahydrofuran. See Supplementary Note 1 for detailed description of probe synthesis, purification and characterization.

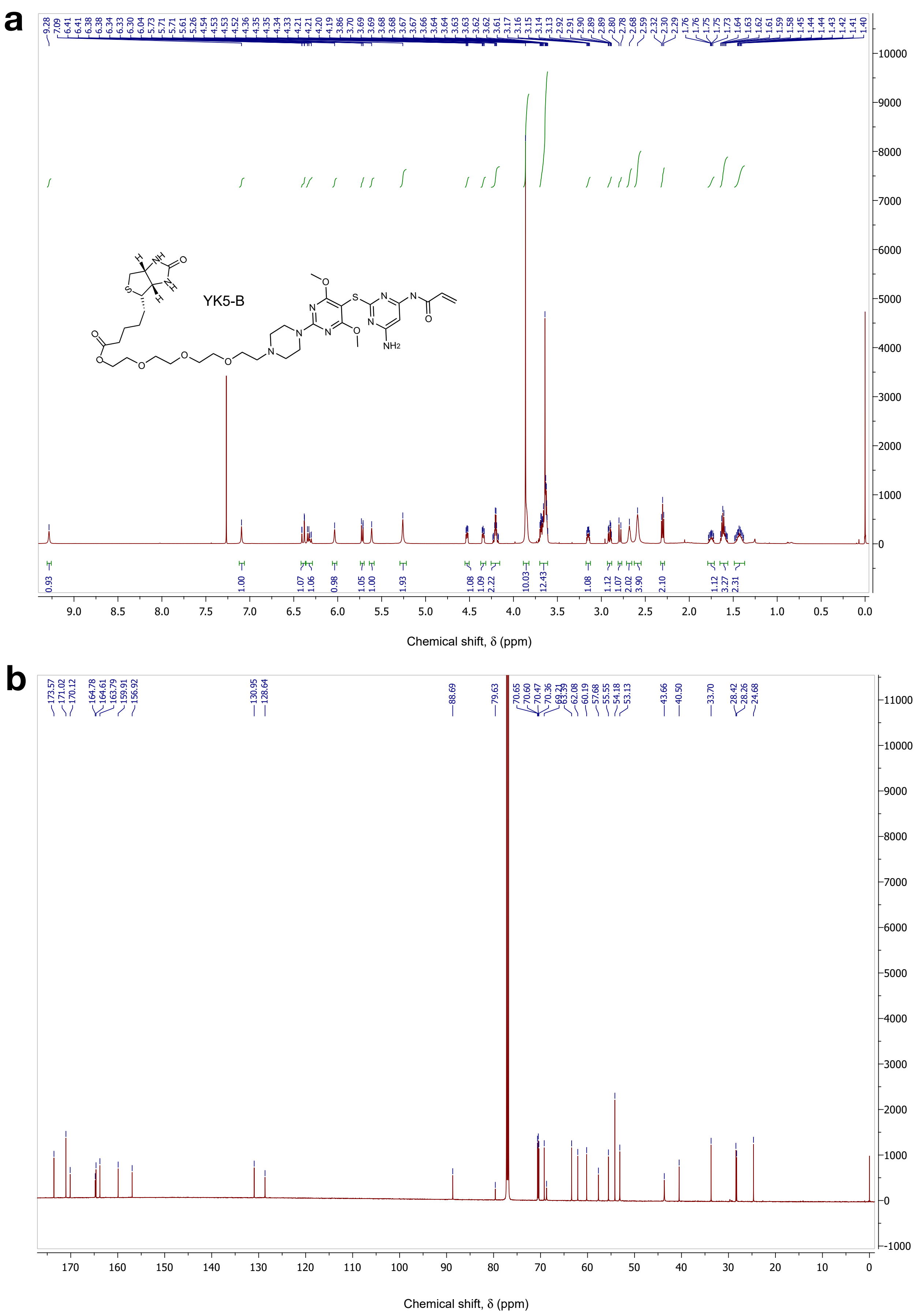

Supplementary Fig. 8. Structural characterization of YK5-B by nuclear magnetic resonance spectroscopy. a ^{1}H NMR spectra in $CDCl_3$. **b** ^{13}C NMR spectra in $CDCl_3$.

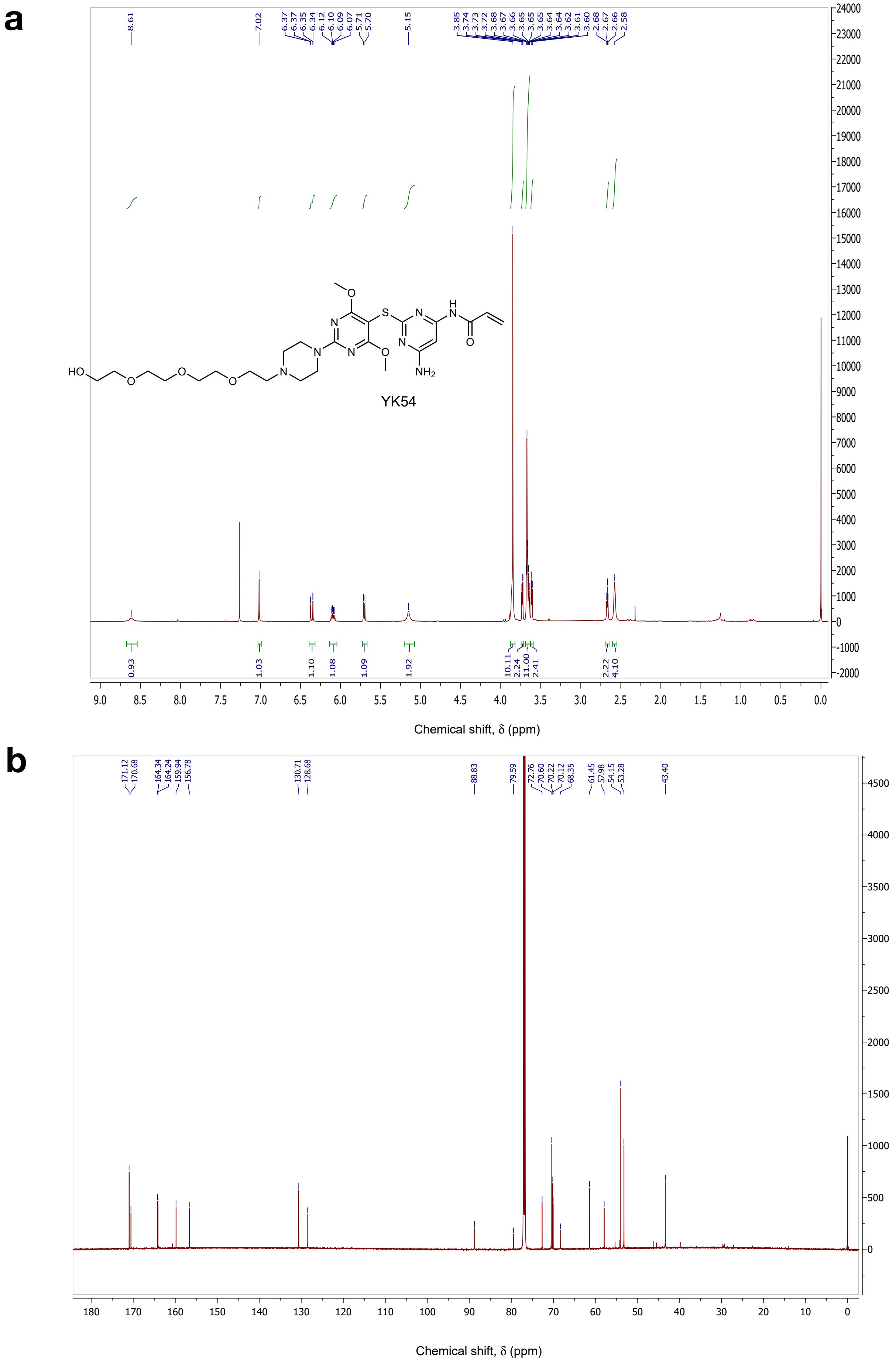

Supplementary Fig. 9. Structural characterization of YK54 by nuclear magnetic resonance spectroscopy. a ^{1}H NMR spectra in $CDCl_3$. **b** ^{13}C NMR spectra in $CDCl_3$.

a

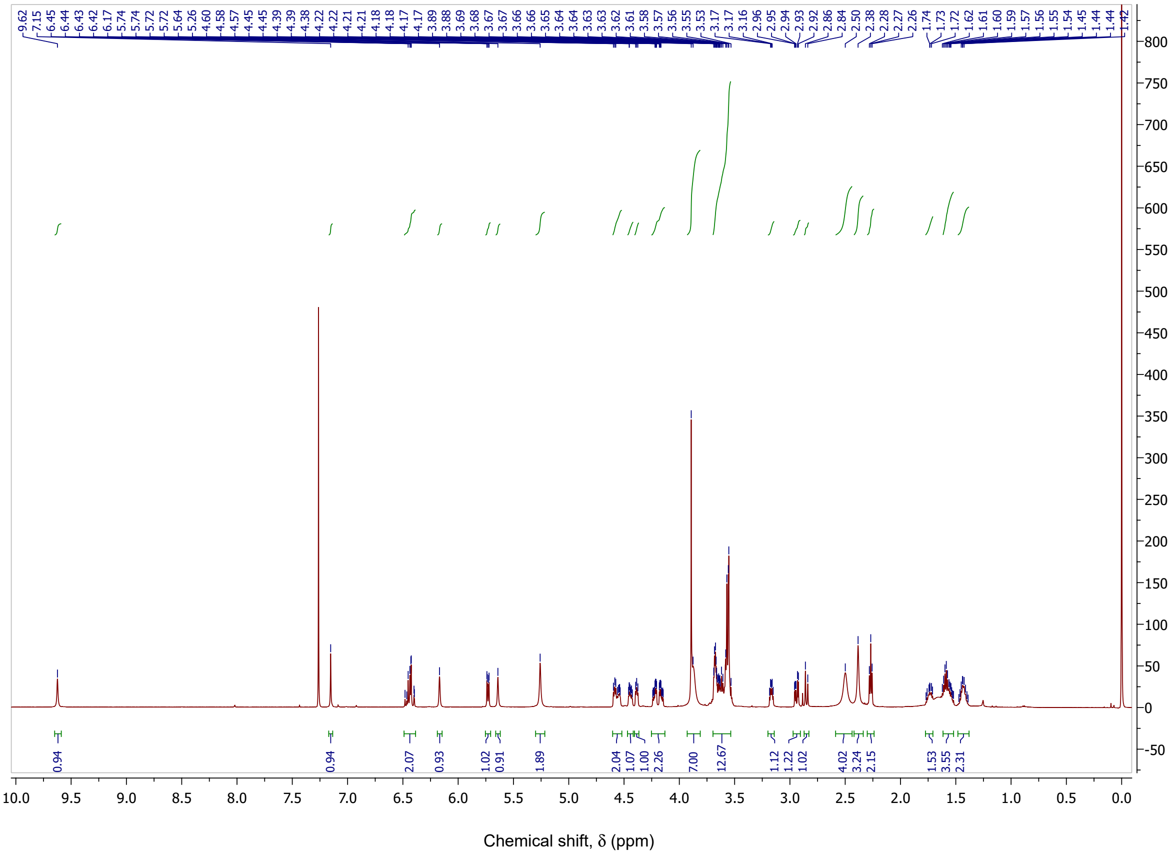

b

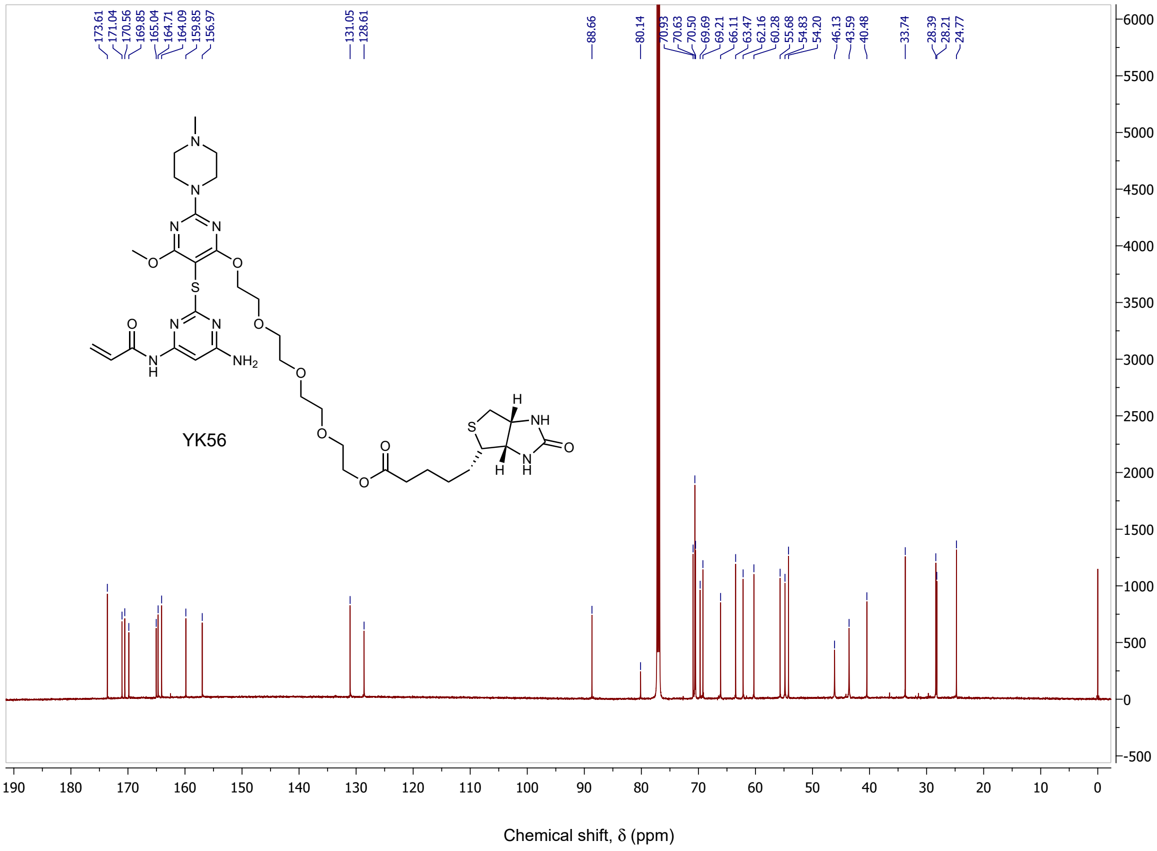

Supplementary Fig. 10. Structural characterization of YK56 by nuclear magnetic resonance spectroscopy. a ^{1}H NMR spectra in $CDCl_3$. **b** ^{13}C NMR spectra in $CDCl_3$.

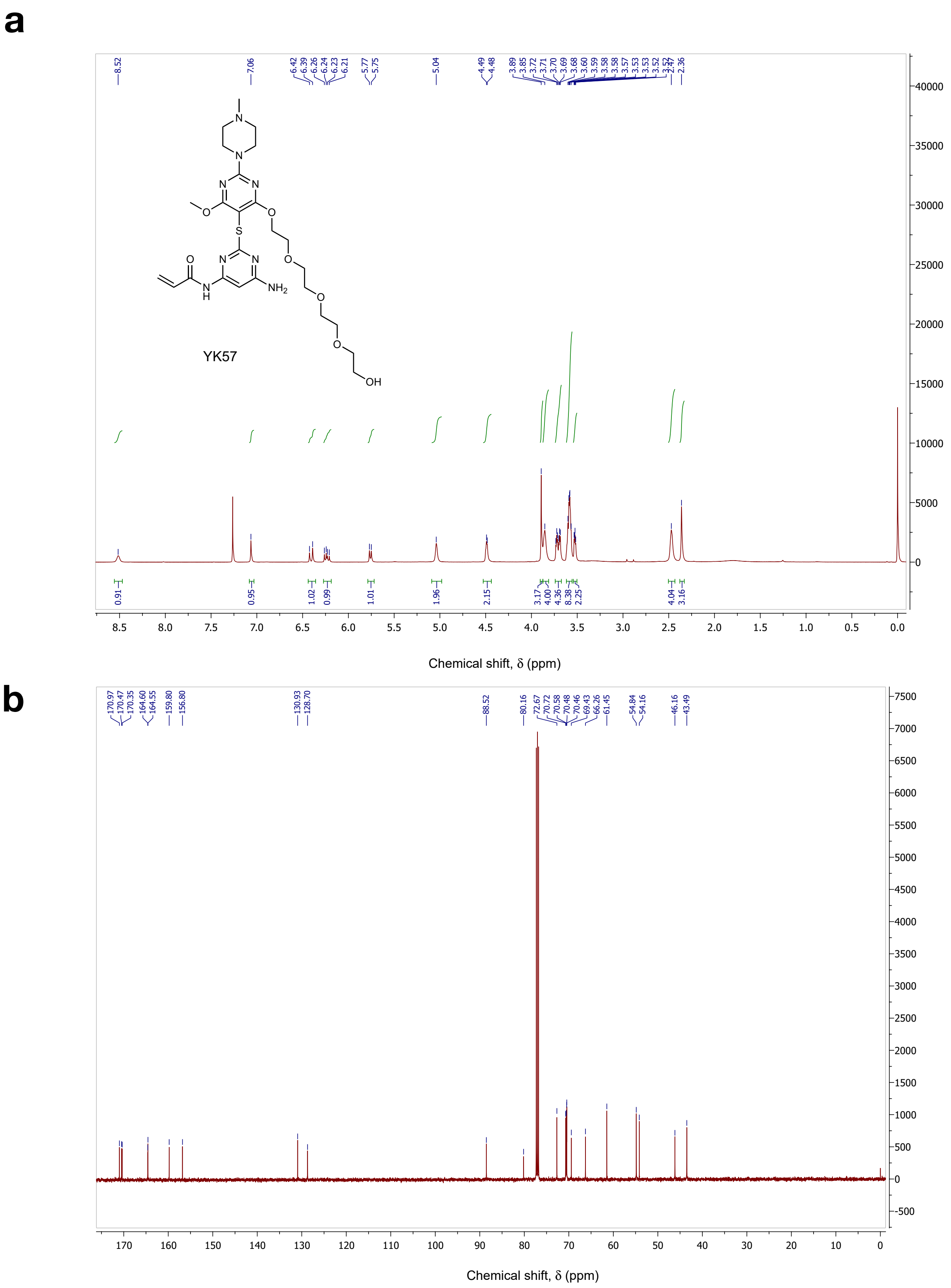

Supplementary Fig. 11. Structural characterization of YK57 by nuclear magnetic resonance spectroscopy. a ^{1}H NMR spectra in $CDCl_3$. **b** ^{13}C NMR spectra in $CDCl_3$.

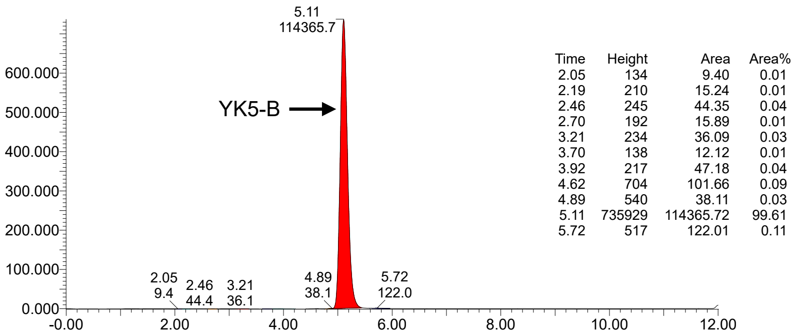

Time	Height	Area	Area%
2.05	134	9.40	0.01
2.19	210	15.24	0.01
2.46	245	44.35	0.04
2.70	192	15.89	0.01
3.21	234	36.09	0.03
3.70	138	12.12	0.01
3.92	217	47.18	0.04
4.62	704	101.66	0.09
4.89	540	38.11	0.03
5.11	735929	114365.72	99.61
5.72	517	122.01	0.11

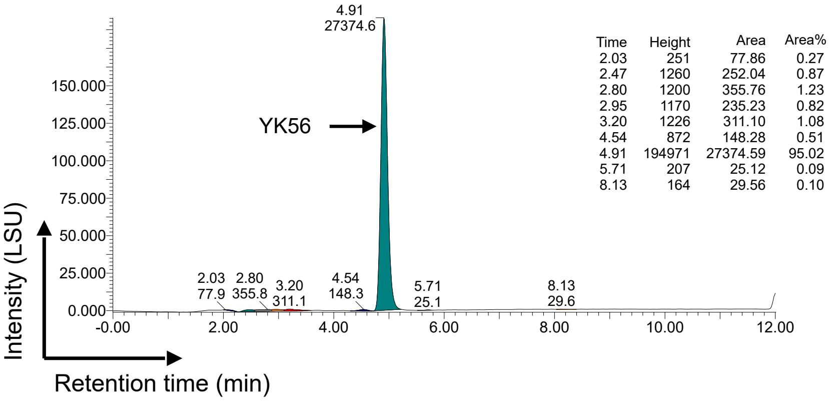

Time	Height	Area	Area%
2.03	251	77.86	0.27
2.47	1260	252.04	0.87
2.80	1200	355.76	1.23
2.95	1170	235.23	0.82
3.20	1226	311.10	1.08
4.54	872	148.28	0.51
4.91	194971	27374.59	95.02
5.71	207	25.12	0.09
8.13	164	29.56	0.10

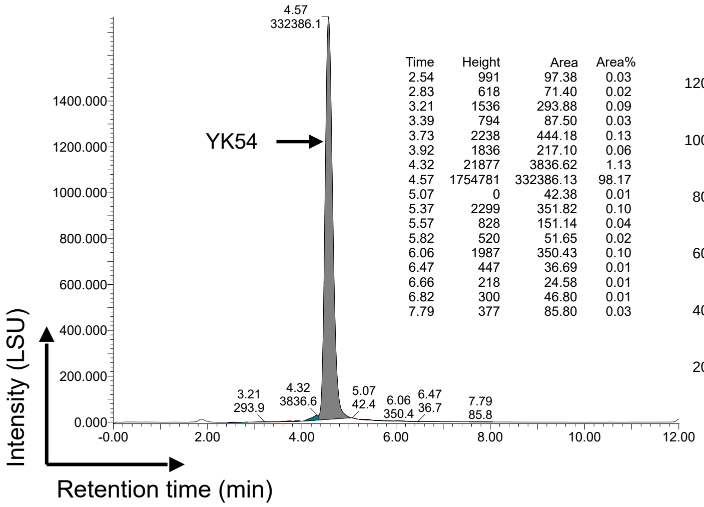

Time	Height	Area	Area%
2.54	991	97.38	0.03
2.83	618	71.40	0.02
3.21	1536	293.88	0.09
3.39	794	87.50	0.03
3.73	2238	444.18	0.13
3.92	1836	217.10	0.06
4.32	21877	3836.62	1.13
4.57	1754781	332386.13	98.17
5.07	0	42.38	0.01
5.37	2299	351.82	0.10
5.57	828	151.14	0.04
5.82	520	51.65	0.02
6.06	1987	350.43	0.10
6.47	447	36.69	0.01
6.66	218	24.58	0.01
6.82	300	46.80	0.01
7.79	377	85.80	0.03

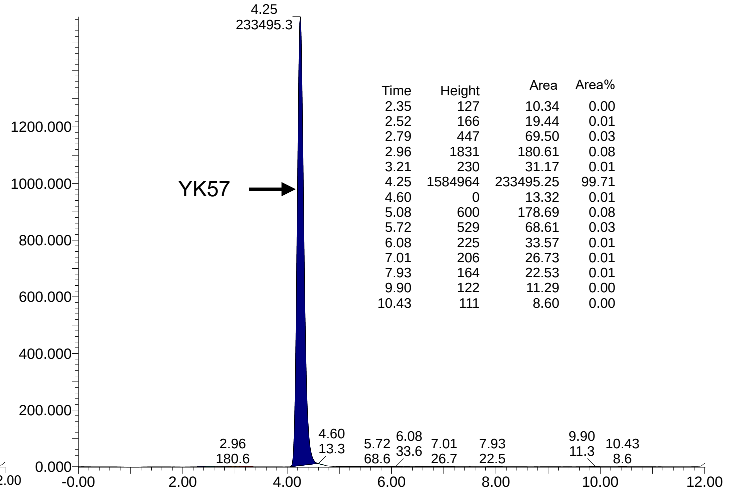

Time	Height	Area	Area%
2.35	127	10.34	0.00
2.52	166	19.44	0.01
2.79	447	69.50	0.03
2.96	1831	180.61	0.08
3.21	230	31.17	0.01
4.25	1584964	233495.25	99.71
4.60	0	13.32	0.01
5.08	600	178.69	0.08
5.72	529	68.61	0.03
6.08	225	33.57	0.01
7.01	206	26.73	0.01
7.93	164	22.53	0.01
9.90	122	11.29	0.00
10.43	111	8.60	0.00

Supplementary Fig. 12. HPLC traces for purity evaluation of biotinylated YK probes YK5-B and YK56, and of their precursor molecules YK54 and YK57. Chromatogram, retention time and peak integration are shown. See Supplementary Note 1 for chromatographic method description.

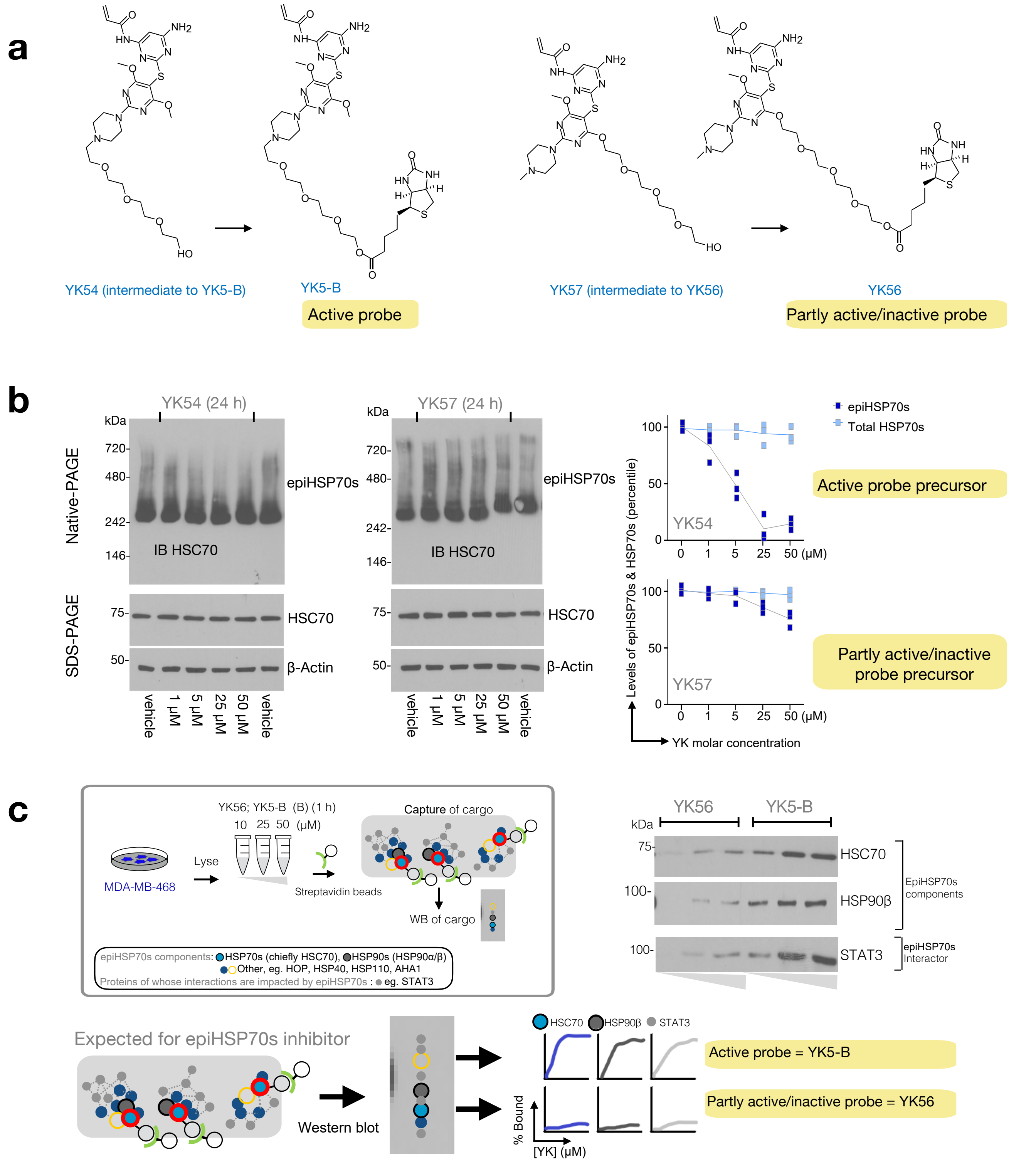

Supplementary Figure 13. Evaluation of positive and negative (less active) controls used in affinity-purification experiments.
a Chemical structure of the YK affinity probes and their pre-biotin precursors. **b** Dose-dependent, in-cell activity, of the YK probes on epiHSP70s disassembly. Experimental design as in Supplementary Fig. 1a. Data are presented as mean, n = 3. **c** EpiHSP70s components (eg. HSC70, HSP90β) and epiHSP70s-interacting proteins (STAT3) (see Fig. 2 and text, why STAT3) captured by the probes in MDA-MB-468 cancer cells, as indicated. Gel images representative of three (n = 3) independent experiments. Source data are provided as a Source Data file.

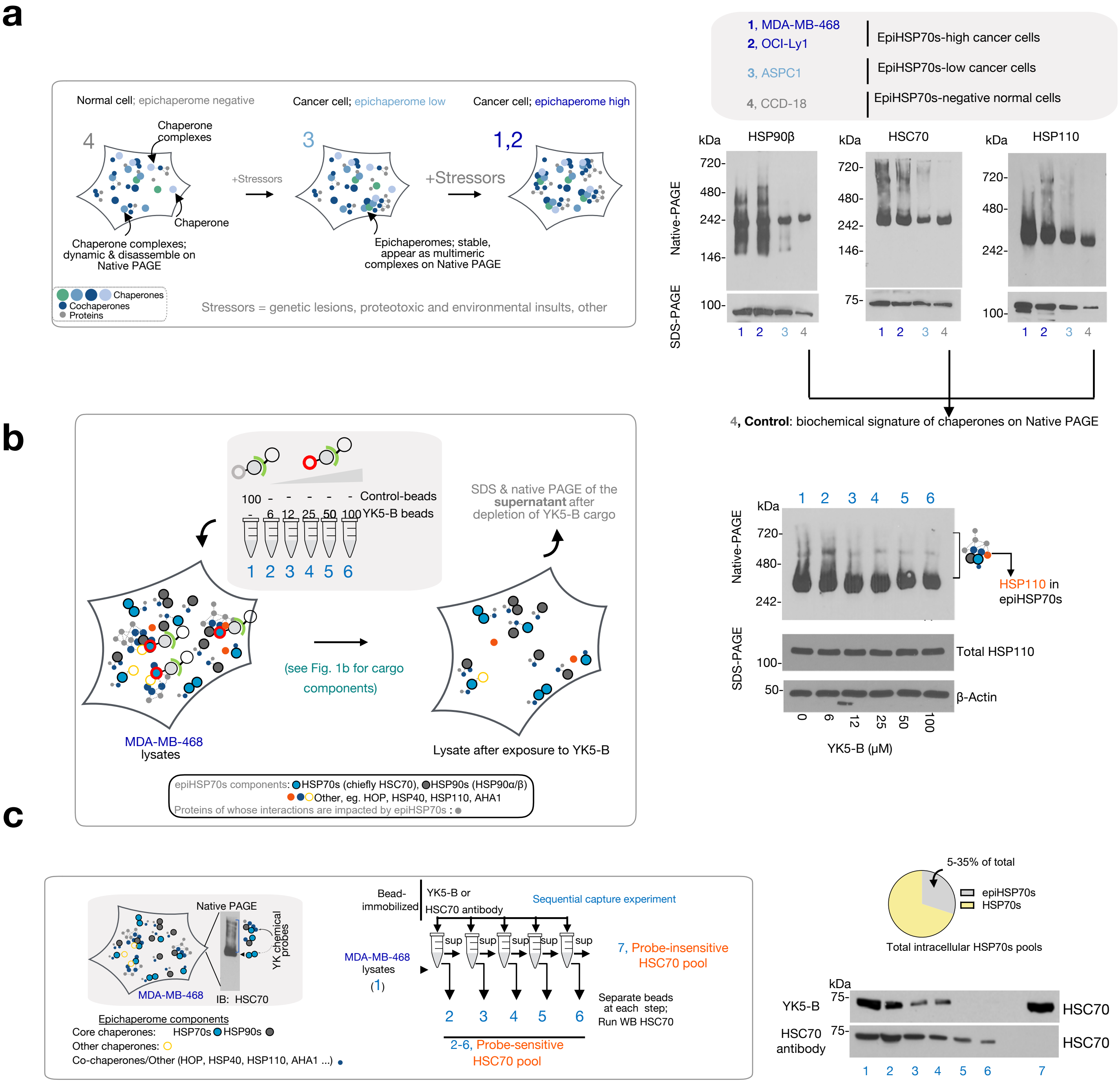

Supplementary Figure 14. Evaluation of epiHSP70s probes through affinity capture in epiHSP70s-high cancer cells.
a Biochemical evaluation of epichaperome levels through Native PAGE followed by immunoblotting. Total levels of indicated chaperones were analyzed by Western blot. Same total protein amount was loaded for both runs. **b** The levels of epiHSP70s-resident HSP110 evaluated in homogenates subjected to one application of YK5-B beads loaded with indicated amounts of YK5-B. Beads were generated by incubating YK5-B (0 to 100 μM) with streptavidin-immobilized beads. Gel images representative of three (n = 3) independent experiments. β-Actin, protein loading control. Related to Figure 1b. **c** Affinity purifications performed in the indicated cell homogenates, with sequential applications of a set concentration of solid-support immobilized YK5-B or HSC70 antibody. Gel images representative of three (n = 3) independent experiments. Source data are provided as a Source Data file.

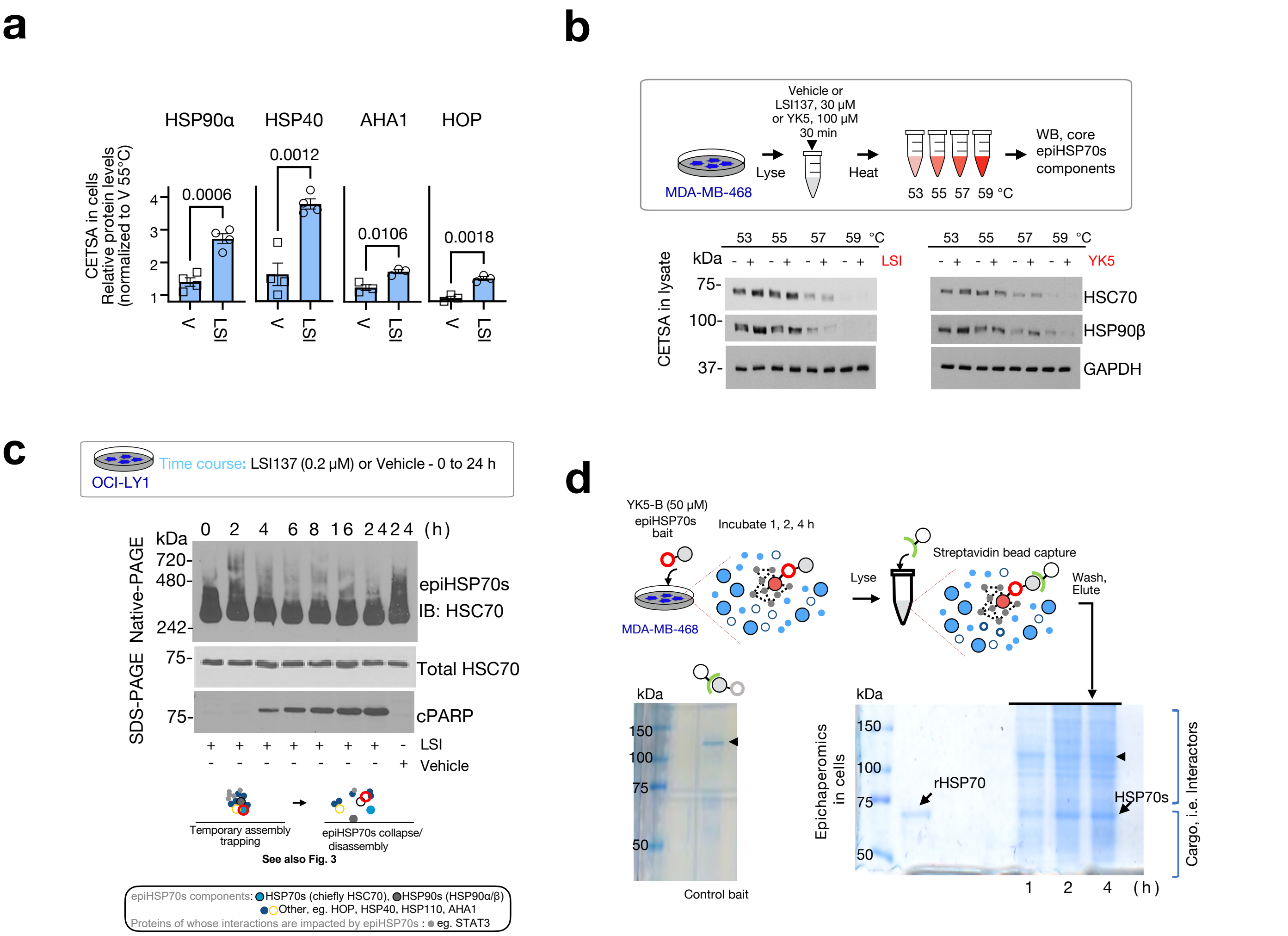

Supplementary Figure 15. Biochemical mechanism of YK-probes.
a CETSA as in Figure 2a-c. Homogenates from MDA-MB-468 cells treated with vehicle (DMSO) or LSI137, 10 μM. Data are presented as mean ± s.e.m., vehicle (V) and LSI137 (LSI), n = 4 for HSP90α and HSP40 and n = 3 for AHA1 and HOP; unpaired two-tailed t-test. Values, protein levels at 53°C normalized to those obtained for vehicle at 55°C. **b** CETSA assay performed with YKs added to cell homogenates, as indicated. Gel images are representative of three independent experiments. **c** Time-dependent analysis of epiHSP70s levels in epiHSP70s high OCI-LY1 cells treated with LSI137 as indicated. Total HSC70 levels and induction of apoptosis (detected by PARP cleavage, cPARP were analyzed by Western blot. Gel images are representative of three independent experiments. **d** Time-dependent analysis of YK5-B binding. For compounds such as YK5 where irreversible binding plays a role, binding consists of two components, one reflecting reversible binding and another reflecting the subsequent covalent binding, and depends on the extent to which the covalent interaction has occurred. Indeed, incubation of cells for 1, 2, 4 h with YK5-B led to a progressive increase in the amount of immobilized epiHSP70. Coomassie blue stained SDS-PAGE of the eluted interactors captured by epichaperomics as shown in the schematic. Left gel: control beads cargo is presented to indicate the location of contaminants. A contaminant(s) at ~120kDa is shown with a black arrowhead. rHSP70, recombinant HSP70 loaded as control. Gel images are representative of three independent experiments. Source data are provided as Source data files.

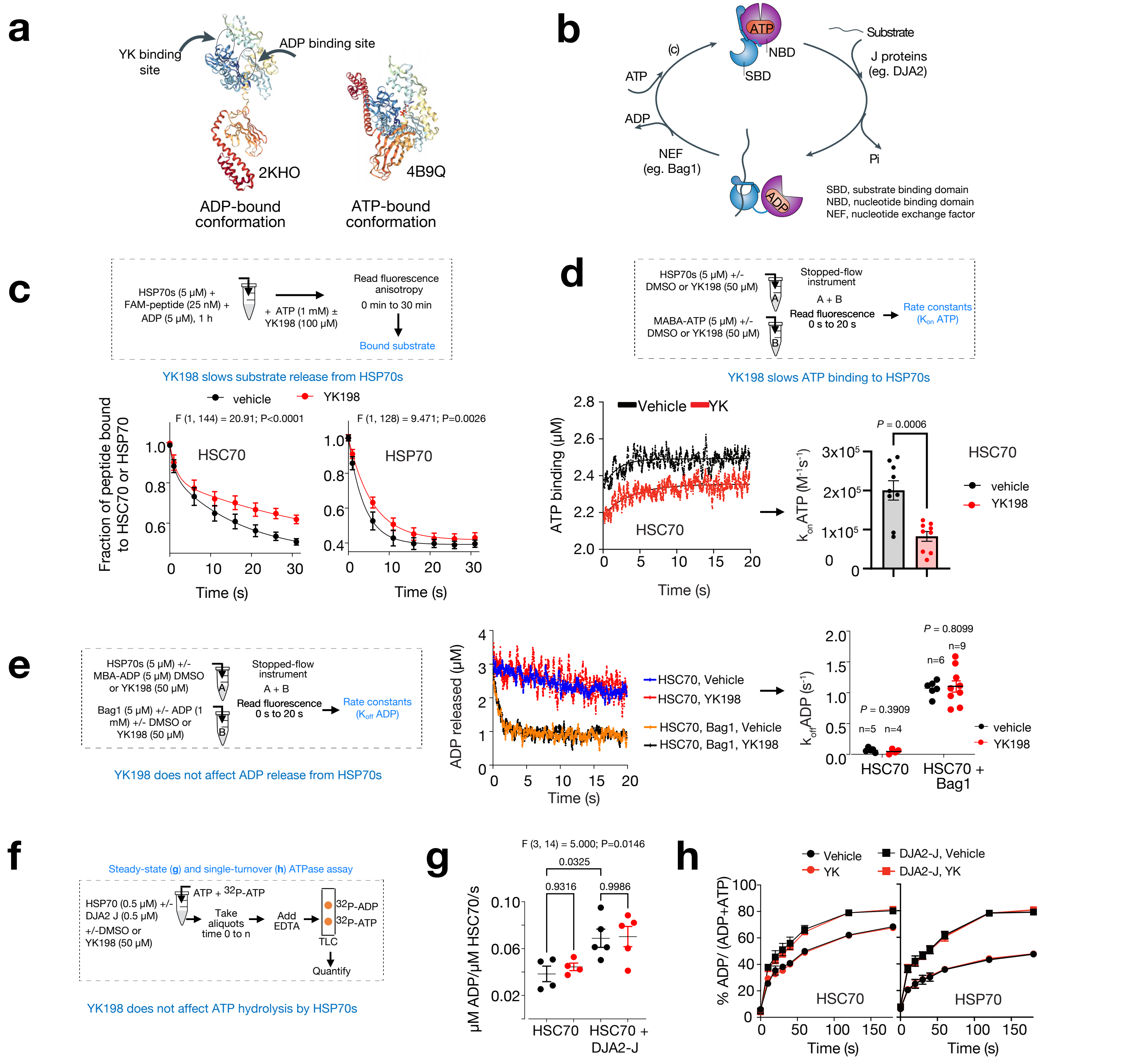

Supplementary Fig. 16. The effect of YKs on HSP70s in reconstituted systems.
a ATP- and ADP-binding mediate conformations of HSP70s, with ATP binding inducing a conformational change that enables interactor protein (i.e., substrate) release. Substrate is delivered to HSP70s in the ATP-bound conformation. Hydrolysis of ATP to ADP, accelerated by J-proteins, such as DJA2, results in HSP70s conformational change leading to tight binding of the substrate. Nucleotide exchange factors, such as Bag1, then induce ADP dissociation and rebinding of ATP, leading to substrate release. **b** Crystal structures of HSP70 in the ADP- and ATP-bound conformation (PDB:2KHO, Structure of *E. coli* HSP70 (DNAK) chaperone complexed with ADP and substrate, PDB DOI: https://doi.org/10.2210/pdb2KHO/pdb; PDB:4B9Q, Open conformation of ATP-bound HSP70 homolog DnaK, PDB DOI: https://doi.org/10.2210/pdb4B9Q/pdb). The binding site of YKs is shown in rapport to that occupied by ADP. **c** The effect of YK198 on substrate binding was determined as indicated. Graph, mean±sem, n = 10 and n = 9 for HSC70 and HSP70, respectively, two-way ANOVA. **d** The effect of YK198 on ATP on-rate. Graph, mean ± s.e.m., n = 9, unpaired two-tailed t-test. **e** The effect of YK198 on ADP off-rates in the presence or absence of a nucleotide exchange factor (eg. Bag1). Graph, mean±sem, unpaired two-tailed t-test. **f** Steady-state and single-turnover ATPase rates were measured for reactions with HSC70 or HSP70 and DJA2-J (DJA2 J-domain) with vehicle or YK198, as indicated. **g** Graph, mean±sem, one-way ANOVA, n=4 and n=5 for HSC70 and HSC70+DJA2-J, respectively. **h** Graph, mean±sem, n=3 for HSC70 +YK198, HSC70+DJA2-J+YK198, HSP70+DJA2-J and HSP70+DJA2-J+YK198 and n=4 for all others. Two-way ANOVA with Tukey's post-hoc. Source data, along with statistical analyses and statistics output, are provided as Source data file.

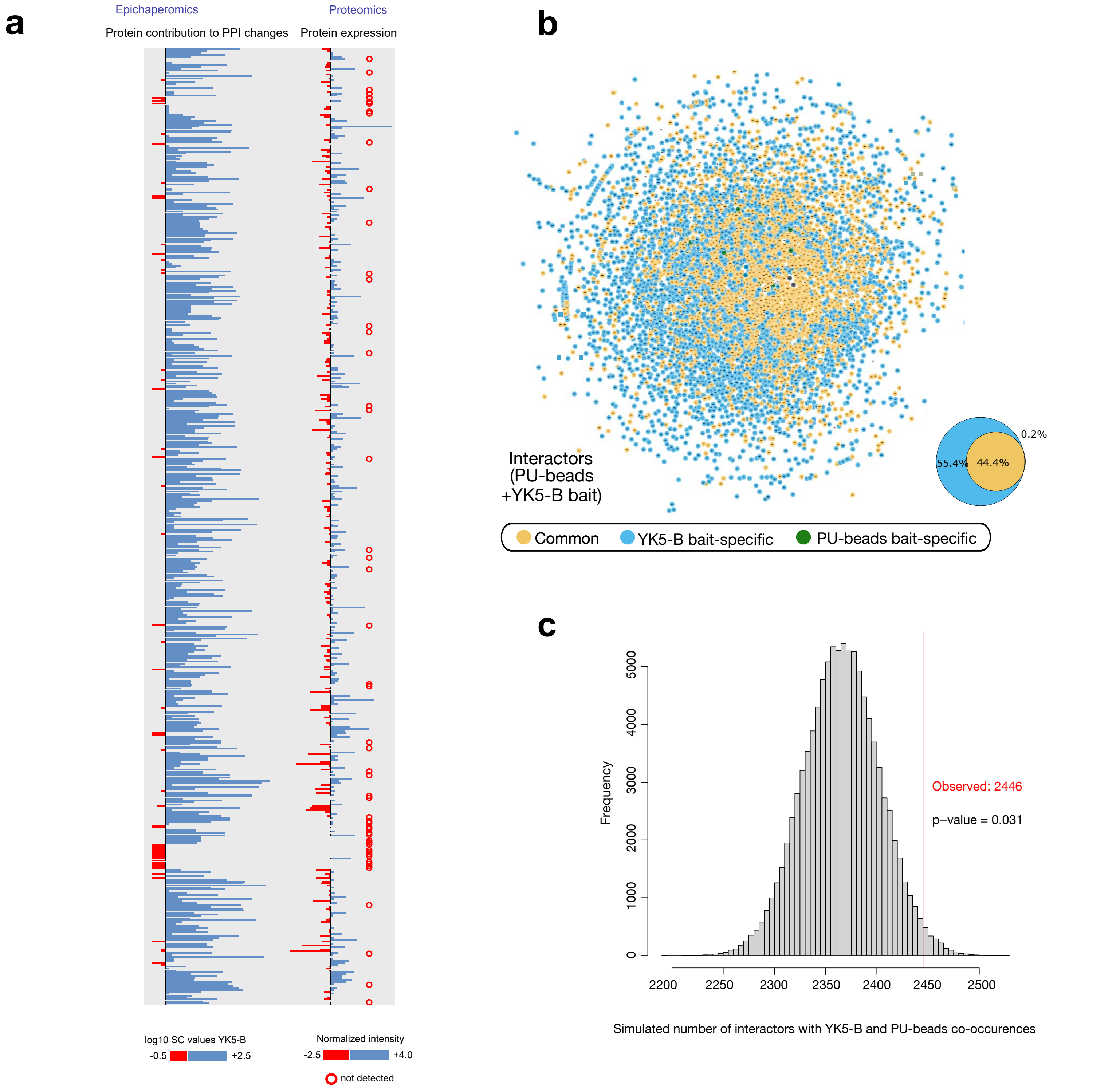

Supplementary Figure 17. Analysis of the epiHSP70s and epiHSP90s interactors.
a Protein connectivity, as detected by YK5-B epichaperomics, compared to protein levels, as determined by quantitative proteomics in MDA-MB-468 cells (see also Supplementary Data 1). SC, spectral counts. **b** Topology of the interactors identified by the YK5-B beads and PU-beads in the MDA-MB-468 cancer cells. The map shows common epiHSP70s and epiHSP90s interactors (yellow), those epiHSP70s specific (blue), and those epiHSP90s-specific (green). Venn diagram, %common and unique interactors as identified by the individual baits. **c** The histogram of the simulated YK5-B beads and PU-beads interactors under null hypothesis of epiHSP70s-epiHSP90s independence, where the mean is 2366 with 95% confidence interval (2293, 2439). The observed number of epiHSP70s-epiHSP90s co-occurrences, displayed as the red vertical line, was 2446, which indicates a statistically significant positive correlation between interactors (exact two-sided p-value 0.031). Thus, the null hypothesis of epiHSP70s-epiHSP90s independence is rejected at the significance level of 0.05. Source data are provided as Supplementary Data files.

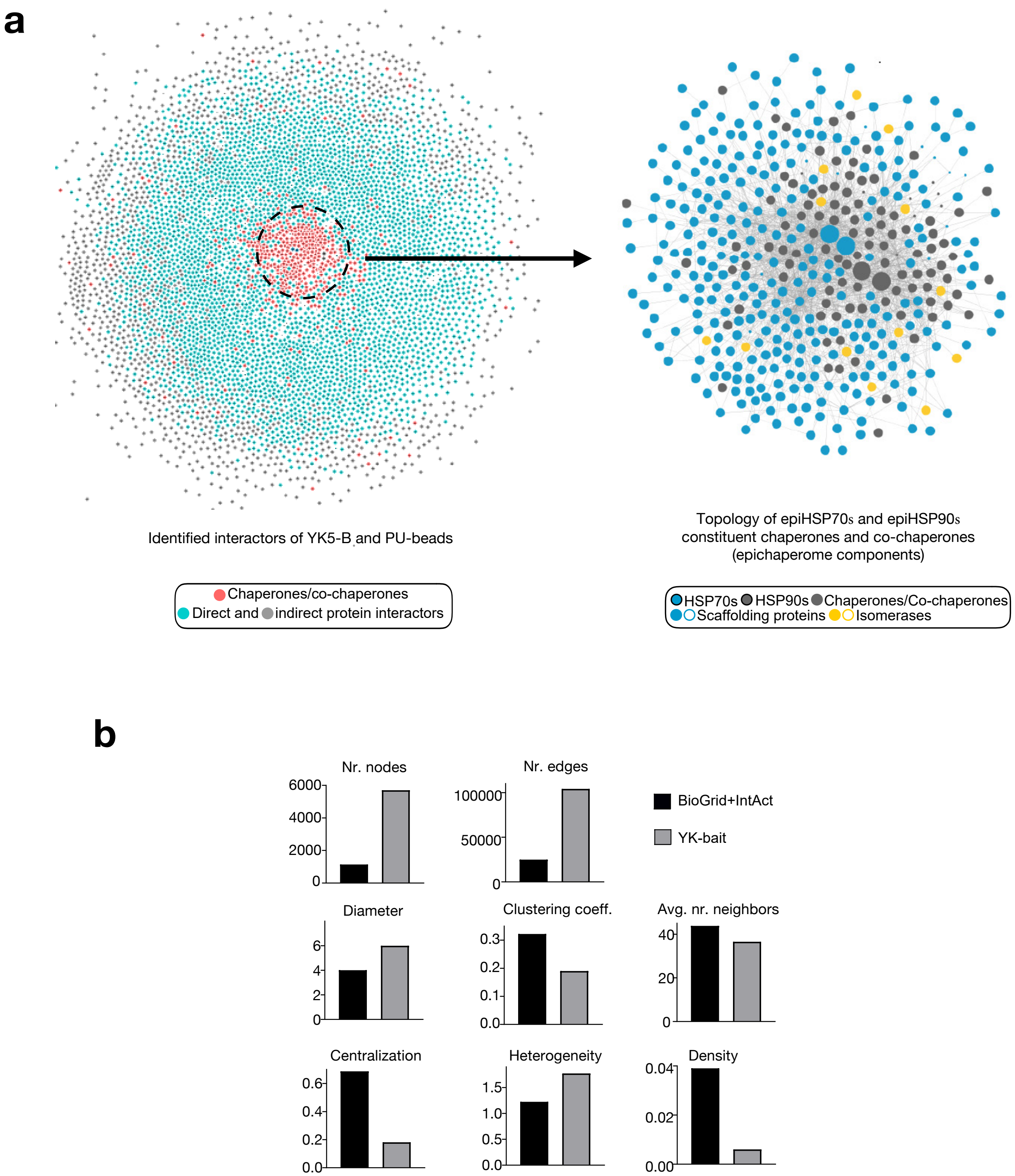

Supplementary Figure 18. Topology of interactors identified by epichaperomics.
a Topology of the interactors identified by the YK5-B beads and PU-beads in the MDA-MB-468 cancer cells (see Figure 5b). The map shows the distribution of epichaperome components in relation to direct and indirect epichaperome interactors. **b** Calculated network topology parameters for the PPI networks created for the YK5-B interactors and for the HSP70s interactors available from indicated databases. Source data are provided as Supplementary Data files.

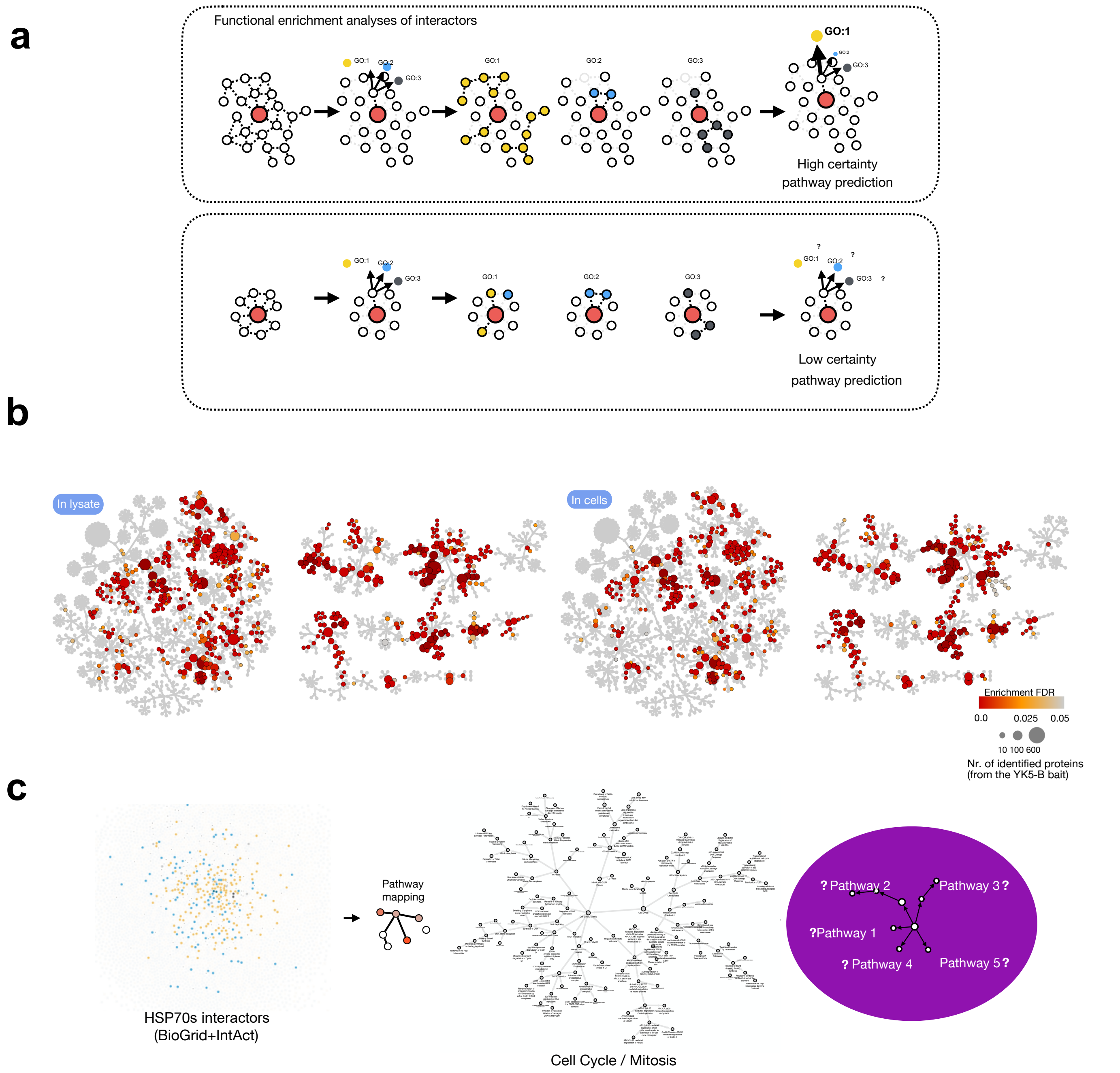

Supplementary Figure 19. Determination of context-specific functions of proteins.
a A single protein may carry out different functions with different partners in different biological contexts. It is the number of disease-module associated proteins, not the number of proteins identified by a specific method, that is of critical importance for accurate mapping of dysregulated protein pathways underlying disease phenotypes. **b** Functional annotation (Reactome mapping) of epiHSP70s interactors identified by the YK5-B beads in MDA-MB-468 cells. See Figure 5a for experimental design. **c** Reactome mapping of the reported HSP70s interactors shows the difficulty of making correct context-specific functional annotations for a protein when insufficient disease-module associated proteins are identified. The functional mapping of cell cycle related proteins is shown. Source data are provided as Supplementary Data files.

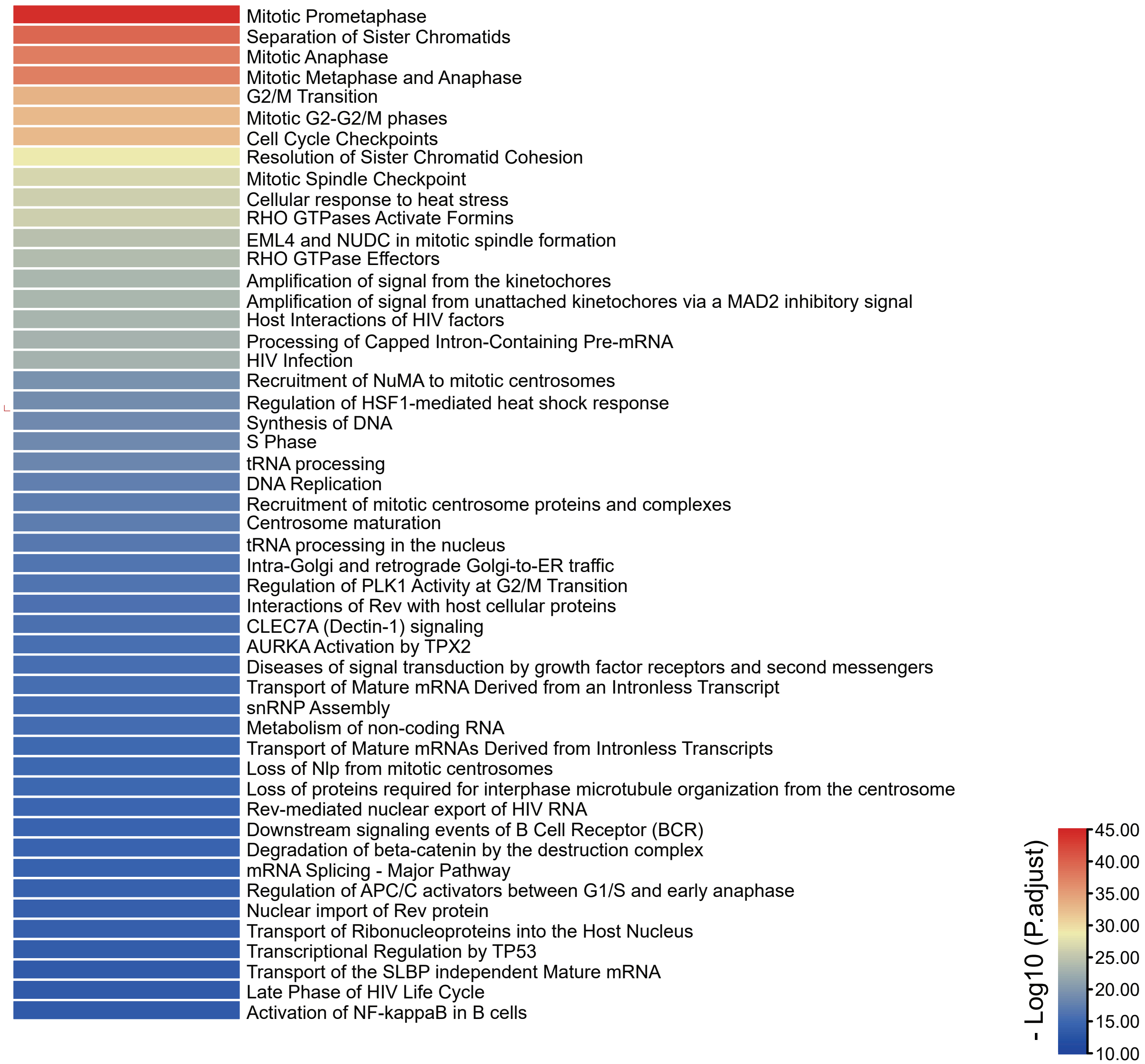

Supplementary Figure 20. Reactome functional mapping of Grade A interactors identified by the epiHSP70s bait.
Output from the Reactome pathway enrichment analysis detailing the functional annotation of Grade A proteins. Top 50 pathways ranked by the p.adjust values are shown. Size bar, negative log10 (p-value). Source data are provided as Supplementary Data files.

a

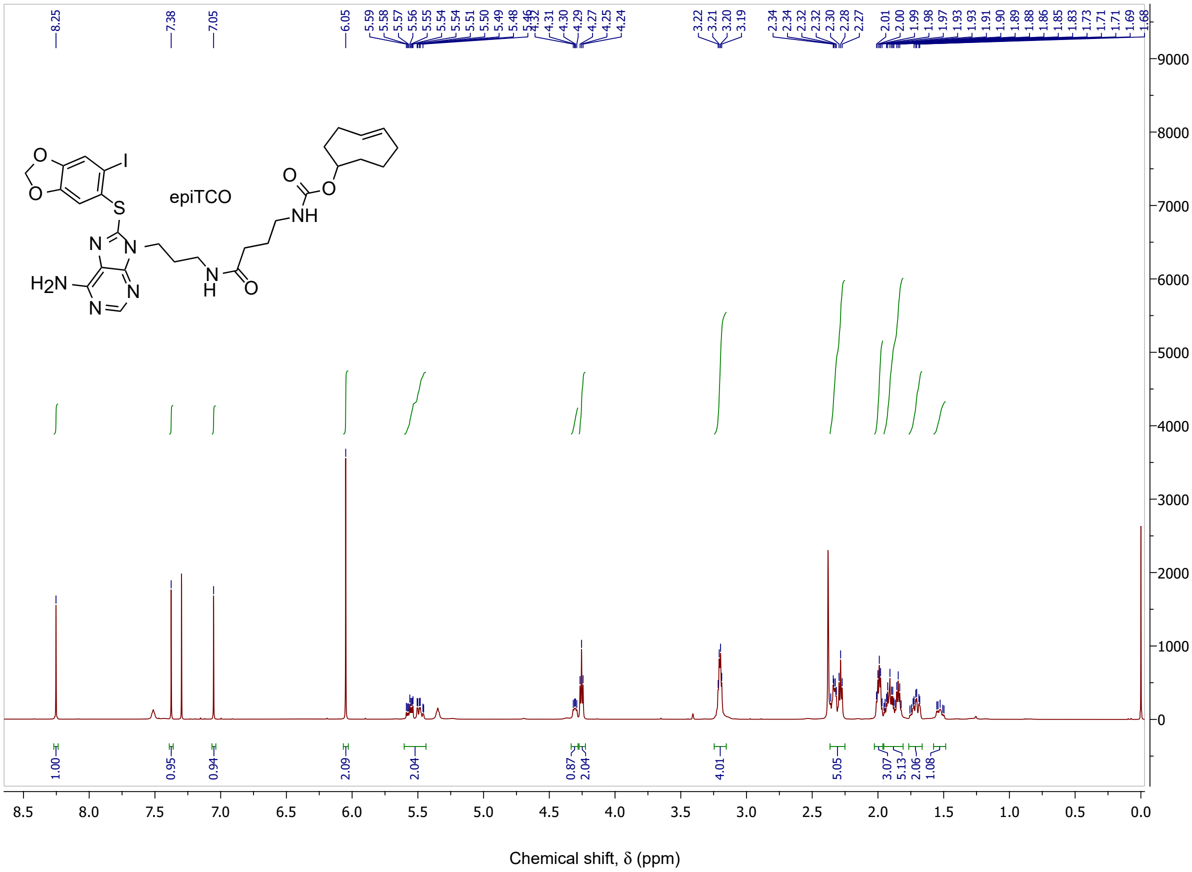

b

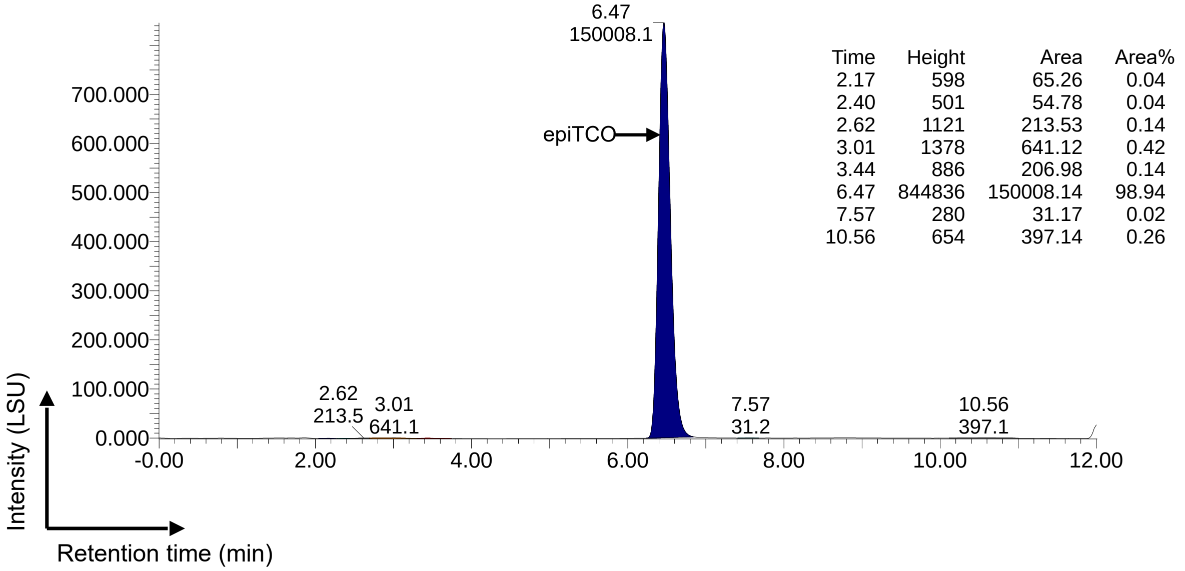

Supplementary Fig. 21. epiTCO characterization. a ^{1}H NMR spectra in $CDCl_3/CD_3OD$ and **b** HPLC trace (chromatogram, retention time and peak integration are shown). See Supplementary Note 1 for synthesis, purification and characterization and for ^{13}C NMR.

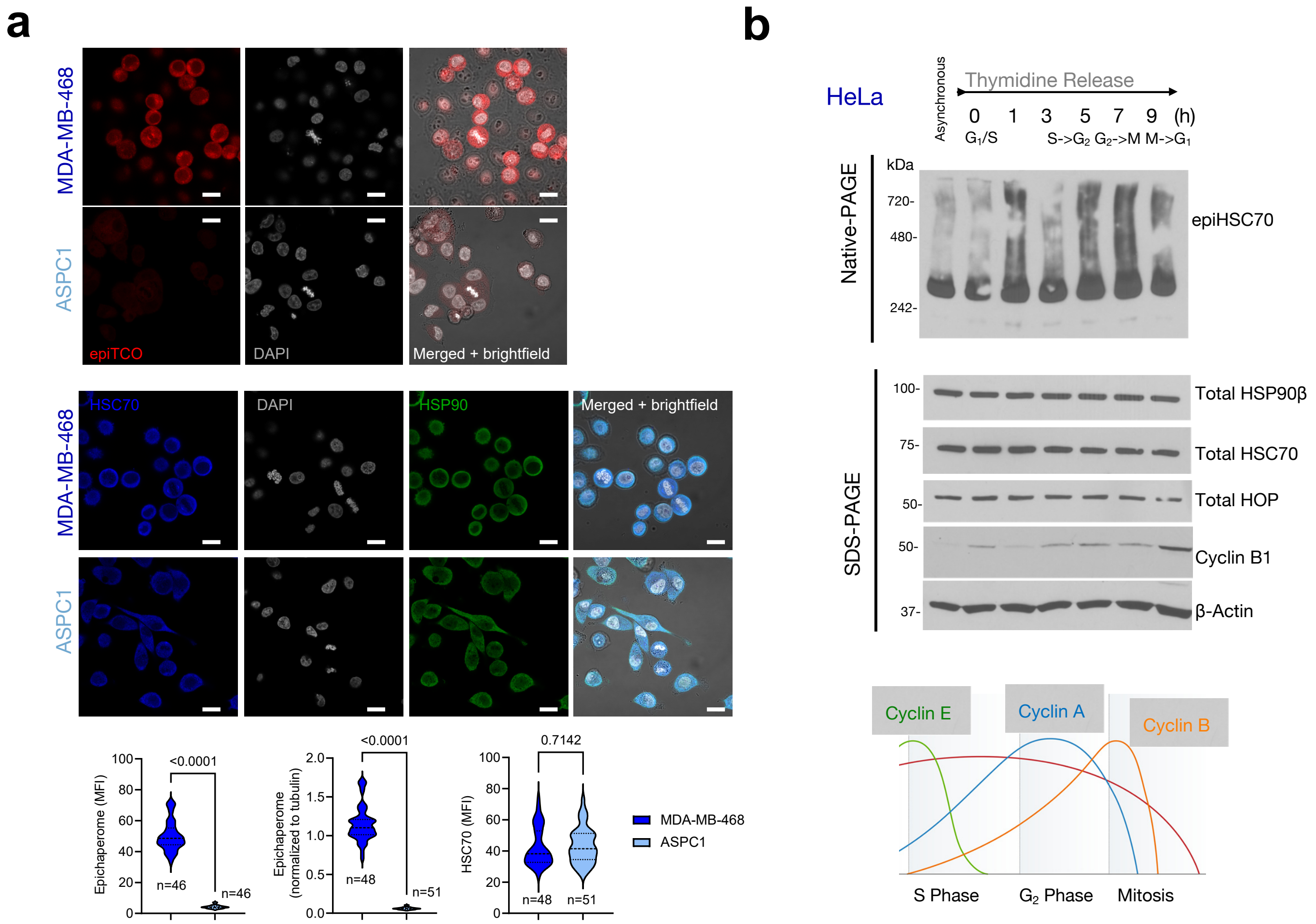

Supplementary Figure 22. EpiHSP70s levels monitoring.
a epiHSP70s and HSP70s levels in asynchronous MDA-MB-468 and ASPC1 cells visualized and quantified by confocal microscopy, as indicated. Graph, median, dotted line and quartiles, dashed lines, n, number of cells as indicated, unpaired two-tailed t-test. Top, representative micrographs. Scale bar, 10 μm. **b** Changes in epiHSP70s levels in the HeLa cancer cells released from double thymidine block were monitored by native PAGE and immunobloting with indicated antibodies. Total levels of chaperones and cyclins were analyzed by Western blot. Experiment was repeated three times with similar results. Source data, along with statistical analyses and statistics output, are provided as Source data files.

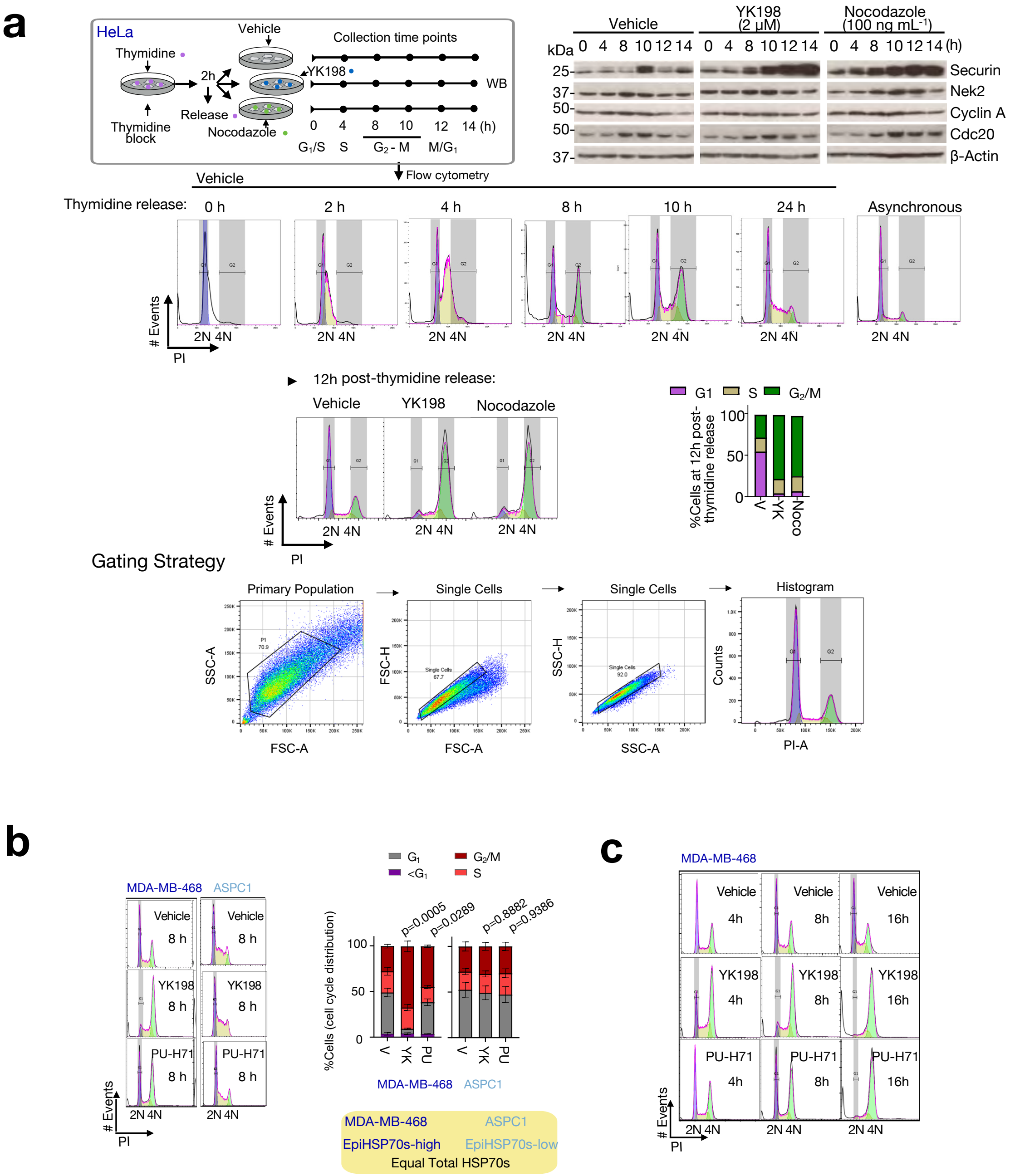

Supplementary Figure 23. EpiHSP70s inhibition leads to accumulation of cells in mitosis - flow cytometry analysis.

a Cell cycle analysis of HeLa epichaperome-positive cells released from double thymidine block into Vehicle, or YK198 (2 μM). Nocodazole, positive control, mitotic block. Representative western blot (mitotic markers) and flow cytometry (propidium iodide staining) analyses of 3 independent experiments are shown. **b c** Cell cycle analysis of MDA-MB-468 epichaperome-high cells treated with Vehicle or YK198 (2 μM). ASPC1, control for cell line with low/negative epichaperome levels but with high HSP70s levels. PU-H71 (2 μM), control for epichaperome inhibition. Data in (b) are presented as mean ± s.e.m., n = 3, one-way ANOVA with Dunnett's post-hoc, for the G_2/M population compared to Vehicle. Source data are provided as Source data files.

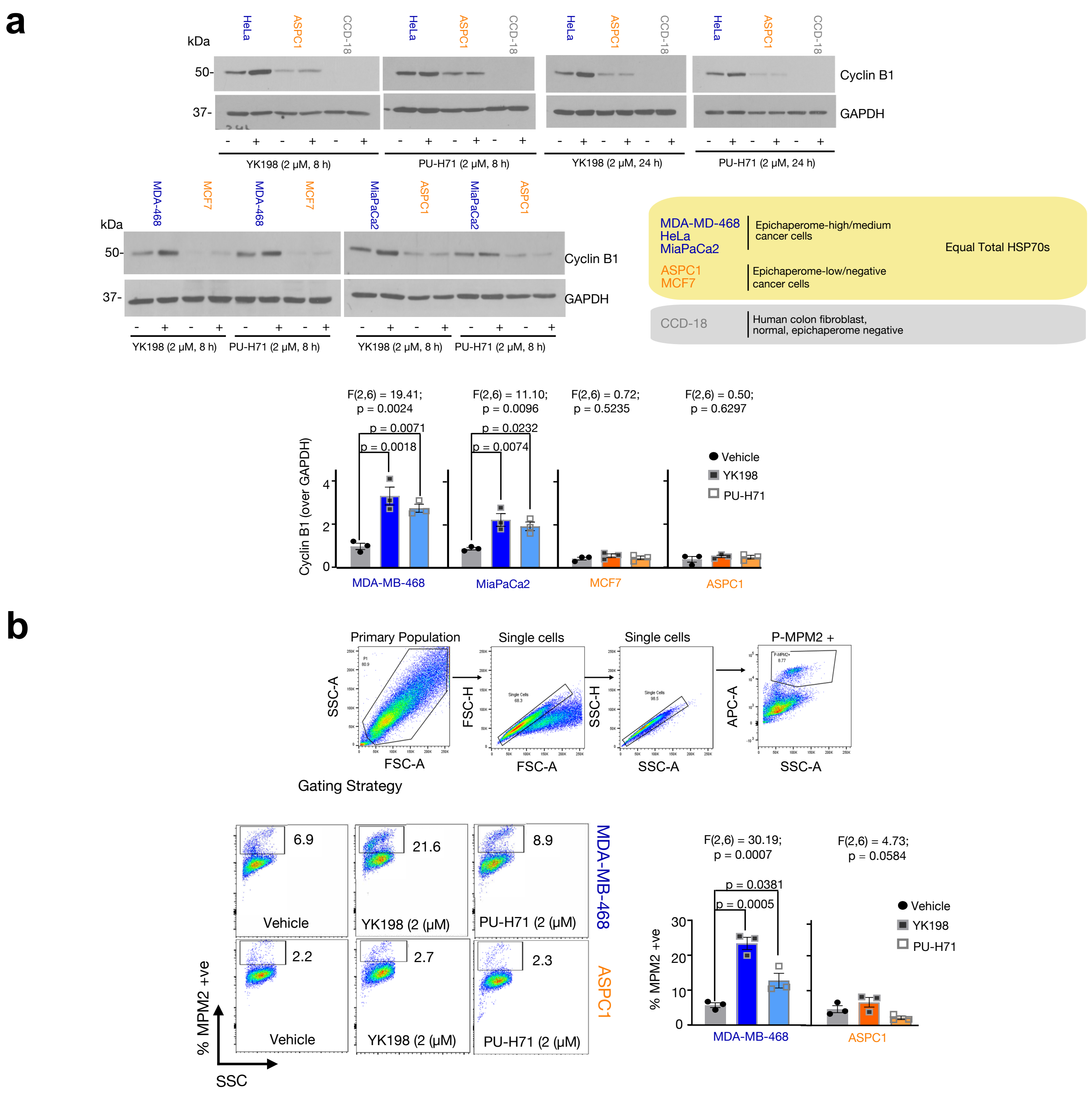

Supplementary Figure 24. EpiHSP70s inhibition leads to accumulation of cells in mitosis - evaluation of mitotic markers.
a Changes in cyclin B1 levels in asynchronous epiHSP70s-high/medium cells (MDA-MB-468, HeLa, MiaPaCa2) treated with Vehicle (-) or YK198 (2 μM) for 8 h or 24 h, as indicated, were monitored by Western blot. GAPDH, protein loading control. ASPC1, MCF7 and CCD18 control for cell lines with low/negative epiHSP70s levels. PU-H71 (2 μM), control for epichaperome inhibition. **b** Mitotic cells treated as in (a) identified by mitosis-specific phosphoepitopes of MPM-2 (mitotic protein monoclonal 2). Data in a,b are presented as mean ± s.e.m., n = 3, one-way ANOVA with Dunnett's post-hoc, compared to Vehicle. Source data are provided as Source data files.

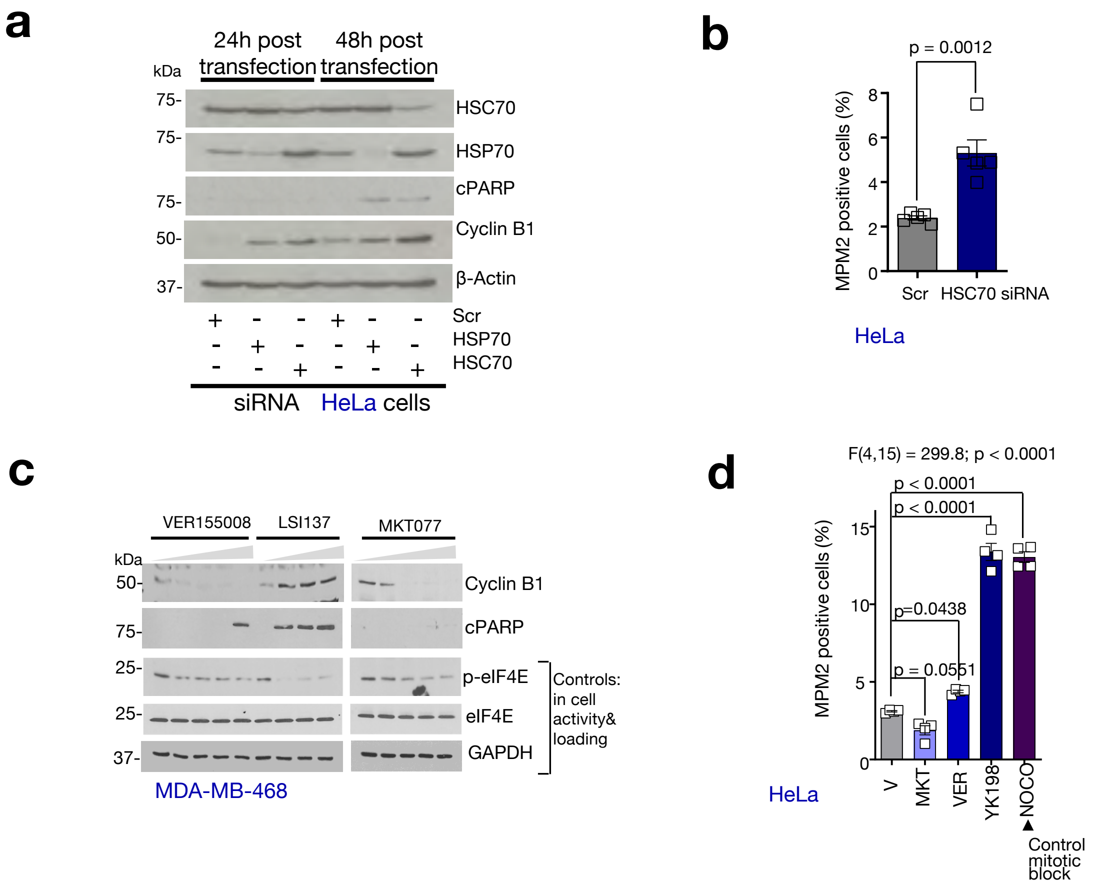

Supplementary Figure 25. Mitotic markers analyzed upon genetic or pharmacologic inhibition of various HSP70s pools.

a b Analysis of cyclin B1 levels (a) and MPM2-positive cells (b) in epiHSP70s+ve HeLa cells treated with scramble siRNA or siRNAs targeting epiHSP70s via knock-down of HSP70 or HSC70 (see Fig. 5d). **c** Western blot analysis of epiHSP70s+ve MDA-MB-468 cells treated for 24 h with VER155008 (0, 5, 10, 25 and 50 µM), LSI137 (0, 50, 100 and 200 nM) or MKT077 (0, 1, 2.5, 5, 10 µM). **d** MPM2-positive cells in HeLa cells treated for 8 h with the indicated inhibitors. V, vehicle; MKT077 (20 µM); VER155008 (20 µM); YK198 (2 µM); NOCO, nocodazole (0.3 µM). Experiments in (a,c) were repeated three times with similar results. Data in (b) are presented as mean ± s.e.m., n = 4, unpaired two-tailed t-test. Data in (d) are presented as mean ± s.e.m., n = 4, one-way ANOVA with Dunnett's post-hoc, compared to Vehicle. Source data are provided as Source data files.

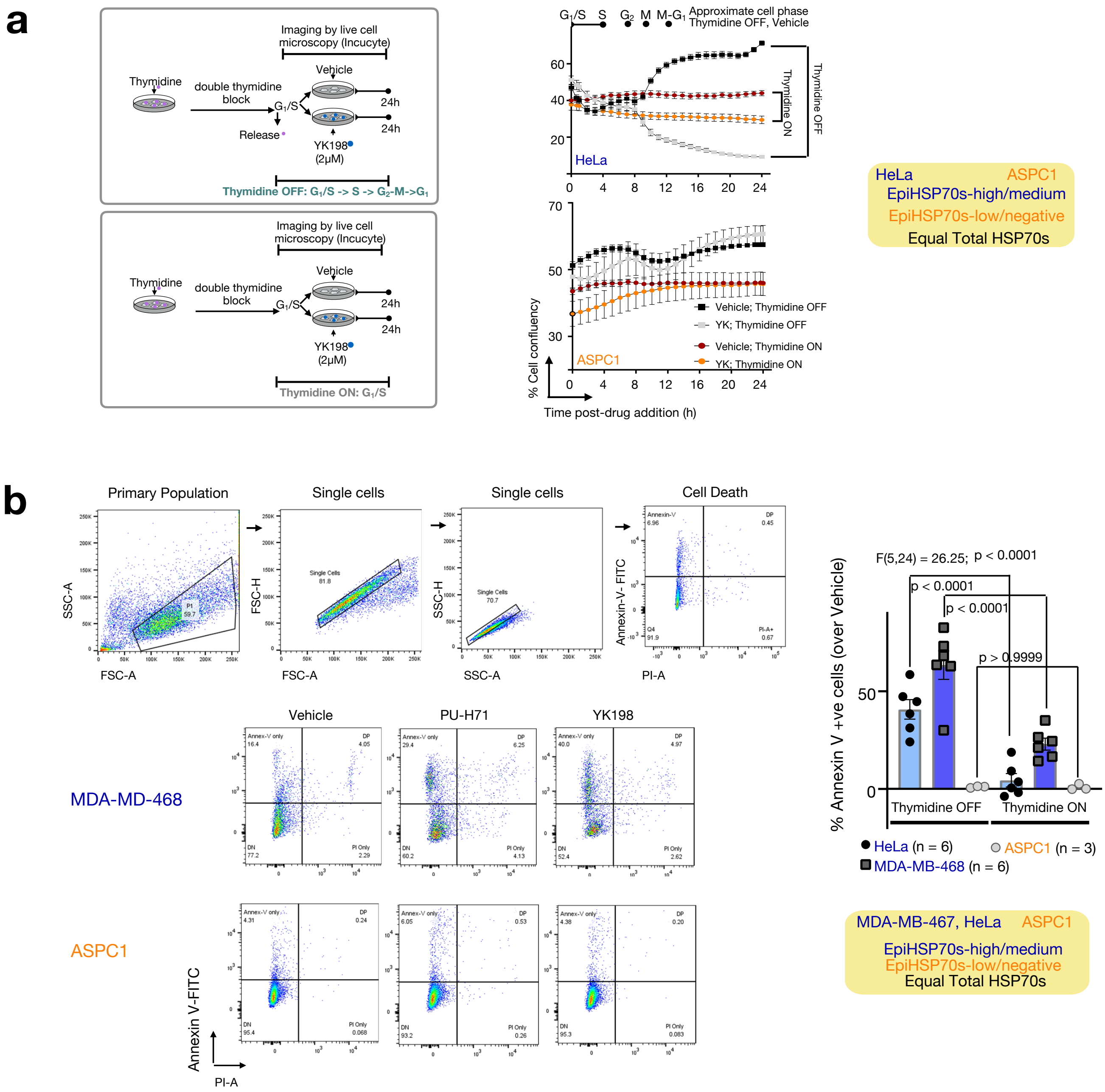

Supplementary Figure 26. Mitotic block upon epiHSP70s inhibition is lethal.
a Cell confluency monitored by live cell microscopy in epiHSP70s-high/medium and -low/negative cancer cells (with equivalent HSP70s levels) treated with vehicle (-) or YK198 (2 μM), after being released from or kept in thymidine, as indicated. Each curve represents the mean ± s.e.m. of 3 wells, and the data is representative of 3 independent experiments. **b** Annexin V staining of cells treated as in (a) for 24 h. Data are presented as mean ± s.e.m., one-way ANOVA with Sidak's post-hoc. Representative gating strategy and histograms of cells treated for 24 h are also shown. Source data are provided as Source data files.

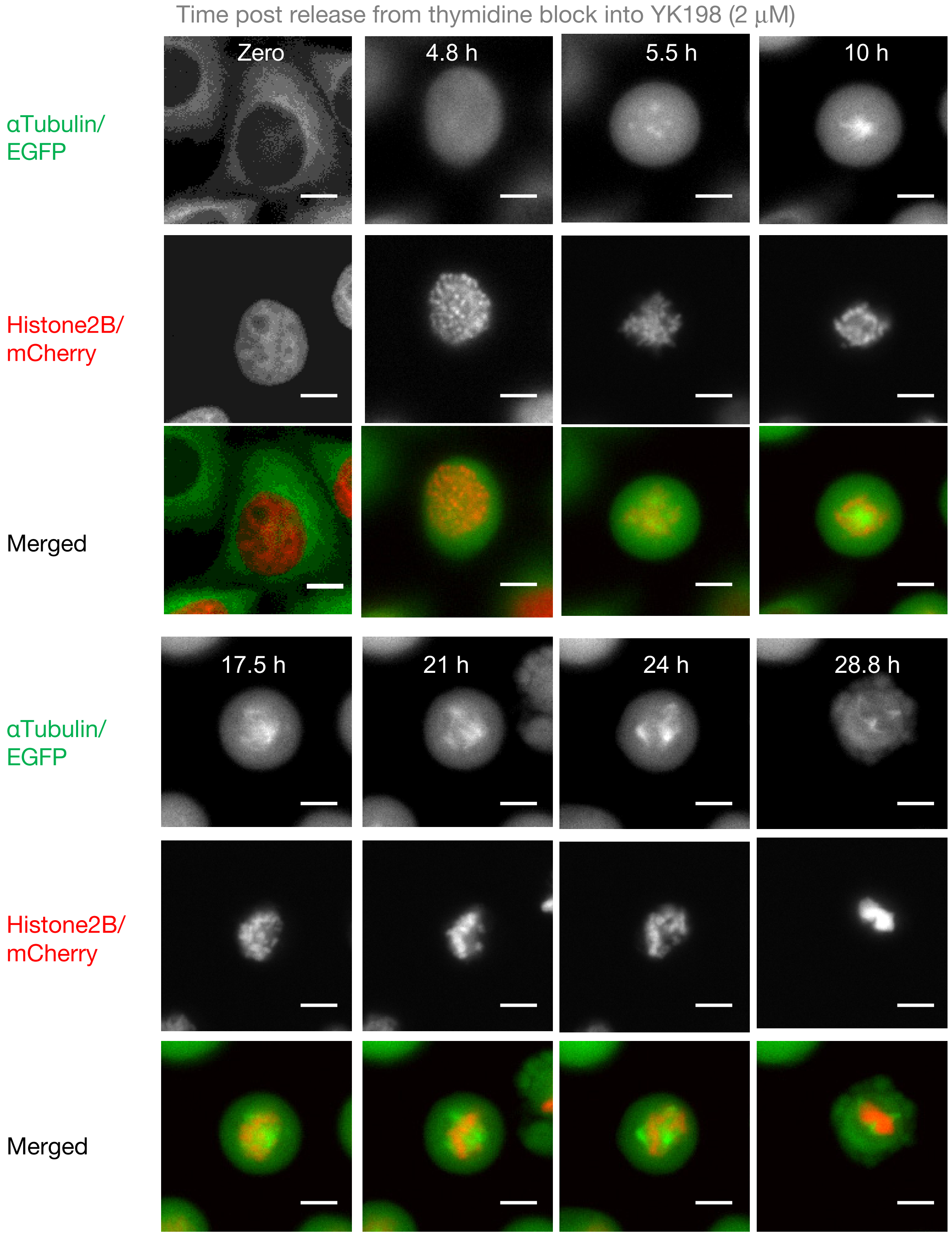

Supplementary Figure 27. Morphology of cells released from thymidine block into YK198. Representative time-lapse microscopy images of HeLa cells (epiHSP70s-positive cancer cells) released from thymidine block into YK198 (n = 103). Scale bar, 25 µm. Related to Fig. 8g.

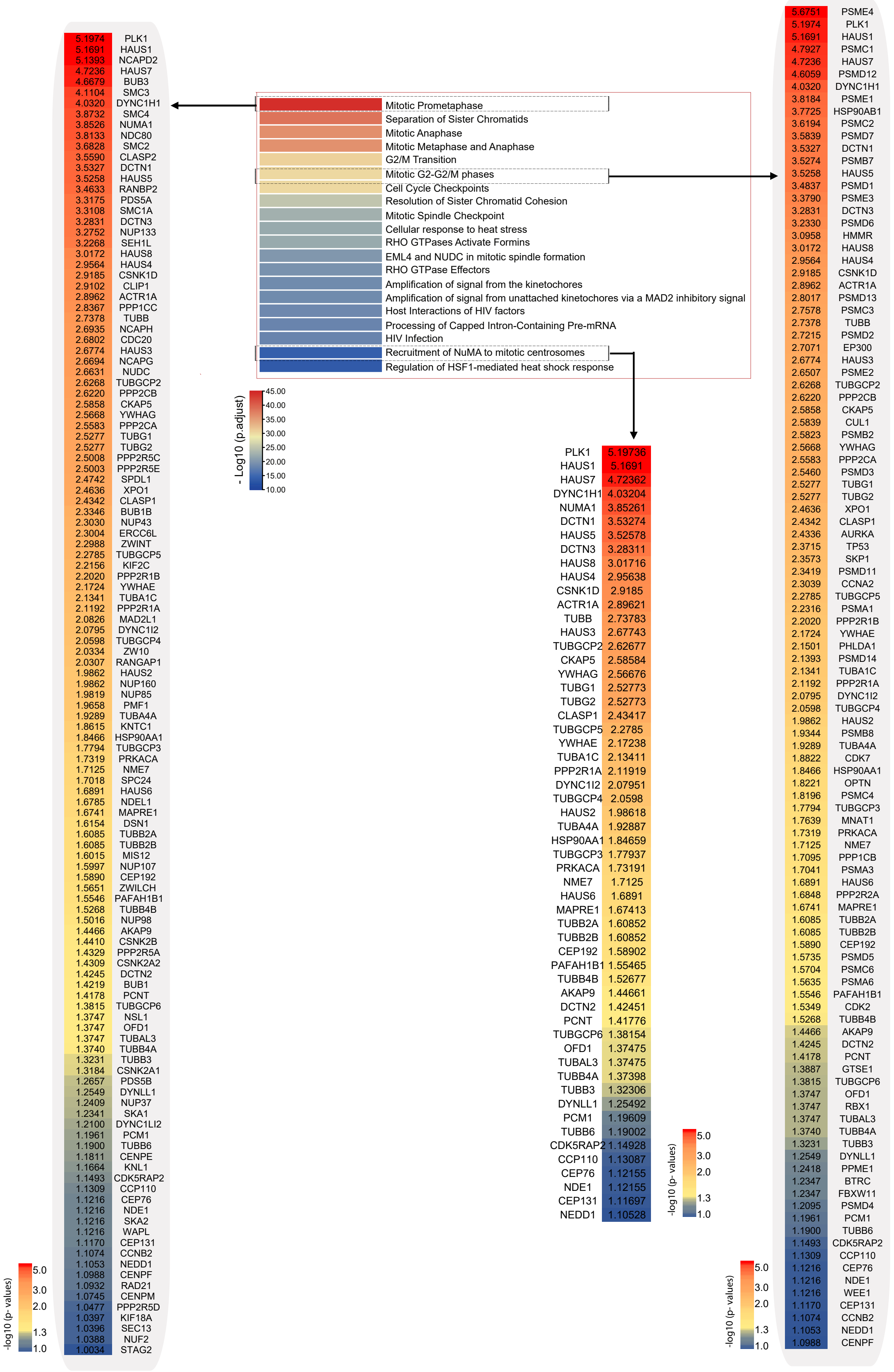

Supplementary Fig. 28. Functional annotation of Grade A proteins via Reactome pathway analysis. Top 20 pathways ranked by the p.adjust values and interactors, as identified by the YK5-B bait, that were mapped to the indicated pathways, are shown. Proteins are graphed based on their interactor grade value (i.e. p-values calculated as in Fig. 5d based on a two-sided t-test). Size bar (blue, yellow, red), negative log10 (p-value). Source data are provided as Supplementary Data files.

97 Assays Tested
2 Interactions Mapped S-score(35) = 0.022

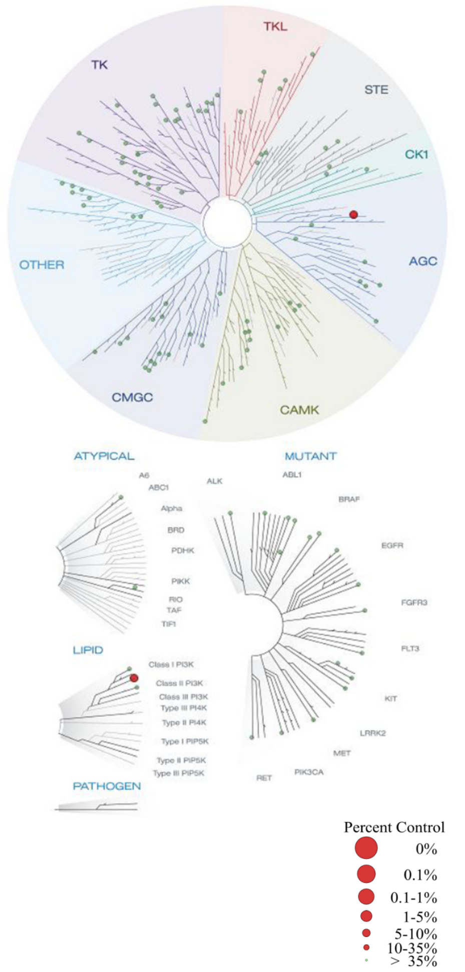

KINOMEscan Gene Symbol		
ABL1(E255K)-phosphorylated	*FAK*	*PDGFRA*
ABL1(T315I)-phosphorylated	*FGFR2*	*PDGFRB*
ABL1-nonphosphorylated	*FGFR3*	*PDPK1*
ABL1-phosphorylated	*FLT3*	*PIK3C2B*
ACVR1B	*GSK3B*	*PIK3CA*
ADCK3	*IGF1R*	*PIK3CG*
AKT1	*IKK-alpha*	*PIM1*
AKT2	*IKK-beta*	*PIM2*
ALK	*INSR*	*PIM3*
AURKA	*JAK2(JH1domain-catalytic)*	*PKAC-alpha*
AURKB	*JAK3(JH1domain-catalytic)*	*PLK1*
AXL	*JNK1*	*PLK3*
BMPR2	*JNK2*	*PLK4*
BRAF	*JNK3*	*PRKCE*
BRAF(V600E)	*KIT*	*RAF1*
BTK	*KIT(D816V)*	*RET*
CDK1	*KIT(V559D,T670I)*	*RIOK2*
CDK2	*LKB1*	*ROCK2*
CDK3	*MAP3K4*	*RSK2(Kin.Dom.1-N-terminal)*
CDK7	*MAPKAPK2*	*SNARK*
CDK9	*MARK3*	*SRC*
CHEK1	*MEK1*	*SRPK3*
CSF1R	*MEK2*	*TGFBR1*
CSNK1D	*MET*	*TIE2*
CSNK1G2	*MKNK1*	*TRKA*
DCAMKL1	*MKNK2*	*TSSK1B*
DYRK1B	*MLK1*	*TYK2(JH1domain-catalytic)*
EGFR	*p38-alpha*	*ULK2*
EGFR(L858R)	*p38-beta*	*VEGFR2*
EPHA2	*PAK1*	*YANK3*
ERBB2	*PAK2*	*ZAP70*
ERBB4	*PAK4*	
ERK1	*PCTK1*	

Supplementary Figure 29. scanEDGE kinase screen.
YK198 (at 10 µM) was tested in the scanEDGE screen against 97 kinases. Tree map and the list of kinases encompassing the screen, are presented. KINOMEscan's selectivity score (S) is a quantitative measure of compound selectivity. It is calculated by dividing the number of kinases that bind to the compound by the total number of distinct kinases tested, excluding mutant variants. The higher the S values, the less selective the compound. S(35) = (number of nonmutant kinases with %Ctrl < 35)/(number of nonmutant kinases tested).

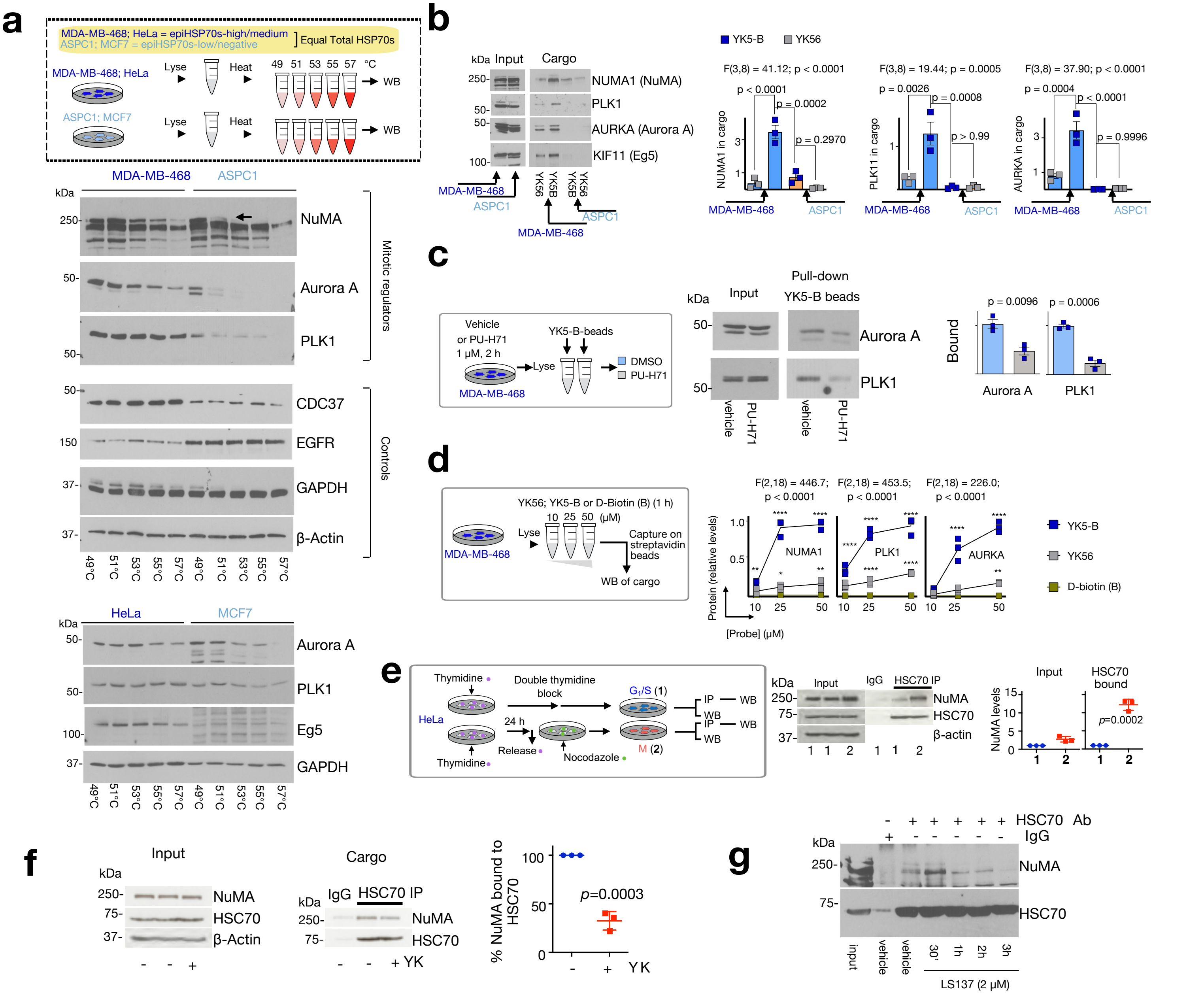

Supplementary Figure 30. Context-dependent stability and epiHSP70s-dependence of mitotic regulators. a CETSA: Melting curves for indicated mitotic proteins. Experiments were repeated three times with similar results. **b** Mitotic proteins captured by the YK5-B probe and the control probe YK56, as indicated. Data are presented as mean ± s.e.m., n = 3, one-way ANOVA with Tukey's post-hoc. **c** Affinity purification performed with YK5-B in MDA-MB-468 cells pre-treated with PU-H71 or Vehicle, as indicated. Data are presented as mean ± s.e.m., n = 3, unpaired two-tailed t-test. **d** Mitotic proteins captured by the YK5-B probe and the control probe YK56, as indicated. Data are presented as mean ± s.e.m., n = 3, two-way ANOVA with Dunnett's post-hoc, compared to D-biotin. **e** Interaction of mitotic proteins (i.e. NuMA) with epiHSP70s analyzed by immunoprecipitation (IP) and western blot (WB). Graphs are mean ± s.e.m. of 3 repeats; unpaired two-tailed t-test. IgG, isogenic control. **f** As in (d) for cells pretreated with YK5 (10 μM) or Vehicle. **g** Cells were treated with LSI or vehicle for the indicated time, prior to lyzing and antibody capture, to show temporary trapping of the epiHSP70s-interactor prior to assembly disruption. Gel images representative of three (n = 3) independent experiments. Source data are provided as Source data files.

Supplementary Note 1: Chemical synthesis and compound characterization

General methods: All commercial chemicals and solvents were reagent grade and used without further purification. The identity and purity of each product was characterized by MS, HPLC, TLC, and NMR. $^1H/^{13}C$ NMR spectra were recorded on either a Bruker 500 or 600 MHz instrument. Chemical shifts are reported in δ values in ppm downfield from TMS as the internal standard. ^1H data are reported as follows: chemical shift, multiplicity (s = singlet, d = doublet, t = triplet, q = quartet, br = broad, m = multiplet), coupling constant (Hz), integration. ^{13}C chemical shifts are reported in δ values in ppm downfield from TMS as the internal standard. Low resolution mass spectra were obtained on Waters Acquity Ultra Performance LC with electrospray ionization and SQ detector. Purity of target compounds has been determined to be >95% by LC/MS on a Waters Autopurification system with PDA, MicroMass ZQ and ELSD detector and a reversed phase column (Waters X-Bridge C18, 4.6 x 150 mm, 5 μm) eluted with water/acetonitrile gradients, containing 0.1% TFA. Column chromatography was performed using 230-400 mesh silica gel. Analytical thin layer chromatography was performed on 250 μM silica gel F254 plates. Preparative thin layer chromatography was performed on 1000 μM silica gel F254 plates. Flash chromatography was performed using CombiFlash®R_f instrument.

Synthesis of YK198

4-(Benzyloxy)-2-chloropyrimidine (2) and 4-(benzyloxy)-2-(4-methylpiperazin-1-yl) pyrimidine (3): To a solution of 2,4- dichloropyrimidine (**1**) (2.0 g, 0.0134 mmol) in toluene (20 mL) were added benzyl alcohol (1.53 mL, 1.59 g, 0.0147 mol), KOH (0.82 g, 0.0147 mol), and 18-crown-6 (0.177 g, 0.00067 mol), and the resulting solution was stirred at rt for 1 h. The reaction mixture was diluted with EtOAc (400 mL), washed with water (3 × 50 mL), dried over $MgSO_4$, filtered, and concentrated to give a white solid that was chromatographed (hexane/CH_2Cl_2, 1:1 to 3:7) to afford 2.09 g (71%) of a regioisomeric mixture with desired regioisomer 2-(benzyloxy)-4-chloropyrimidine (**2**) [**MS (*m/z*):** $[M + Na]^+$ 243.1]. Next, to a solution of **2** (2.09 g, 0.00947 mol; containing regioisomers) in DMF (34 mL) was added 1-methylpiperazine (3.15 mL, 2.85 g, 0.0284 mol), and the resulting solution was heated at 80 °C for 1.75 h. Solvent was removed under reduced pressure, and the residue was taken up into EtOAc (350 mL) and washed with brine (3 × 50 mL). The aqueous layer was extracted with EtOAc (2 × 50 mL), and the combined organic layers were dried over $MgSO_4$, filtered, and concentrated to give an oil that was purified by column chromatography (EtOAc/MeOH−NH_3 (7 N), 1:0 to 25:1) to afford 1.88 g (70%) of **3**. **^{1}H NMR (500 MHz, $CDCl_3$):** δ 8.06 (d, *J* = 5.6 Hz, 1H), 7.41 (d, *J* = 7.0 Hz, 2H), 7.35 (t, *J* = 7.0 Hz, 2H), 7.32 (d, *J* = 7.0 Hz, 1H), 6.03 (d, *J* = 5.6 Hz, 1H), 5.35 (s, 2H), 3.83 (m, 4H), 2.45 (m, 4H), 2.33 (s, 3H); **^{13}C NMR (125 MHz, $CDCl_3$)**: δ 169.3, 161.6, 158.1, 136.8, 128.5, 128.0, 96.9, 67.3, 54.9, 46.2, 43.7; **MS (*m/z*):** $[M + H]^+$ 284.91.

4-(Benzyloxy)-5-iodo-2-(4-methylpiperazin-1-yl)pyrimidine (4): To a solution of **3** (0.937 g, 0.0033 mol) in acetonitrile (16 mL) were added TFA (1.02 mL, 1.51 g, 0.0132 mol) and *N*-iodosuccinimide (0.965 g, 0.0043 mol), and the resulting solution was stirred at rt for 1 h. Then 7 mL of 10% Na_2CO_3 (0.70 g, 0.066 mol) was added, and the resulting solution was stirred for 2 min. The reaction mixture was concentrated to dryness, and the residue was taken up into CH_2Cl_2 (200 mL) and washed with 10% Na_2CO_3 (2 × 50 mL), 10% sodium thiosulfate (50 mL), and brine (50 mL). The organic layer was dried over $MgSO_4$, filtered, and concentrated to give an oil which was purified by column chromatography (CH_2Cl_2/MeOH−NH_3 (7 N), 50:1) to yield 1.31 g (97%) of **4**. **^{1}H NMR (500 MHz, $CDCl_3$):** δ 8.27 (s, 1H), 7.44 (d, *J* = 7.4 Hz, 2H), 7.37 (t, *J* = 7.2 Hz, 2H), 7.32 (d, *J* = 7.3 Hz, 1H), 5.40 (s, 2H), 3.79 (m, 4H), 2.42 (m, 4H), 2.32 (s, 3H); **^{13}C NMR (150 MHz, $CDCl_3$)** δ 166.7, 164.2, 159.9, 135.9, 128.3, 127.8, 126.9, 68.58, 64.2, 53.02, 43.36, 40.94; **MS (m/z):** $[M + H]^+$ 411.02.

4-(Benzyloxy)-2-(4-methylpiperazin-1-yl)-5-((6-nitropyridin-3-yl)thio)pyrimidine (5): A mixture of **4** (0.500 g, 1.218 mmol) and K_2CO_3 (0.505 g, 3.656 mmol) in DMF (10 mL) was evacuated and backfilled with argon three times. Copper(I)thiophene-2-carboxylate (0.093 g, 0.487 mmol) was added and evacuated and backfilled with argon two times. 6-Nitropyridine-3-thiol (0.380 g, 2.436 mmol) was added and the reaction mixture was heated at 130 °C for 6 h. Solvent was removed under reduced pressure and the residue was purified by column chromatography (CH_2Cl_2:MeOH, 0-10% MeOH) to afford 0.395 g (74%) of **5**. **^{1}H NMR (500 MHz, CDCl$_3$):** δ 8.33 – 8.25 (m, 2H), 7.99 – 7.93 (m, 1H), 7.49 – 7.43 (m, 1H), 7.27 (s, 3H), 7.17 (s, 2H), 5.36 (s, 2H), 3.91 (s, 4H), 2.50 (s, 4H), 2.37 (s, 3H); **^{13}C NMR (125 MHz, CDCl$_3$):** δ 168.3, 164.9, 161.8, 154.1, 145.3, 144.1, 135.9, 135.6, 128.5, 128.2, 127.7, 117.8, 95.2, 68.1, 54.7, 46.1, 43.9; **MS (m/z):** $[M + H]^+$ 438.92.

5-((4-(Benzyloxy)-2-(4-methylpiperazin-1-yl)pyrimidin-5-yl)thio)pyridin-2-amine (6, YK198): To a mixture of **5** (0.100 g, 0.228 mmol) in acetic acid (5 mL), Fe (0.064 g, 1.14 mmol) was added. The reaction was stirred at room temperature for 3 h. The mixture was then diluted with CH_2Cl_2, filtered through celite pad, and the cake was washed with CH_2Cl_2. The volatile solvents were removed in vacuo and the water (10 mL) was added. The water phase was then neutralized to pH 7-8 with sodium bicarbonate. The resulting mixture was extracted with CH_2Cl_2 (20 mL) for three times. The organic extracts were combined, washed with brine, dried over anhydrous sodium sulfate, concentrated and was purified by preparatory TLC (CH_2Cl_2:MeOH (7N), 20:1) to afford 78.1 mg (80%) of **6**. **^{1}H NMR (500 MHz, CDCl$_3$):** δ 8.20 (s, 1H), 8.12 (s, 1H), 7.41 (dd, *J* = 8.5, 2.3 Hz, 1H), 7.35 – 7.26 (m, 5H), 6.34 (d, *J* = 8.5 Hz, 1H), 5.36 (s, 2H), 4.50 (br s, 2H), 3.83 (s, 4H), 2.47 (s, 4H), 2.36 (s, 3H); **^{13}C NMR (150 MHz, CDCl$_3$):** δ 167.8, 162.4, 161.0, 157.5, 150.7, 141.9, 136.5, 128.4, 127.9, 127.7, 120.5, 108.9, 103.2, 67.8, 54.6, 46.0, 43.6; **MS (m/z):** $[M + H]^+$ 409.07; **HPLC**: (a) H_2O + 0.1% TFA, (b) ACN + 0.1% TFA (5−95% ACN in 12 min at 1.2 mL/min) R_t = 4.47 min, 99.42%.

<u>**Synthesis of LSI137**</u>

4-((3-Methoxybenzyl)oxy)-2-(methylthio)pyrimidine (8) : To a solution of 4-methoxybenzyl alcohol (5.2 g, 37.355 mmol) in 50 mL acetonitrile at 0°C, NaH (1.12 g, 46.693 mmol) was added portion wise and the mixture stirred at 0°C for 30 min. 4-chloro-2-(methylthio)pyrimidine (**7**) (5 g, 31.129 mmol) was added slowly and the mixture was allowed stir at rt overnight. The reaction was quenched with methanol and the solvent was removed under reduced pressure. The residue was dissolved in EtOAc, washed with brine, and dried over Na_2SO_4. Solvent was evaporated under reduced pressure and the residue was purified by column chromatography (Hexane:EtOAc, 4:1) to afford 7.5 g (92 %) of **8**. **^{1}H NMR (500 MHz, CDCl$_3$)**: δ 8.24 (d, *J* = 5.7 Hz, 1H), 7.34 – 7.21 (m, 1H), 7.04 – 6.97 (m, 2H), 6.91 – 6.81 (m, 1H), 6.44 (d, *J* = 5.7 Hz, 1H), 5.39 (s, 2H), 3.82 (s, 3H), 2.55 (s, 3H); **^{13}C NMR (125 MHz, CDCl$_3$)**: δ 172.1, 168.3, 159.7, 157.3, 137.6, 129.6, 120.4, 113.7, 113.7, 103.8, 67.9, 55.2, 14.1; **MS (*m/z*):** $[M+H]^+$ 263.29.

4-((3-Methoxybenzyl)oxy)-2-(methylsulfonyl)pyrimidine (9): To a stirred solution of 4-((3-methoxybenzyl)oxy)-2-(methylthio)pyrimidine (**8**) (5 g, 19.061 mmol) in dichloromethane (100 ml) at 0 °C was added *meta*-chloroperoxybenzoic acid (8.22 g, 47.652 mmol) over a period of 30 minutes. The solution was allowed to warm to room temperature and was stirred for 8 hours. The mixture was diluted with dichloromethane (100 mL) and then treated with sat. $NaHCO_3$ solution (3 x 100 ml), and brine (200 ml) then dried over Na_2SO_4. Solvent was evaporated under reduced pressure to provide crude product which was purified by column chromatography (Hexane:EtOAc, 1:1) to afford 3.5 g (63 %) of **9**. **^{1}H NMR (500 MHz, CDCl$_3$)**: δ 8.56 (d, *J* = 5.8 Hz, 1H), 7.30 (t, *J* = 7.8 Hz, 1H), 7.06 – 7.04 (m, 2H), 6.95 (d, *J* = 5.8 Hz, 1H), 6.90 (dd, *J* = 8.3, 1.8 Hz, 1H), 5.50

(s, 2H), 3.82 (s, 3H), 3.31 (s, 3H); **^{13}C NMR (125 MHz, $CDCl_3$)**: δ 170.1, 165.2, 159.8, 157.9, 136.3, 129.8, 120.8, 114.4, 114.1, 111.7, 69.7, 55.3, 39.1; **MS (*m/z*)**: $[M+H]^+$ 295.09.

(*R*)-1-(4-((3-methoxybenzyl)oxy)pyrimidin-2-yl)-*N,N*-dimethylpyrrolidin-3-amine (10) : To a stirred solution of **9** (3 g, 10.193 mmol) in DMF (25 ml) was added (*R*)-*N*,*N*-dimethylpyrrolidin-3-amine (1.4 g, 12.232 mmol) at rt. The solution was then heated at 80 ºC for 6 hours. After completion of the reaction, the mixture was concentrated under reduced pressure and the residue was purified by column chromatography (CH_2Cl_2:MeOH, 20:1) to afford 2.7 g (80 %) of **10**. **^{1}H NMR (500 MHz, $CDCl_3$)**: δ 8.07 (d, *J* = 5.7 Hz, 1H), 7.30 –7.25 (m, 1H), 7.03 – 6.96 (m, 2H), 6.85 (dd, *J* = 8.2, 1.9 Hz, 1H), 6.02 (d, *J* = 5.7 Hz, 1H), 5.34 (s, 2H), 3.94 – 3.87 (m, 1H), 3.85 – 3.77 (m, 4H), 3.48 (td, *J* = 10.8, 6.9 Hz, 1H), 3.33 – 3.25 (m, 1H), 2.83 – 2.73 (m, 1H), 2.32 (s, 6H), 2.19 (dt, *J* = 12.0, 6.4 Hz, 1H), 1.94 – 1.79 (m, 1H); **^{13}C NMR (125 MHz, $CDCl_3$)**: δ 169.1, 160.1, 159.7, 158.1, 138.6, 129.5, 120.3, 113.6, 113.4, 96.2, 67.1, 65.5, 55.2, 50.9, 45.7, 44.4, 30.3; **MS (*m/z*)**: $[M+H]^+$ 328.99.

(*R*)-1-(5-iodo-4-((3-methoxybenzyl)oxy)pyrimidin-2-yl)-*N,N*-dimethylpyrrolidin-3-amine (11): To a mixture of **10** (2 g, 6.089 mmol) in acetonitrile (50 mL) was added *N*-iodosuccinimide (1.64 g, 7.307 mmol) and trifluoroacetic acid (470 µL, 2.435 mmol). The mixture was stirred at rt for 3 h and then the reaction was quenched with 5 percent aqueous sodium thiosulphate solution (100 mL) and the solvent removed in vacuo. The product was then extracted into dicholoromethane (3 x 50 mL) and the combined organics were washed with brine (50 mL) and dried over $MgSO_4$. Solvent was evaporated under reduced pressure and the residue was purified by column chromatography (CH_2Cl_2:MeOH, 20:1) to afford 2.2 g (78 %) of **11**. **^{1}H NMR (500 MHz, $CDCl_3/CD_3OD$)**: δ 8.28 (s, 1H), 7.30 (t, *J* = 8.1 Hz, 1H), 7.05 – 7.01 (m, 2H), 6.89 – 6.85 (m, 1H), 5.43 (s, 2H), 4.07 – 3.94 (m, 2H), 3.88 (t, *J* = 8.9 Hz, 1H), 3.84 – 3.76 (m, 4H), 3.61 – 3.53 (m, 1H), 2.93 (s, 6H), 2.58 – 2.50 (m, 1H), 2.42 (dq, *J* = 12.7, 8.7 Hz, 1H); **^{13}C NMR (125 MHz, $CDCl_3/CD_3OD$)**: δ 167.1, 164.3, 160.1, 159.3, 138.3, 130.0, 119.9, 113.6, 113.3, 78.0, 68.8, 65.2, 63.5, 55.5, 45.5, 42.1, 27.5; **MS (*m/z*)**: $[M+H]^+$ 455.27

(*R*)-1-(4-((3-methoxybenzyl)oxy)-5-((6-nitropyridin-3-yl)thio)pyrimidin-2-yl)-*N,N*-dimethyl pyrrolidin-3-amine (**12**)**:** A mixture of **11** (0.500 g, 1.101 mmol) and K_2CO_3 (152 mg, 3.301 mmol) in DMF (10 mL) was evacuated and backfilled with argon. Copper(I)thiophene-2-carboxylate (83.98 mg, 0.440 mmol) was added and evacuated and backfilled with argon. 4-Mercaptobenzonitrile (361 mg, 2.312 mmol) was added and the reaction mixture was heated at 120 °C for 3 h. Solvent was removed under reduced pressure and the residue was purified by column chromatography (CH_2Cl_2:MeOH-NH_3 (7N), 20:1) to afford 383 mg (72%) of **12**. **^{1}H NMR (500 MHz, $CDCl_3$)**: δ 8.31 (s, 1H), 8.28 (s, 1H), 7.97 (d, *J* = 8.2 Hz, 1H), 7.43 (d, *J* = 7.7 Hz, 1H), 7.17 (t, *J* = 7.5 Hz, 1H), 6.78 (s, 2H), 6.63 (s, 1H), 5.40 – 5.30 (m, 2H), 3.99 – 3.80 (m, 2H), 3.71 (s, 3H), 3.53 (dd, *J* = 17.8, 10.3 Hz, 1H), 3.36 (t, *J* = 8.4 Hz, 1H), 2.83 (d, *J* = 6.0 Hz, 1H), 2.34 (s, 6H), 2.24 (s, 1H), 2.01 – 1.88 (m, 1H); **^{13}C NMR (150 MHz, $CDCl_3$, *T* = 50 ºC)**: δ 168.3, 164.9, 160.7, 159.8, 154.3, 145.4, 144.2, 137.7, 135.7, 129.5, 120.0, 117.9, 114.0, 113.1, 95.2, 67.9, 65.4, 55.2, 51.2, 46.2, 44.2, 30.2, 30.0; **MS (*m/z*)**: $[M+H]^+$ 483.21.

(*R*)-5-((2-(3-(dimethylamino)pyrrolidin-1-yl)-4-((3-methoxybenzyl)oxy)pyrimidin-5-yl)thio) pyridin-2-amine (**13**, **LSI137**)**:** To a mixture of **12** (0.100 g, 0.207 mmol) in acetic acid (5 mL), Fe (0.058 g, 1.036 mmol) was added. The reaction was stirred at room temperature for 3 h. The mixture was then diluted with CH_2Cl_2, filtered through celite pad, and the cake was washed with CH_2Cl_2. The volatile solvents were removed in vacuo and the water (10 mL) was added. The water phase was then neutralized to pH 7-8 with sodium bicarbonate. The resulting mixture was extracted with CH_2Cl_2 (15 mL) for three times. The organic extracts were combined, washed with brine, dried over anhydrous sodium sulfate, concentrated and was purified by preparatory TLC (CH_2Cl_2:MeOH (7N), 20:1) to afford 44 mg (47%) of **13** (LSI137). **^{1}H NMR (500 MHz, $CDCl_3$)**: δ 8.23 (s, 1H), 8.13 (s, 1H), 7.39 (dd, *J* = 8.5, 1.6 Hz, 1H), 7.23 (t, *J* = 7.8 Hz, 1H), 6.91 – 6.86 (m,

2H), 6.82 (d, *J* = 8.2 Hz, 1H), 6.33 (d, *J* = 8.5 Hz, 1H), 5.37 (s, 2H), 4.47 (s, 2H), 3.89 – 3.74 (m, 5H), 3.50 – 3.40 (m, 1H), 3.26 (t, *J* = 8.6 Hz, 1H), 2.79 – 2.70 (m, 1H), 2.30 (s, 6H), 2.22 – 2.13 (m, 1H), 1.92 – 1.81 (m, 1H); **^{13}C NMR (125 MHz, $CDCl_3$)**: δ 167.8, 162.7, 159.7, 159.6, 157.5, 150.9, 141.3, 138.3, 129.4, 121.0, 120.0, 113.4, 113.1, 108.7, 102.3, 67.6, 65.4, 55.2, 51.1, 45.9, 44.4, 30.3; **MS (*m/z*)**: $[M + H]^+$ 453.25; **HPLC**: (a) H_2O + 0.1% TFA, (b) ACN + 0.1% TFA (5−95% ACN in 12 min at 1.2 mL/min) R_t = 4.44 min, 99.15%.

***N*-(6-amino-2-((4,6-dimethoxy-2-(4-methylpiperazin-1-yl)pyrimidin-5-yl)thio)pyrimidin-4-yl) acrylamide (YK5, 14). YK5** (**14**) was synthesized as reported previously[1, 2]. **^{1}H NMR (600 MHz, $CDCl_3/CD_3OD$):** δ 7.02 (s, 1H), 6.38 (dd, *J* = 17.0, 1.0 Hz, 1H), 6.18 (dd, *J* = 16.9, 10.3 Hz, 1H), 5.75 (dd, *J* = 10.4, 0.9 Hz, 1H), 3.88 (s, 10H), 2.52 (br s, 4H), 2.38 (s, 3H); **^{13}C NMR (150 MHz, $CDCl_3/CD_3OD$):** δ 171.2, 170.4, 164.8, 164.6, 160.1, 156.7, 130.7, 128.9, 88.7, 79.7, 54.8, 54.3, 46.0, 43.3; **MS (m/z):** $[M + H]^+$ 433.45; **HPLC**: (a) H_2O + 0.1% TFA, (b) ACN + 0.1% TFA (5−95% ACN in 12 min at 1.2 mL/min), R_t = 4.49 min, 99.86%.

Synthesis of YK57 and YK56

2-(2-(2-(2-((2-Amino-6-methoxypyrimidin-4-yl)oxy)ethoxy)ethoxy)ethoxy)ethan-1-ol (**16**): To a solution of 10.95 g (56.4 mmol) of tetraethylene glycol in 30 mL of DMF was added 1.35 g (56.40 mmol) of NaH portion wise at 0 ºC and the resulting suspension was allowed to warm to rt. Then 3.0 g (18.8 mmol) of 2-amino-4-chloro-6-methoxypyrimidine (**15**) was added and the reaction mixture heated at 80 ºC for 3 h. The reaction was quenched with methanol and the solvent was removed under reduced pressure. The oily residue was purified by flash chromatography (EtOAc:MeOH, 100:0 to 95:5) to give 5.2 g (87 %) of an oil **16**. **^{1}H NMR (500 MHz, $CDCl_3$)**: δ 5.49 (s, 1H), 4.91 (br s, 2H), 4.40 – 4.37 (m, 2H), 3.83 (s, 3H), 3.81 – 3.78 (m, 2H), 3.75 – 3.72 (m, 2H), 3.72 – 3.64 (m, 8H), 3.63 – 3.60 (m, 2H), 2.79 (br s, 1H); **^{13}C NMR (125 MHz, $CDCl_3$)**: δ 172.4, 171.8, 162.2, 79.9, 72.6, 70.7, 70.6, 70.6, 70.4, 69.5, 65.4, 61.6, 53.8; **MS (*m/z*)**: $[M+H]^+$ 318.02.

2-(2-(2-(2-((2-Fluoro-6-methoxypyrimidin-4-yl)oxy)ethoxy)ethoxy)ethoxy)ethan-1-ol (**17**): 3.2 g (10.1 mmol) of **16** was added to a plastic tube fitted with a stir bar and cooled to 0 °C. Then a solution of HF/pyridine (2.44 ml, 97.6 mmol) was added. After 30 minutes, 1 g (14.64 mmol) of $NaNO_2$ was added in portions over a period of 30 minutes with stirring. It was vigorously stirred for an additional 70 minutes at 0 ºC and at rt for 3 hours. Then 30 ml of CH_2Cl_2 and 10 g of $CaCO_3$ (97.6 mmol) were added and the mixture was stirred for 5 hours at rt. It was then filtered over a cintered disc funnel and the solid washed with EtOAc (4 x 30 ml). The combined filtrate was filtered over celite, concentrated under reduced pressure and the oily residue was purified by flash chromatography (EtOAc:MeOH, 100:0 to 95:5) to give 1.2 g (37 %) of an oil **17**. **^{1}H NMR (500 MHz, $CDCl_3$)**: δ 6.00 (d, *J* = 2.5 Hz, 1H), 4.51 – 4.46 (m, 2H), 3.95 (s, 3H), 3.83 – 3.79 (m, 2H), 3.74 – 3.64 (m, 10H), 3.63 – 3.58 (m, 2H), 2.55 (br s, 1H); **^{13}C NMR (125 MHz, $CDCl_3$)**: δ 173.7 (d, *J* = 15.7 Hz), 173.0 (d, *J* = 15.6 Hz), 162.4 (d, *J* = 216.0 Hz), 88.0 (d, *J* = 7.4 Hz), 72.5, 70.6, 70.5, 70.3, 69.2, 66.6, 61.7, 54.8; **MS (*m/z*)**: $[M+H]^+$ 321.19.

2-(2-(2-(2-((6-Methoxy-2-(4-methylpiperazin-1-yl)pyrimidin-4-yl)oxy)ethoxy)ethoxy)ethoxy) ethan-1-ol (18): 2 g (6.24 mmol) of **17** was dissolved in 40 ml of DMF and 6.25 g (62.44 mmol) of 1-methylpiper- azine was added and heated at 90 °C for 1 h. Solvent and excess reagent were removed under reduced pressure and the oily residue was purified by flash chromatography (CH_2Cl_2:MeOH, 10:1) to give 2.1 g (84 %) of an oil **18**. **^{1}H NMR (500 MHz, $CDCl_3$)**: δ 5.40 (s, 1H), 4.44 – 4.39 (m, 2H), 3.85 (s, 3H), 3.80 (s, 4H), 3.74 – 3.65 (m, 12H), 3.63 – 3.59 (m, 2H), 2.47 – 2.42 (m, 4H), 2.34 (s, 3H); **^{13}C NMR (125 MHz, $CDCl_3$)**: δ 171.9, 171.3, 160.7, 78.5, 72.6, 70.6, 70.5, 70.3, 69.5, 65.0, 61.6, 54.9, 53.4, 46.2, 43.6; **MS (*m/z*)**: $[M+H]^+$ 401.17.

2-(2-(2-(2-((5-Iodo-6-methoxy-2-(4-methylpiperazin-1-yl)pyrimidin-4-yl)oxy)ethoxy)ethoxy) ethoxy) ethan-1-ol (19): To 2 g (4.99 mmol) of **18** dissolved in 40 ml CH_3CN was added 1.93 g (8.59 mmol) of *N*-iodosuccinimide and the solution was stirred for 1.5 h at rt. After completion, the reaction was quenched with 5 percent aqueous sodium thiosulphate solution (50 mL) and the solvent removed in vacuo. The product was then extracted into dichloromethane (3 x 50 mL) and the combined organics were washed with brine (50 mL) and dried over $NaSO_4$. The resulting oily residue was purified by flash chromatography (CH_2Cl_2:MeOH, 10:1) to give 2.3 g (87 %) of an yellow oil **19**. **^{1}H NMR (500 MHz, $CDCl_3$):** δ 4.49 – 4.44 (m, 2H), 3.92 (s, 3H), 3.86 – 3.79 (m, 6H), 3.79 – 3.75 (m, 2H), 3.74 – 3.70 (m, 2H), 3.69 – 3.64 (m, 6H), 3.62 – 3.59 (m, 2H), 2.50 – 2.45 (m, 4H), 2.37 (s, 3H); **^{13}C NMR (125 MHz, $CDCl_3$)**: δ 169.2, 168.6, 160.1, 72.5, 71.0, 70.6, 70.6, 70.3, 69.2, 66.6, 61.6, 54.7, 46.2, 44.5, 43.7; **MS (*m/z*)**: $[M+H]^+$ 527.05.

2-(2-(2-(2-((5-((4,6-Diaminopyrimidin-2-yl)thio)-6-methoxy-2-(4-methylpiperazin-1-yl)pyrimidin-4-yl)oxy)ethoxy)ethoxy)ethoxy)ethan-1-ol (20): A mixture of 1.5 g (2.851 mmol) of **19**, 1.21 g (5.702 mmol) K_3PO_4, 60 mg (0.285 mmol) neocuproine, 54.3 mg (0.285 mmol) copper iodide, and 600 mg (3.421 mmol) 4,6-diamino- 2-mercaptopyrimidine in 30 ml DMF was heated at 150 °C for 3 h. Solvent was removed under reduced pressure and the residue was purified by flash chromatography (CH_2Cl_2:MeOH, 10:1) to give 700 mg (45 %) of **20**. **^{1}H NMR (500 MHz, $CDCl_3/CD_3OD$)**: δ 5.20 (s, 1H), 4.46 (s, 2H), 3.91 – 3.84 (m, 7H), 3.76 (s, 2H), 3.69 (s, 2H), 3.65 – 3.60 (m, 6H), 3.59 – 3.54 (m, 4H), 2.49 (s, 4H), 2.36 (s, 3H); **^{13}C NMR (125 MHz, $CDCl_3/CD_3OD$)**: δ 171.0, 170.4, 169.9, 163.3, 159.7, 80.1, 80.0, 72.3, 70.4, 70.2, 70.1, 69.9, 69.0, 66.3, 61.0, 54.4, 53.8, 45.6, 43.1; **MS (*m/z*)**: $[M + H]^+$ 541.42.

2-(2-(2-(2-((5-((4-Acrylamido-6-aminopyrimidin-2-yl)thio)-6-methoxy-2-(4-methylpiperazin-1-yl) pyrimidin-4-yl)oxy)ethoxy)ethoxy)ethoxy)ethyl acrylate (21): To 0.300 g (0.555 mmol) of **20** in 10 ml of CH_2Cl_2 at 0 °C was added 386 µl (2.77 mmol) of triethylamine. Then 90 µl (1.11 mmol) of acryloyl chloride was added and the reaction was stirred at 0 °C. After 2 hr, additional (90 µl, 1.11 mmol) of acryloyl chloride was added and stirring continued for an additional 1 hr. After completion of reaction, the mixture was concentrated under reduced pressure and the residue purified by flash chromatography (CH_2Cl_2:MeOH, 10:1) to yield 112 mg (31 %) of **21**. **^{1}H NMR (500 MHz, $CDCl_3/CD_3OD$)**: δ 7.03 (s, 1H), 6.45 – 6.35 (m, 2H), 6.26 (dd, *J* = 16.9, 10.3 Hz, 1H), 6.14 (dd, *J* = 17.3, 10.5 Hz, 1H), 5.88 – 5.74 (m, 2H), 4.49 – 4.43 (m, 2H), 4.32 – 4.27 (m, 2H), 3.92 – 3.82 (m, 7H), 3.77 – 3.69 (m, 4H), 3.64 – 3.57 (m, 6H), 3.54 (dd, *J* = 5.4, 3.3 Hz, 2H), 2.53 – 2.44 (m, 4H), 2.37 (s, 3H); **^{13}C NMR (150 MHz, $CDCl_3/CD_3OD$)**: δ 171.8, 171.2, 170.8, 167.1, 165.8, 165.4, 160.5, 157.2, 131.6, 131.3, 129.1, 128.6, 89.1, 80.5, 71.2, 71.0, 70.93, 70.9, 69.7, 69.4, 66.9, 64.3, 55.2, 54.5, 46.1, 43.8; **MS (*m/z*)**: $[M+H]^+$ 649.41.

***N*-(6-Amino-2-((4-(2-(2-(2-(2-hydroxyethoxy)ethoxy)ethoxy)ethoxy)-6-methoxy-2-(4-methylpiperazin -1-yl)pyrimidin-5-yl)thio)pyrimidin-4-yl)acrylamide (22, YK57)**: To 50 mg (0.0647 mmol) of **21** dissolved in 1.6 ml of THF was added 0.4 mL of 0.5 N NaOH at rt and stirred for 6 hours. The reaction mixture was concentrated under reduced pressure and the residue purified by preparatory TLC (CH_2Cl_2:MeOH-NH_3 (7N), 10:1) to yield 22.2 mg (48 %) of **22**. **^{1}H NMR (500 MHz, $CDCl_3$)**: δ 8.52 (br s, 1H), 7.06 (s, 1H), 6.41 (d, *J* = 16.9 Hz, 1H), 6.24 (dd, *J* = 16.9, 10.3 Hz, 1H), 5.76 (d, *J* = 10.2 Hz, 1H), 5.04 (br s, 2H), 4.52 – 4.44 (m, 2H), 3.89 (s, 3H), 3.85 (s, 4H), 3.74 – 3.67 (m, 4H), 3.62 – 3.56 (m, 8H), 3.53 (dd, *J* = 5.5, 3.3 Hz, 2H), 2.47 (s, 4H), 2.36 (s, 3H); **^{13}C NMR (125 MHz, $CDCl_3$)**: δ 171.0, 170.5, 170.3, 164.6, 164.5, 159.8, 156.8, 130.9, 128.7, 88.5, 80.2, 72.7, 70.7, 70.6, 70.5, 70.5, 69.4, 66.3, 61.4, 54.8, 54.2, 46.2, 43.5; **MS (*m/z*):** $[M+H]^+$ 595.34; **HPLC**: (a) H_2O + 0.1% TFA, (b) ACN + 0.1% TFA (5−95% ACN in 12 min at 1.2 mL/min) R_t = 4.25 min, 99.7%.

2-(2-(2-(2-((5-((4-Acrylamido-6-aminopyrimidin-2-yl)thio)-6-methoxy-2-(4-methylpiperazin-1-yl)pyrimidin-4-yl)oxy)ethoxy)ethoxy)ethoxy)ethyl 5-((3a*S*,4*S*,6a*R*)-2-oxohexahydro-1*H*-thieno[3,4-d]imidazol-4-yl)pentanoate (23, YK56): A mixture of 15 mg (0.0252 mmol) of **22**, 21.5 mg (0.088 mmol) D-(+)-biotin, 3.1 mg (0.0252 mmol) DMAP, 41.6 mg (0.2016 mmol) DCC in 10 ml of CH_2Cl_2 was sonicated for 18 hours in a sealed tube. The reaction mixture was concentrated under reduced pressure and the residue purified by preparatory TLC (CH_2Cl_2:MeOH-NH_3 (7N), 10:1) to yield 12.2 mg (64 %) of **23** (**YK56**). **^{1}H NMR (600 MHz, $CDCl_3$)**: δ 9.62 (s, 1H), 7.15 (s, 1H), 6.49 – 6.39 (m, 2H), 6.17 (br s, 1H), 5.73 (dd, *J* = 9.4, 2.2 Hz, 1H), 5.64 (br s, 1H), 5.26 (br s, 2H), 4.60 – 4.52 (m, 2H), 4.47 – 4.42 (m, 1H), 4.41 – 4.37 (m, 1H), 4.25 – 4.13 (m, 2H), 3.88 (br s, 7H), 3.70 – 3.53 (m, 12H), 3.20 – 3.14 (m, 1H), 2.97 – 2.91 (m, 1H), 2.85 (d, *J* = 12.9 Hz, 1H), 2.50 (br s, 4H), 2.38 (s, 3H), 2.27 (t, *J* = 7.5 Hz, 2H), 1.77 – 1.71 (m, 1H), 1.62 – 1.52 (m, 3H), 1.48 – 1.38 (m, 2H); **^{13}C NMR (150 MHz, $CDCl_3$)**: δ 173.6, 171.0, 170.6, 169.8, 165.0, 164.7, 164.1, 159.8, 157.0, 131.0, 128.6, 88.7, 80.1, 70.9, 70.6, 70.5, 69.7, 69.2, 66.1, 63.5, 62.2, 60.3, 55.7, 54.8, 54.2, 46.1, 43.6, 40.5, 33.7, 28.4, 28.2, 24.8; **MS (*m*/*z*)**: $[M+H]^+$ 821.49; **HPLC**: (a) H_2O + 0.1% TFA, (b) ACN + 0.1% TFA (5−95% ACN in 12 min at 1.2 mL/min) R_t = 4.91 min, 95.02%.

<u>Synthesis of YK54 and YK55</u>

2-(2-(2-(2-(4-(5-((4,6-Diaminopyrimidin-2-yl)thio)-4,6-dimethoxypyrimidin-2-yl)piperazin-1-yl)ethoxy) ethoxy)ethoxy)ethan-1-ol (24): Compound **24** was synthesized as reported previously[1]. **^{1}H NMR (600 MHz, $CDCl_3$/DMSO-*d*$_6$):** δ 5.28 (br s, 5H), 3.89 (br s, 4H), 3.87 (s, 6H), 3.70 (t, *J* = 5.4 Hz, 2H), 3.69 – 3.66 (m, 2H), 3.66 – 3.63 (m, 8H), 3.59 – 3.56 (m, 2H), 2.71 (br s, 2H), 2.65 (br s, 4H); **^{13}C NMR (150 MHz, $CDCl_3$/CD_3OD):** δ 170.2, 169.0, 162.3, 159.1, 78.7, 71.7, 69.6, 69.5, 69.2, 69.1, 67.2, 60.3, 56.7, 53.2, 52.3, 42.2; **MS (*m*/*z*)**: $[M+H]^+$ 541.30.

2-(2-(2-(2-(4-(5-((4-Acrylamido-6-aminopyrimidin-2-yl)thio)-4,6-dimethoxypyrimidin-2-yl)piperazin-1-yl)ethoxy)ethoxy)ethoxy)ethyl acrylate (25): To 50 mg (0.092 mmol) of **24** in 5 ml of CH_2Cl_2 at 0 °C was added 65 µl (0.462 mmol) of Et_3N. 20.4 µl (0.253 mmol) of acryloyl chloride was added at 0 °C. After 1 hour an additional 20.4 µl (0.253 mmol) of acryloyl chloride was added. This was repeated three more times for a total reaction time of 7 h (total acryloyl chloride, 102 µl, 1.265 mmol). The reaction mixture was concentrated under reduced pressure and the residue purified by preparatory TLC (CH_2Cl_2:MeOH, 10:1) to yield 24 mg (40 %) of **25**. **^{1}H NMR (500 MHz, $CDCl_3$)**: δ 8.36 (br s, 1H), 7.04 (s, 1H), 6.41 (t, *J* = 17.0 Hz, 2H), 6.23 – 6.07 (m, 2H), 5.79 (dd, *J* = 43.1, 10.3 Hz, 2H), 4.98 (br s, 2H), 4.34 – 4.25 (m, 2H), 3.87 (br s, 10H), 3.76 – 3.70 (m, 2H), 3.66 (br s, 10H), 2.66 (br s, 2H), 2.57 (br s, 4H); **^{13}C NMR (125 MHz, $CDCl_3$)**: δ 171.1, 170.6, 166.2, 164.4, 164.3, 160.0, 156.8, 131.1, 130.8, 128.8, 128.3, 88.5, 79.9, 70.7, 70.6, 70.4, 69.2, 68.8, 63.7, 57.9, 54.2, 53.4, 43.6; **MS (*m*/*z*)**: $[M+H]^+$ 649.23.

***N*-(6-Amino-2-((2-(4-(2-(2-(2-(2-hydroxyethoxy)ethoxy)ethoxy)ethyl)piperazin-1-yl)-4,6-dimethoxy pyrimidin-5-yl)thio)pyrimidin-4-yl)acrylamide (26, YK54):** To 10 mg (0.0168 mmol) of **25** dissolved in 1.6 ml of THF was added 0.4 mL of 0.5 N NaOH at rt and stirred for 5 hours. The reaction mixture was concentrated under reduced pressure and the residue purified by preparatory TLC (CH_2Cl_2:MeOH, 10:1) to yield 4.2 mg (46 %) of **26** (**YK54**). **^{1}H NMR (600 MHz, $CDCl_3$)**: δ 8.61 (br s, 1H), 7.02 (s, 1H), 6.36 (dd, *J* = 16.9, 0.8 Hz, 1H), 6.09 (dd, *J* = 16.8, 10.3 Hz, 1H), 5.70 (d, *J* = 11.1 Hz, 1H), 5.15 (br s, 2H), 3.85 (br s, 10H), 3.75 – 3.71 (m, 2H), 3.69 – 3.63 (m, 11H), 3.63 – 3.59 (m, 2H), 2.67 (t, *J* = 5.3 Hz, 2H), 2.58 (br s, 4H); **^{13}C NMR (150 MHz, $CDCl_3$):** δ 171.1, 170.7, 164.3, 164.2, 159.9, 156.8, 130.7, 128.7, 88.8, 79.6, 72.8, 70.6, 70.2, 70.1, 68.3, 61.4, 58.0, 54.1, 53.3, 43.4; **MS (*m*/*z*)**: $[M+H]^+$ 595.34; **HPLC**: (a) H_2O + 0.1% TFA, (b) ACN + 0.1% TFA (5−95% ACN in 12 min at 1.2 mL/min) R_t = 4.57 min, 98.17%.

2-(2-(2-(2-(4-(5-((4,6-Diaminopyrimidin-2-yl)thio)-4,6-dimethoxypyrimidin-2-yl)piperazin-1-yl)ethoxy)ethoxy)ethoxy)ethyl 5-((3aS,4S,6aR)-2-oxohexahydro-1H-thieno[3,4-d]imidazol-4-yl) pentanoate (27): 50.0 mg (0.0925 mmol) of **24**, 80.0 mg (0.3236 mmol) D-(+)-biotin, 11.3 mg (0.0925 mmol) DMAP, 153 mg (0.74 mmol) DCC in 15 ml of CH_2Cl_2 was sonicated for 14 hours in a sealed tube. The reaction mixture was evaporated to dryness and the residue was column chromatographed (CH_2Cl_2:MeOH-NH_3 (7N), 20:1 to 10:1) to give impure **27** which was purified by preparatory TLC (CH_2Cl_2:MeOH-NH_3 (7N), 10:1) to yield 53 mg (75 %) of **27**. **^{1}H NMR (600 MHz, $CDCl_3$/CD_3OD):** δ 5.19 (s, 1H), 4.50 (dd, *J* = 7.8, 4.9 Hz, 1H), 4.30 (dd, *J* = 7.8, 4.6 Hz, 1H), 4.24 – 4.21 (m, 2H), 3.88 (br s, 10H), 3.72 – 3.62 (m, 12H), 3.18 – 3.13 (m, 1H), 2.90 (dd, *J* = 11.5, 6.5 Hz, 1H), 2.74 (d, *J* = 12.8 Hz, 1H), 2.67 (t, *J* = 5.7 Hz, 2H), 2.61 – 2.56 (m, 4H), 2.36 (t, *J* = 7.4 Hz, 2H), 1.75 – 1.59 (m, 4H), 1.50 – 1.38 (m, 2H); **^{13}C NMR (150 MHz, $CDCl_3$/CD_3OD):** δ 173.9, 171.2, 170.3, 164.0, 163.9, 163.5, 163.4, 160.1, 80.7, 79.9, 70.6, 70.6, 70.5, 70.4, 69.2, 68.8, 63.5, 62.0, 60.1, 57.8, 55.5, 54.2, 53.4, 43.5, 40.5, 33.8, 28.5, 28.3, 24.7; **MS (m/z):** [M+H]+ 767.36.

2-(2-(2-(2-(4-(5-((4-Acrylamido-6-aminopyrimidin-2-yl)thio)-4,6-dimethoxypyrimidin-2-yl)piperazin-1-yl)ethoxy)ethoxy)ethoxy)ethyl 5-((3aS,4S,6aR)-2-oxohexahydro-1H-thieno [3,4-d]imidazol-4-yl)pentanoate (28, YK5-B): To 35 mg (0.046 mmol) of **27** in 10 ml of CH_2Cl_2 at 0 °C was added 32 µl (0.225 mmol) of Et_3N. Then 7.5 µl (0.092 mmol) of acryloyl chloride was added at 0 °C. After 1 hour an additional 7.5 µl (0.0913 mmol) of acryloyl chloride was added. This was repeated two more times for a total reaction time of 5 hours (total acryloyl chloride, 30 µl, 0.368 mmol). The reaction mixture was concentrated under reduced pressure and the residue purified by preparatory TLC (CH_2Cl_2:MeOH-NH_3 (7N), 10:1) to yield 18 mg (48 %) of **YK5-B** (**28**). **^{1}H NMR (600 MHz, $CDCl_3$)**: δ 9.28 (s, 1H), 7.09 (s, 1H), 6.39 (dd, *J* = 16.9, 1.5 Hz, 1H), 6.32 (dd, *J* = 16.9, 10.0 Hz, 1H), 6.04 (br s, 1H), 5.74 – 5.70 (m, 1H), 5.61 (s, 1H), 5.26 (br s, 2H), 4.53 (dd, *J* = 7.6, 5.1 Hz, 1H), 4.37 – 4.31 (m, 1H), 4.26 – 4.16 (m, 2H), 3.86 (s, 10H), 3.70 – 3.61 (m, 12H), 3.17 – 3.12 (m, 1H), 2.92 – 2.88 (dt, *J* = 9.0, 4.5 Hz, 1H), 2.79 (d, *J* = 12.8 Hz, 1H), 2.68 (br s, 2H), 2.59 (br s, 4H), 2.30 (t, *J* = 7.6 Hz, 2H), 1.79 – 1.72 (m, 1H), 1.65 – 1.56 (m, 3H), 1.49 – 1.37 (m, 2H); **^{13}C NMR (150 MHz, $CDCl_3$)**: δ 173.6, 171.0, 170.1, 164.8, 164.6, 163.8, 159.9, 156.9, 131.0, 128.6, 88.7, 79.6, 70.7, 70.6, 70.5, 70.4, 69.2, 68.7, 63.4, 62.1, 60.2, 57.7, 55.5, 54.2, 53.1, 43.7, 40.5, 33.7, 28.4, 28.3, 24.7; **MS (m/z)**: $[M+H]^+$ 821.47; **HPLC**: (a) H_2O + 0.1% TFA, (b) ACN + 0.1% TFA (5−95% ACN in 12 min at 1.2 mL/min) R_t = 5.11 min, 99.61%.

Synthesis of epiTCO

epiTCO (**34**) was synthesized with a slight modification of a previously described synthetic protocol for epichaperome probes[3]. Briefly, the dehydrative coupling of 3-(Boc-amino)-1-propanol with 8-((6-iodobenzo[*d*][1,3]dioxol-5-yl)thio)-9*H*-purin-6-amine in the presence of PPh_3 and DEAD gave a Boc-protected intermediate in 52% yield, which underwent boc-deprotection with TFA**.** The coupling reaction of free aliphatic amine from the previous step and 4-(*tert*-butoxycarbonylamino)butyric acid with DCC followed by removal of the Boc-group provided an intermediate in 76% yield, which was finally coupled with TCO-NHS ester in the presence of triethylamine in dark to obtain **epiTCO** (**34**) in 68% yield.

***tert*-Butyl(3-(6-amino-8-((6-iodobenzo[d][1,3]dioxol-5-yl)thio)-9H-purin-9-yl)propyl)carbamate** (**30**)[4]: To a stirred of 8-((6-iodobenzo[*d*][1,3]dioxol-5-yl)thio)-9*H*-purin-6-amine (**29**) (800 mg, 1.936 mmol), *N*-tert-butoxycarbonyl-3-aminopropanol (510 mg, 2.904), and PPh_3 (1.02 g, 3.872 mmol) in dry CH_2Cl_2 (20 mL) was added DEAD (2.2 M in toluene, 4.4 ml, 9.68 mmol). After 2 h, the reaction mixture was concentrated under reduced pressure and the residue purified by flash chromatography (CH_2Cl_2:MeOH, 10:1) to yield 570 mg (52 %) of **30. ^{1}H NMR (500 MHz, $CDCl_3$)**: δ 8.29 (s, 1H), 7.31 (s, 1H), 6.91 (s, 1H), 6.21 (brs, 2H), 6.00 (s, 2H), 5.54 (s,

1H), 4.28 (t, *J* = 5.9 Hz, 2H), 3.04 (s, 2H), 1.96 – 1.88 (m, 2H), 1.45 (s, 9H); **^{13}C NMR (125 MHz, $CDCl_3$)**: δ 156.0, 154.8, 152.3, 151.5, 149.3, 149.2, 146.5, 127.2, 119.7, 119.3, 112.6, 102.4, 91.7, 79.3, 41.0, 36.9, 29.8, 28.5; **MS (*m/z*)**: $[M + H]^+$ 571.14.

9-(3-Aminopropyl)-8-((6-iodobenzo[d][1,3]dioxol-5-yl)thio)-9*H*-purin-6-amine (31): To a stirred solution of **30** (300 mg, 0.526 mmol) in dry CH_2Cl_2 (5.0 ml) was added TFA (500 µL). The solution was stirred at rt for 3 h. The solvent was removed under reduced pressure and the residue purified by preparatory TLC (CH_2Cl_2:MeOH, 10:1) to yield 210 mg (81 %) of **31**. **^{1}H NMR (500 MHz, $CDCl_3/CD_3OD$)**: δ 8.22 (s, 1H), 7.39 (s, 1H), 7.06 (s, 1H), 6.06 (s, 2H), 4.33 – 4.24 (m, 2H), 2.72 – 2.60 (m, 2H), 2.04 – 1.90 (m, 2H); **^{13}C NMR (125 MHz, $CDCl_3/CD_3OD$)**: δ 154.5, 152.5, 151.5, 149.9, 149.6, 147.5, 125.9, 119.6, 119.5, 113.9, 102.7, 94.1, 41.0, 38.2, 32.7; **MS (*m/z*)**: $[M + H]^+$ 470.87.

***tert*-Butyl (4-((3-(6-amino-8-((6-iodobenzo[d][1,3]dioxol-5-yl)thio)-9H-purin-9-yl)propyl)amino)-4-oxo butyl)carbamate (32):** A mixture of **31** (110 mg, 0.234 mmol), 4-((*tert*-butoxycarbonyl)amino)butanoic acid (72 mg, 0.354 mmol), DCC (96.5 mg, 0.467 mmol) and a catalytic amount of DMAP (2.8 mg, 0.0234 mmol) in CH_2Cl_2 was stirred at room temperature overnight. The reaction mixture was concentrated under reduced pressure and the resulting residue was purified by preparatory TLC (CH_2Cl_2:MeOH-NH_3 (7N), 20:1) to give 92 mg (60%) of **32**. **^{1}H NMR (500 MHz, $CDCl_3$)**: δ 8.33 (s, 1H), 7.32 (s, 1H), 7.21 (s, 1H), 6.94 (s, 1H), 6.01 (s, 2H), 5.69 (br s, 2H), 4.89 (s, 1H), 4.28 (t, *J* = 6.3 Hz, 2H), 3.20 (dd, *J* = 13.3, 7.3 Hz, 2H), 3.13 (dd, *J* = 12.1, 6.1 Hz, 2H), 2.29 (t, *J* = 7.0 Hz, 2H), 1.99 – 1.92 (m, 2H), 1.90 – 1.82 (m, 2H), 1.43 (s, 9H); **^{13}C NMR (125 MHz, $CDCl_3/CD_3OD$)**: δ 173.8, 156.6, 154.0, 151.8, 150.6, 149.6, 149.1, 147.4, 124.9, 119.1, 118.9, 113.5, 102.3, 93.9, 78.7, 40.9, 39.2, 35.9, 33.0, 28.6, 27.6, 25.5; **MS (*m/z*)**: $[M+H]^+$ 656.11.

4-Amino-*N*-(3-(6-amino-8-((6-iodobenzo[d][1,3]dioxol-5-yl)thio)-9H-purin-9-yl)propyl)butanamide (33): To a stirred CH_2Cl_2 (2.0 ml) solution of **32** (50 mg, 0.0763 mmol) was added TFA (200 µL). The solution was stirred at rt for 1h. The reaction mixture was concentrated under reduced pressure and the resulting residue was purified by preparatory TLC (CH_2Cl_2:MeOH-NH_3 (7N), 20:1) to give 32 mg (76%) of **33**.**^{1}H NMR (500 MHz, $CDCl_3/CD_3OD$)**: δ 8.21 (s, 1H), 7.40 (s, 1H), 7.08 (s, 1H), 6.07 (s, 2H), 4.27 (t, *J* = 7.1 Hz, 2H), 3.25 (t, *J* = 6.3 Hz, 2H), 2.74 (t, *J* = 7.0 Hz, 2H), 2.30 (t, *J* = 7.4 Hz, 2H), 2.06 – 1.98 (m, 2H), 1.85 – 1.77 (m, 2H); **^{13}C NMR (150 MHz, $CDCl_3/CD_3OD$)**: δ 173.4, 154.4, 152.4, 151.3, 150.0, 149.5, 147.8, 125.5, 119.7, 119.5, 114.1, 102.7, 94.5, 41.2, 40.4, 36.1, 33.6, 29.0, 26.8; **MS (*m/z*)**: $[M+H]^+$ 556.14.

(*E*)-Cyclooct-4-en-1-yl (4-((3-(6-amino-8-((6-iodobenzo[d][1,3]dioxol-5-yl)thio)-9H-purin-9-yl)propyl) amino)-4-oxobutyl)carbamate (34, epiTCO): A mixture of **33** (11 mg, 0.0198 mmol), TCO-NHS ester (6.3 mg, 0.0237 mmol) and TEA (6 µL, 0.0396 mmol) in DMF was stirred at rt for 3 h in dark. The reaction mixture was concentrated under reduced pressure and the resulting residue was purified by flash chromatography (CH_2Cl_2:MeOH, 20:1) to give 9.5 mg (68%) of compound **34** (**epiTCO**). **^{1}H NMR (600 MHz, $CDCl_3/CD_3OD$)**: δ 8.25 (s, 1H), 7.38 (s, 1H), 7.05 (s, 1H), 6.05 (s, 2H), 5.60 – 5.44 (m, 2H), 4.30 (dd, *J* = 10.2, 5.7 Hz, 1H), 4.25 (t, *J* = 6.8 Hz, 2H), 3.25 – 3.15 (m, 4H), 2.36 – 2.25 (m, 5H), 2.03 – 1.96 (m, 3H), 1.96 – 1.81 (m, 5H), 1.77 – 1.67 (m, 2H), 1.58 – 1.48 (m, 1H); **^{13}C NMR (150 MHz, $CDCl_3$)**: δ 172.6, 156.5, 154.5, 153.0, 151.9, 149.3, 149.2, 146.7, 134.9, 133.0, 127.2, 120.0, 119.3, 112.7, 102.4, 91.9, 80.5, 41.2, 40.7, 40.3, 38.7, 35.4, 34.3, 33.9, 32.5, 30.9, 29.1, 25.9; **MS (*m/z*)**: $[M + H]^+$ 708.32; **HPLC**: (a) H_2O + 0.1% TFA, (b) ACN + 0.1% TFA (5−95% ACN in 12 min at 1.2 mL/min) R_t = 6.47 min, 98.94%.

Supplementary Note 2. Characterization of YKs in reconstituted systems.

HSP70s undergo large conformational changes regulated by ATP and ADP binding and further influenced by co-chaperones (**Supplementary Fig. 16a**)[5]. The allosteric pocket in HSP70s targeted by YKs is formed in a conformation resembling that of ADP bound HSP70s, in which the substrate binding domain captures interactor proteins (i.e., substrates)[6]. YKs thus would stabilize this state, causing it to hold on to substrate longer (**Supplementary Fig. 16b**). This mode of binding for YKs to HSP70s was computationally predicted, experimentally demonstrated in cellulo, biochemically and functionally[7], and further through medicinal chemistry and structure-activity studies[2, 8]. We provide now further evidence in reconstituted systems using HSP70s, HSP70 regulatory co-chaperones (i.e., Bag1 and DJA2) and an HSP70s substrate, alone or combined. We designed several studies observing the effect of YKs on substrate release, ATP and ADP binding, and hydrolysis of ATP.

For substrate binding, we used fluorescence anisotropy to monitor binding of an N-terminally FAM-labeled peptide substrate of HSP70s. The peptide sequence TSLLMVIMG was derived from the cystic fibrosis transmembrane regulator (CFTR), a known HSP70 substrate[9]. Complexes of HSC70 or HSP70 were created with FAM-peptide in the presence of ADP. They were then quickly mixed with ATP, and the anisotropy signal was monitored over time (**Supplementary Fig. 16c**). The anisotropy data was used to calculate the amount of peptide bound to HSP70. We observed a decrease in anisotropy indicating a release of FAM-peptide from HSP70 in the presence of ATP, as expected. Without ATP, about 100% of peptide remained bound to HSC70 and HSP70 over the course of the experiment (see Source Data). YK198 significantly decreased the amount of HSP70s-bound peptide released by ATP and altered the rate of release (**Supplementary Fig. 16c**). The HSC70 reactions were best fit with a two-phase exponential decay model. The first-rate constant k_{fast} was lowered from 0.014 s^{-1} to 0.010 s^{-1} by YK198, and the second rate constant k_{slow} also decreased from 9.5×10^{-5} s^{-1} to 3.9×10^{-5} s^{-1}.

ATP binding is necessary for the efficient release of substrate[5]. We used a stopped flow device to quickly mix HSC70 or HSP70 with a fluorescently labeled ATP (i.e., MABA-ATP) and monitored an increase in fluorescence over time, which indicated ATP binding to HSP70s (**Supplementary Fig. 16d**). The data was then fit to a single exponential association curve, and an association rate constant was determined for each condition tested. We found YK198 binding to HSP70s significantly slowed ATP insertion into the HSP70s nucleotide-binding pocket (**Supplementary Fig. 16d** and Source Data). We obtained a k_{on} of 2.0×10^5 $M^{-1}s^{-1}$ for ATP binding to HSC70, and 2.6×10^5 $M^{-1}s^{-1}$ for HSP70. With YK198, the k_{on} for ATP binding was 8.3×10^4 $M^{-1}s^{-1}$ for HSC70, and 1.4×10^5 $M^{-1}s^{-1}$ for HSP70, which is between 1.5 and 2 times lower than vehicle.

Next, we tested ADP release in the presence of YK198 (**Supplementary Fig. 16e**), because ADP release is required before ATP can access the nucleotide-binding pocket[5]. Towards this, MABA-ADP complexes with HSC70 or HSP70 were formed, and MABA-ADP release in the presence or absence of Bag1 was monitored in a stopped flow device over time by a decrease in fluorescence, indicating dissociation of the complex. The fluorescence data were fit to a single exponential dissociation curve and off-rate constants for ADP were determined. With no nucleotide exchange factor, the ADP k_{off} for HSC70 was 0.049 s^{-1} and with YK198 it was 0.030 s^{-1} so there was only a minor effect of the drug. Similarly, for HSP70, the ADP k_{off} was 0.11 s^{-1} and with YK198 it was 0.084 s^{-1} with only a small effect on ADP release. ADP release was accelerated by the nucleotide exchange factor, as expected, but YK198 had no significant impact on the ADP release rate (**Supplementary Fig. 16e**).

We next addressed the steady-state ATPase activity of HSP70s in the presence of YK198. The intrinsic ATPase activity in this system was measured to be 0.038 μM ADP/μM HSC70/s. The addition of the isolated J domain of DJA2 (DJA2-J) stimulated ATP hydrolysis and the ATPase activity increased almost two-fold to 0.069 μM ADP/μM HSC70/s, as expected[10]. However, addition of YK198 did not change ATPase rates significantly (**Supplementary Fig. 16f,g**). To specifically confirm that YK198 had no effect on the ATP hydrolysis step, we performed single-turnover ATPase assays with HSC70 and HSP70. With HSC70 alone, the rate of catalysis was 0.022 s^{-1} and addition of DJA2-J stimulated the rate to 0.032 s^{-1}. Addition of YK198 had no significant effect on ATP hydrolysis by HSP70s, with or without DJA2-J (**Supplementary Fig. 16f,h**).

Therefore, our data show the biochemical effect of YK198 on HSP70s manifests majorly at the allosteric release of substrate, impairing substrate release and delaying ATP insertion. YK198 only minorly affects the chaperone ATPase activity of HSP70s, consistent with the specific effect on epiHSP70s over HSP70s observed in cellulo and further confirming the proposed mechanism of action.

Protein purification. pPROEX-HTa (Clontech) expression vector encoding human HSC70, HSP70, DJA2 J-domain, and Bag1 were as previously described[9, 10, 11]. His-tagged HSC70, HSP70, Bag1 and DJA2 J-domain were purified as previously described[9, 10, 11]. Briefly, the expression vectors were grown in Rosetta 2 *Escherichia coli* cells (Novagen), and protein expression was induced with 1 mM isopropyl-β-d-thiogalactopyranoside at 30°C. The cells were harvested and resuspended in buffer containing 750 mM NaCl, 60 mM imidazole and 20 mM KH_2PO_4, pH 7.5, with Complete protease inhibitors (Roche Diagnostics, Indianapolis, IN). Cells were lysed by cavitation in a French press, and the cell debris was removed by centrifugation. The supernatant was loaded onto a 5-mL nickel-Sepharose high-performance column (GE Healthcare, Little Chalfont, Buckinghamshire, United Kingdom), and proteins were eluted with buffer containing 1 M imidazole, 500 mM NaCl, and 20 mM KH_2PO_4, pH 7.5. Peak fractions were loaded on a Superdex 200 Hi-Load 16/60 column (GE Healthcare) and eluted with buffer HS (500 mM NaCl, 20 mM HEPES-KOH, pH 7.5, and 5 mM $MgOAc_2$). Nonaggregated peak fractions were collected, and the yield was determined by absorbance at 280 nm. HSC70 was expressed for 4 h at 30°C and purified on nickel-Sepharose high-performance equilibrated in buffer containing 500 mM NaCl, 20 mM imidazole, and 20 mM KH_2PO_4, pH 7.5, and eluted in buffer containing 300 mM imidazole and 20 mM KH_2PO_4, pH 7.5. HSC70 was further purified by ion exchange on a Mono Q 5/50 GL column and gel filtration on a Superdex 200 Hi-Load 16/60 column (GE Healthcare) equilibrated in buffer G (100 mM KOAc, 20 mM HEPES-KOH, pH 7.5, and 5 mM $MgOAc_2$). His tags were removed by digestion with His-tagged TEV protease 4 °C overnight followed by repurification on nickel-Sepharose.

Supplementary References

1. Kang, Y., Taldone, T., Rodina, A., Patel, P. D. & Chiosis, G. Heat shock protein binding compounds, compositions, and methods for making and using same. U.S. patent EP2467142 A2467142 (June 2012).
2. Kang, Y. et al. Heat shock protein 70 inhibitors. 1. 2,5'-thiodipyrimidine and 5-(phenylthio)pyrimidine acrylamides as irreversible binders to an allosteric site on heat shock protein 70. *J. Med. Chem.* **57**, 1188-1207 (2014).
3. Taldone, T. et al. Synthesis and evaluation of cell-permeable biotinylated PU-H71 derivatives as tumor Hsp90 probes. *Beilstein J. Org. Chem.* **9**, 544-556 (2013).

4. Yoshii, T., Mizusawa, K., Takaoka, Y. & Hamachi, I. Intracellular protein-responsive supramolecules: protein sensing and in-cell construction of inhibitor assay system. *J. Am. Chem. Soc.* **136**, 16635-16642 (2014).
5. Rosenzweig, R., Nillegoda, N. B., Mayer, M. P. & Bukau, B. The Hsp70 chaperone network. *Nat. Rev. Mol. Cell Biol.* **20**, 665-680 (2019).
6. Shrestha, L., Patel, H. J. & Chiosis, G. Chemical Tools to Investigate Mechanisms Associated with HSP90 and HSP70 in Disease. *Cell Chem. Biol.* **23**, 158-172 (2016).
7. Rodina, A. et al. Identification of an allosteric pocket on human hsp70 reveals a mode of inhibition of this therapeutically important protein. *Chem. Biol.* **20**, 1469-1480 (2013).
8. Taldone, T. et al. Heat shock protein 70 inhibitors. 2. 2,5'-thiodipyrimidines, 5-(phenylthio)pyrimidines, 2-(pyridin-3-ylthio)pyrimidines, and 3-(phenylthio)pyridines as reversible binders to an allosteric site on heat shock protein 70. *J. Med. Chem.* **57**, 1208-1224 (2014).
9. Baaklini, I., Goncalves, C. C., Lukacs, G. L. & Young, J. C. Selective Binding of HSC70 and its Co-Chaperones to Structural Hotspots on CFTR. *Sci. Rep.* **10**, 4176 (2020).
10. Baaklini, I., Wong, M. J., Hantouche, C., Patel, Y., Shrier, A. & Young, J. C. The DNAJA2 substrate release mechanism is essential for chaperone-mediated folding. *J. Biol. Chem.* **287**, 41939-41954 (2012).
11. Tzankov, S., Wong, M. J., Shi, K., Nassif, C. & Young, J. C. Functional divergence between co-chaperones of Hsc70. *J. Biol. Chem.* **283**, 27100-27109 (2008).
