# Supplementary material for: Systems-level analyses of protein-protein interaction network dysfunctions via epichaperomics identify cancer-specific mechanisms of stress adaptation
Source: Nat Commun. 2023 Jun 23;14:3742. doi: 10.1038/s41467-023-39241-7 (PMC10290137; doi:10.1038/s41467-023-39241-7)
Supplement: Supplementary file 3 — Description to Additional Supplementary Information [file 41467_2023_39241_MOESM3_ESM.pdf]

Description of Additional Supplementary Data

Supplementary Data 1. Input datasets, protein quality control assessments and Reactome pathway analyses related to Figures 5d,7a-c,9a,c and Supplementary Figure 17a,19,20,28.

Supplementary Data 2. epiHSP70s and epiHSP90s interactor datasets and network topology analyses associated with Supplementary Figures 17b,c,18.

Supplementary Data 3. Input datasets and Reactome pathway analyses related to Figure 7d.
