# Supplementary material for: Systems-level analyses of protein-protein interaction network dysfunctions via epichaperomics identify cancer-specific mechanisms of stress adaptation
Source: Nat Commun. 2023 Jun 23;14:3742. doi: 10.1038/s41467-023-39241-7 (PMC10290137; doi:10.1038/s41467-023-39241-7)
Supplement: Supplementary file 7 — Reporting Summary [file 41467_2023_39241_MOESM7_ESM.pdf]

nature portfolio

Corresponding author(s): Gabriela Chiosis and Tai Wang

Last updated by author(s): May 17, 2023

Reporting Summary

Nature Portfolio wishes to improve the reproducibility of the work that we publish. This form provides structure for consistency and transparency in reporting. For further information on Nature Portfolio policies, see our Editorial Policies and the Editorial Policy Checklist.

Statistics

For all statistical analyses, confirm that the following items are present in the figure legend, table legend, main text, or Methods section.

n/a	Confirmed	
☐	☒	The exact sample size (*n*) for each experimental group/condition, given as a discrete number and unit of measurement
☐	☒	A statement on whether measurements were taken from distinct samples or whether the same sample was measured repeatedly
☐	☒	The statistical test(s) used AND whether they are one- or two-sided<br>*Only common tests should be described solely by name; describe more complex techniques in the Methods section.*
☒	☐	A description of all covariates tested
☐	☒	A description of any assumptions or corrections, such as tests of normality and adjustment for multiple comparisons
☐	☒	A full description of the statistical parameters including central tendency (e.g. means) or other basic estimates (e.g. regression coefficient) AND variation (e.g. standard deviation) or associated estimates of uncertainty (e.g. confidence intervals)
☐	☒	For null hypothesis testing, the test statistic (e.g. *F*, *t*, *r*) with confidence intervals, effect sizes, degrees of freedom and *P* value noted<br>*Give P values as exact values whenever suitable.*
☒	☐	For Bayesian analysis, information on the choice of priors and Markov chain Monte Carlo settings
☒	☐	For hierarchical and complex designs, identification of the appropriate level for tests and full reporting of outcomes
☐	☒	Estimates of effect sizes (e.g. Cohen's *d*, Pearson's *r*), indicating how they were calculated

Our web collection on statistics for biologists contains articles on many of the points above.

Software and code

Policy information about availability of computer code

Data collection	As described in the Methods, the identity and purity of each product was characterized by MS, HPLC, TLC, and NMR. 1H/13C NMR spectra were recorded on either a Bruker 500 or 600 MHz instrument. High resolution mass spectra were recorded on a Waters LCT Premier system. Low resolution mass spectra were obtained on Waters Acquity Ultra Performance LC with electrospray ionization and SQ detector. HPLC analysis was done on Waters Autopurification system with PDA, MicroMass ZQ and ELSD detector and a reversed phase column (Waters X-Bridge C18, 4.6 x 150 mm, 5 µm) eluted with water/acetonitrile gradients, containing 0.1% TFA. Fluorescence was visualized under a confocal microscope (Zeiss, LSM880). IncuCyte live-cell microscopy system (Essen BioScience) was used to evaluate cell confluency. For flow cytometry we used a LSRFortessa instrument (BD Biosciences). Time lapse microscopy was performed using the Axio Observer (ZEISS). For quantitative proteomics, samples were analyzed by LC-MS/MS with a Q Exactive High Field Orbitrap, and resulting spectra searched with MaxQuant using its corresponding TMT label as variable modifications on N-terminus and lysine. Label free proteomics analyses were performed using a Q Exactive mass spectrometer coupled to a Thermo Scientific EASY-nLC 1000. The mass spectrometer was operated in data-dependent analysis (DDA) mode with survey scans acquired at a resolution of 70,000 over a scan range of 300-1750 m/z. Fluorescence anisotropy experiments were performed on the FluoroMax HORIBA spectro-fluorometer and data was collected with the FluorEssence software. Radiolabeled ATP and ADP were captured on a phosphorimaging screen then detected on a Typhoon scanner and quantified on the Alpha View.
Data analysis	Prism 9 was used for statistical testings (t-tests and ANOVA), and data fitting and analysis. ImageJ (Version 2.0.0) was used for western blot quantification and image analyses. The image analysis were done using ZenBlue software (Version 3.5) (Zeiss). Flow cytometry data were analyzed using FlowJo software (Version 10.0) (FlowJo LLC). For protein identification by MS, all mass spectra were first converted to mgf peak list format using Proteome Discoverer 1.4 and the resulting mgf files searched using Mascot (version 2.7.00). The Mascot search result was finally imported into Scaffold (Proteome Software, Inc., Portland, OR; version 4.11) to further analyze tandem mass spectrometry (MS/MS) based protein and peptide identifications. X! Tandem (The GPM, thegpm.org; version CYCLONE (2010.12.01.1) was then performed and its results were merged with those from Mascot. The mass spectra files were also subjected to Label-Free Quantitation (LFQ) using MaxQuant

proteomics data analysis workflow (version 1.6.0.1) with the Andromeda search engine. For TMT experiments, all data were analyzed with the MaxQuant proteomics data analysis workflow (version 1.5.5.1) with the Andromeda search engine. Following MaxQuant analysis, the protein and peptide .txt files were imported into Perseus (version1.5.6.0) software which was used for the statistical analysis of all the proteins identified. All statistics related to proteomics analyses were performed using R (version 3.3.2). For pathway and interactome visualization we used Cytoscape (v3.40). Bioinformatics analyses were performed using Bioconductor v3.4 packages 'clusterProfiler' and 'ReactomePA' (for pathway enrichment) or R packages 'missForest' (for missing value imputation) and 'limma' (for .differential expression/connectivity). Scripts were deposited in Zenodo [DOI: https://doi.org/10.5281/zenodo.7416220]. Functional enrichment analyses on proteins downregulated in each treatment condition were also performed using gProfiler's web platform101 (Version e106_eg53_p16_65fcd97) .

For manuscripts utilizing custom algorithms or software that are central to the research but not yet described in published literature, software must be made available to editors and reviewers. We strongly encourage code deposition in a community repository (e.g. GitHub). See the Nature Portfolio guidelines for submitting code & software for further information.

Data

Policy information about availability of data

All manuscripts must include a data availability statement. This statement should provide the following information, where applicable:

- Accession codes, unique identifiers, or web links for publicly available datasets
- A description of any restrictions on data availability
- For clinical datasets or third party data, please ensure that the statement adheres to our policy

The source data underlying all main and supplementary figures are provided with this paper as a Source Data file. Datasets and analytics associated with epichaperomics and proteomics analyses are available in the Supplementary Information as Supplementary Data 1 through 3. LC-MS data (i.e. proteomics and epichaperomics raw mass spectrometry data, peak lists, and results) that support the findings of this study are deposited in MassIVE and can be retrieved with the accession code MSV000087540 [ftp://massive.ucsd.edu/MSV000087540/] and the ProteomeXchange Consortium identifier PXD042262 [https://proteomecentral.proteomexchange.org/cgi/GetDataset?ID=PXD042262]. Cytoscape files have been deposited in Zenodo [https://doi.org/10.5281/zenodo.7433980]103. The PPI data were obtained from BioGrid (https://thebiogrid.org/) and IntAct (https://www.ebi.ac.uk/intact/home). Protein sequences (FASTA files) were obtained from UniProt (https://www.uniprot.org/). Protein pathway and functional mapping information was derived from Reactome Pathway (https://reactome.org/) and GenoOnthology (http://geneontology.org/) databases.

Human research participants

Policy information about studies involving human research participants and Sex and Gender in Research.

Reporting on sex and gender	Reporting procedure was not part of this study. The source of samples consists of de-identified unused portions of surgical specimens that were taken for reasons other than research (i.e. for breast cancer patients undergoing the procedures for medical reasons unrelated to need for research samples or to the nature of the research). No individuals were excluded on the basis of age, sex, gender or ethnicity. Because breast cancer is a disease which overwhelmingly affects women, and is a disease that is generally not seen in children, the vast majority of patients enrolled were females >18yrs of age. The patient sex and gender was determined based on self-reporting. An ethnically and socioeconomically diverse population is likely represented in the study as anticipated based on the New York demographics. Informed consent and HIPAA compliance forms were obtained from all subjects prior to their enrollment. Samples were de-identified before receipt for use in the studies.
Population characteristics	see above. Because breast cancer is a disease which overwhelmingly affects women, and is a disease that is generally not seen in children, the vast majority of patients enrolled were females >18yrs of age.
Recruitment	Recruitment procedure was not part of this study (see also above).
Ethics oversight	Surgical specimens were obtained in accordance with the guidelines and approval of the Institutional Review Board at Memorial Sloan Kettering Cancer Center, Biospecimen Research Protocol# 09-121, project title: Ex-Vivo Testing of Breast Cancer Tumors for Sensitivity to Inhibitors of Heat Shock Proteins and Signaling Pathway Inhibitors, S. Modi, PI. Patient tissue samples were obtained with informed consent. Samples were de-identified before receipt for use in the studies.

Note that full information on the approval of the study protocol must also be provided in the manuscript.

Field-specific reporting

Please select the one below that is the best fit for your research. If you are not sure, read the appropriate sections before making your selection.

☒ Life sciences ☐ Behavioural & social sciences ☐ Ecological, evolutionary & environmental sciences

For a reference copy of the document with all sections, see nature.com/documents/nr-reporting-summary-flat.pdf

Life sciences study design

All studies must disclose on these points even when the disclosure is negative.

Sample size	No statistical methods were used to predetermine sample size for experiments but these are similar to those generally employed in the field and reported to result in significant observable differences (refs. 17-19). Sample sizes are fully disclosed in the manuscript.
Data exclusions	No data were excluded from the analyses.
Replication	All in vitro and in vivo experiments were performed, in at least 3 biological replicates, with biological and technical replicates which are fully disclosed in the manuscript. Several alternative methods were used to validate observations. Experiments were also replicated through multiple cohort analyses. Results shown are representative of several independently performed experiments (see figure legends, at least 3). There were no findings that could not be replicated or reproduced.
Randomization	For all experiments, samples were allocated into experimental groups by randomization.
Blinding	Investigators were not blinded to group allocation during data collection and/or analysis for the preclinical studies. Blinding was not relevant to the experiments described in the study due to primary investigators performing experiments from start to finish due to technicality required. Blinding was only applied for the assessment of explants: Apoptosis and necrosis of the tumor cells (as percentage) was evaluated by a pathologist by reviewing all the H&E slides of the case (controls and treated ones) in toto, blindly, allowing for better estimation of the overall treatment effect to the tumor.

Reporting for specific materials, systems and methods

We require information from authors about some types of materials, experimental systems and methods used in many studies. Here, indicate whether each material, system or method listed is relevant to your study. If you are not sure if a list item applies to your research, read the appropriate section before selecting a response.

Materials & experimental systems

n/a	Involved in the study	
☐	☒	Antibodies
☐	☒	Eukaryotic cell lines
☒	☐	Palaeontology and archaeology
☒	☐	Animals and other organisms
☒	☐	Clinical data
☒	☐	Dual use research of concern

Methods

n/a	Involved in the study	
☒	☐	ChIP-seq
☐	☒	Flow cytometry
☒	☐	MRI-based neuroimaging

Antibodies

Antibodies used	All antibodies and relevant information is provided in the Methods and are also listed below: β-actin (A1978, Sigma-Aldrich, RRID: AB_476692, 1:3000) or GAPDH (2118, Cell Signaling, RRID: AB_561053, 1:10000) were used as protein loading controls. Primary antibodies used in this study are listed below. HSP70 (ADI-SPA-810, RRID:AB_10616513, 1:2000), HSC70 (ADI-SPA-815, RRID:AB_10617277, 1:3000), HOP (SRA-1500, RRID:AB_10618972, 1:2000), HSP60 (ADI-SPA-806, RRID:AB_10617232, 1:1000) and HSP40 (SPA-400, RRID:AB_11180881, 1:3000) were purchased from Enzo; HSP90β (SMC-107, RRID:AB_854214, 1:4000) and HSP110 (SPC-195, RRID:AB_2119373, 1:2000) antibodies were from Stressmarq; HSP90α (ab2928, RRID:AB_303423, 1:5000), AHA1 (ab56721, RRID:AB_2273725, 1:1000), FAM29A/HAUS6 (ab103979, RRID:AB_10712429, 1:1000), caspase 10 (ab177475, RRID: AB_2924729, 1:1000), Securin (ab3305, RRID:AB_2173413, 1:1000), Pericentrin (ab4448, RRID:AB_304461, 1:100) and NEDD1 (ab57336, RRID:AB_944385, 1:100), from Abcam; cleaved PARP (G734A, RRID:AB_430876, 1:1000) from Promega; CDC37 (4793, RRID:AB_10695539, 1:3000), phospho-AKT (S473) (9271, RRID:AB_329825, 1:2000), AKT (4691, RRID:AB_915783, 1:3000), phospho-ERK (T202/Y204) (4377, RRID:AB_331775, 1:3000), ERK (4695, RRID:AB_390779, 1:5000), MCL1 (5453, RRID:AB_10694494, 1:1000), RAF1 (12552, RRID:AB_2728706, 1:1000), BCL6 (14895, RRID:AB_2798638, 1:1000), eIF4E (2067, RRID:AB_2097675, 1:2000), phospho-eIF4E (S209) (9741, RRID:AB_331677, 1:500), Eg5 (14404, RRID:AB_2798473, 1:1000), phospho-STAT3 (Y705) (9145, RRID:AB_2491009, 1:1000), STAT3 (9139, RRID:AB_331757, 1:2000), phospho-Aurora A (3079, RRID:AB_2061481, 1:1000), Aurora A (91590, RRID:AB_2800171, 1:1000), PLK1 (4513, RRID:AB_2167409, 1:2000), TPX2 (12245, RRID:AB_2716832, 1:250), EGFR (4267, RRID:AB_2246311, 1:1000), Cyclin A2 (67955, AB_2909603, 1:1000), NuMA (ABE1361, RRID:AB_2892052, 1:1000), p-MPM2 (Millipore, #05-368, RRID:AB_309698, 1:500) from EMD Millipore; NuMA (NB100-74636, RRID:AB_1049265, 1:1000) from Novus Biologicals; α-tubulin (T5168, RRID:AB_477579, 1:5000) from Sigma-Aldrich; Cyclin B1 (554178, RRID:AB_395289, 1:1000), Nek2 (610593, RRID:AB_397933, 1:1000) and Aurora-A (610938, RRID:AB_398251, 1:1000) were from BD Transduction Laboratories; Cyclin A (sc H-432, RRID:AB_631329, 1:1000), Cdc20 (sc-8358, RRID:AB_2291311, 1:1000) and normal rat IgG (sc-2026, Santa Cruz, RRID:AB_737202) were from Santa Cruz. Cy5-conjugated anti-mouse secondary antibody (A10524, RRID:AB_2534033, 1:2,000), Alexa Fluor 488 conjugated (A-11004, RRID:AB_2534072, 1:2000) and Alexa Fluor 568 conjugated (A-11008 (RRID:AB_143165),1:2000) were from Life technologies. Native PAGE: HSP90β (SMC-107, RRID:AB_854214, 1:3,000) and HSP110 (SPC-195, RRID:AB_2119373, 1:2,000) antibodies were purchased from Stressmarq; HSP70 (SPA-810, RRID:AB_10616513, 1:2,000), HSC70 (SPA-815, RRID:AB_10617277, 1:3,000), HOP (SRA-1500, RRID:AB_10618972, 1:2,000) from Enzo; HSP90α (ab2928, RRID:AB_303423, 1:6,000) from Abcam. Horseradish peroxidase (HRP)-conjugated secondary antibody were purchased from SouthernBiotech - goat anti-rat

	IgG-HRP (3030-05, Lot# J1713-M322, 1:5000), goat anti-rabbit Ig, human ads-HRP (4010-05, Lot# A4211-ZH10E, 1:5000) and goat anti-mouse IgG, human ads-HRP (1030-05, Lot# D1922-X922, 1:5000). For immunoprecipitation: HSC70 antibody (ADI-SPA-815, Enzo, RRID:AB_10617277, 1:100) or normal rat IgG (sc-2026, Santa Cruz, RRID:AB_737202, 1:100).
Validation	All antibodies are commercially available and have been validated by the manufacturer. Supporting publications are found on the manufacturer's site (https://www.sigmaaldrich.com/US/en/products/protein-biology/antibodies; https://www.enzolifesciences.com/browse/products/by-product-type/antibodies/; https://www.stressmarq.com/product-category/antibodies/?v=7516fd43adaa; https://www.abcam.com/nav/primary-antibodies; https://www.promega.com/products/protein-detection/primary-and-secondary-antibodies/; https://www.emdmillipore.com/US/en/life-science-research/antibodies-assays/6tWb.qB.8EAAAAE_e5V3.M6B,nav; https://www.novusbio.com/; https://www.biocompare.com/Antibodies/; https://www.scbt.com/browse/Primary-Antibodies-and-ImmunoCruz-sup-reg-sup-Conjugates/_/N-gz9ft1; https://www.cellsignal.com/; https://www.thermofisher.com/us/en/home/brands/life-technologies.html). Manufacturer citations are listed in manufacturer website for each specific antibody. We have used recommended antibody dilutions for western blot experiments, and detected bands of expected molecular weight, as described by the manufacturers. Relevant positive (homogenates of cells containing the protein to be detected) and negative (homogenates of cells lacking the protein to be detected) controls were used to further validate several antibodies as indicated in the relevant figures.

Eukaryotic cell lines

Policy information about cell lines and Sex and Gender in Research

Cell line source(s)	Cell lines selection was not based on gender, sex or ethnicity. Cell lines were cultured according to the providers' recommended culture conditions. Cells were authenticated using short tandem repeat profiling and tested for mycoplasma. Cell lines selection was not based on gender, sex or ethnicity. Cell lines were cultured according to the providers' recommended culture conditions. Cells were authenticated using short tandem repeat profiling and tested for mycoplasma. Breast cancer cell lines MDA-MB-468 (HTB-132, RRID: CVCL_0419), MCF-7 (HTB-22, RRID: CVCL_0031), SK-Br-3 (HTB-30, RRID: CVCL_0033), AU565 (CRL-2351, RRID: CVCL_1074), T-47D (HTB-133, RRID: CVCL_0553), BT-20 (HTB-19, RRID: CVCL_0178), MDA-MB-415 (HTB-128, RRID: CVCL_0621), MDA-MB-453 (HTB-131, RRID: CVCL_0418), HCC1806 (CRL-2335, RRID: CVCL_1258), MDA-MB-361 (HTB-27, RRID: CVCL_0620), MDA-MB-231 (CRM- HTB-26, RRID: CVCL_0062) were purchased from ATCC. Leukemia cell lines KASUMI-1 (CRL-2724, RRID: CVCL_0589) and K562 (CCL-243, RRID: CVCL_0004) were obtained from ATCC and MOLM-13 (ACC-554, RRID: CVCL_2119) was from DSMZ. Pancreatic cancer cell lines ASPC-1 (CRL-1682, RRID: CVCL_0152), BxPc-3 (CRL-1687, RRID: CVCL_0186), SU.86.86 (CRL-1837, RRID: CVCL_3881), Capan-1 (HTB-79, RRID: CVCL_0237), Capan-2 (HTB-80, RRID: CVCL_0026), CFPAC (CRL-1918, RRID: CVCL_1119), Panc-1 (CRL-1469, RRID: CVCL_0480) and MiaPaCa2 (CRL-1420, RRID: CVCL_0428) were from ATCC. 931102 and 931019 were patient derived cell lines provided by Dr. Y. Janjigian, MSKCC21. Gastric cancer cell line MKN74 (RRID:CVCL_2791) was obtained from G. Schwarz (Columbia University) and obtained from AcceGen Biotechnology and OE19 (ACC-700, RRID: CVCL_1622) from DSMZ. The ovarian cancer cell lines PEO-1 (RRID:CVCL_2686), PEO-4 (RRID:CVCL_2690), OVCAR4 (RRID:CVCL_1627), OV1847 (RRID: CVCL_D703), A2780 (RRID:CVCL_0134), IGROV-1 (RRID:CVCL_1304) and OVCAR5 (RRID:CVCL_1628) were kindly provided by Dr. D. Solit, MSKCC and obtained from Millipore Sigma. Lung cancer cell line NCI-H3122 (RRID:CVCL_5160) was provided by M. Moore, MSKCC and obtained from Creative Biolabs, and NCI-H2228 (CRL-5935, RRID: CVCL_1543), NCI-H1975 (CRL-5908, RRID: CVCL_1511), NCI-H1373 (CRL-5866, RRID: CVCL_1465), A549 (CCL-185, RRID: CVCL_0023), NCI-H647 (CRL-5834, RRID: CVCL_1574), NCI-H526 (CRL-5811, RRID: CVCL_1569) were obtained from ATCC. Epstein-Barr virus positive Burkitt's lymphoma cell lines Akata1 (RRID:CVCL_0148)80, Mutu1 (RRID:CVCL_7202)80 and Rael (RRID:CVCL_7208)80 were provided by W. Tam (WCMC, obtained from Lonza) and BCP-1 (CRL-2294, RRID: CVCL_0107), Daudi (CCL-213, RRID: CVCL_0008), EB1 (HTB-60, RRID: CVCL_2027), NAMALWA (CRL-1432, RRID: CVCL_0067), P3HR-1 (HTB-62, RRID: CVCL_2676), SU-DHL-6 (CRL-2959, RRID: CVCL_2206), Farage (CRL-2630, RRID: CVCL_3302) and Pfeiffer (CRL-2632, RRID: CVCL_3326) were obtained from ATCC; HBL-1 (RRID:CVCL_4213) from Applied Biological Materials (abm), MD901 (RRID:CVCL_D709) [PubMed=8274736] and U2932 (RRID:CVCL_1896) from DSMZ were kindly provided by J. Angel Martinez-Climent (Centre for Applied Medical Research, Pamplona, Spain); Karpas422 (ACC-32, RRID: CVCL_1325), RCK8 (ACC-561, RRID: CVCL_1883) and SU-DHL-4 (ACC-495, RRID: CVCL_0539) were obtained from the DSMZ; OCI-LY1 (RRID:CVCL_1879), OCI-LY4 (RRID:CVCL_8801), OCI-LY7 (RRID:CVCL_1881) were obtained from the Ontario Cancer Institute; TMD8 (RRID:CVCL_A442) [PMID: 16780947] was kindly provided by L. M. Staudt (NIH); BC-1 (RRID:CVCL_1079) was derived from an AIDS-related primary effusion lymphoma[PubMed=30894373]; IBL-1 (RRID:CVCL_9638) was derived from an AIDS-related immunoblastic lymphoma[PubMed=18230756] and BC-3 (RRID:CVCL_2099) was derived from a non-HIV primary effusion lymphoma[PMID: 23943653]. Neuroblastoma cells SY5Y (CRL-2266, RRID: CVCL_0019) was purchased from ATCC; LAN5 (RRID:CVCL_0389) and SMS-KCNR (RRID:CVCL_7134) were obtained from the Children's Oncology Group (COG). Multiple Myeloma cell lines U266 (RRID:CVCL_0566) and MM.1R (RRID:CVCL_8794) from ATCC were kindly provided from Dr. Z. Li (OSU), PCNY1 was derived as reported81, and NCI-H929 (CRL-9068, RRID: CVCL_1600) was purchased from ATCC. Ewing's sarcoma cells TC71 (RRID:CVCL_2213) from DSMZ and A673 (RRID:CVCL_0080) from ATCC were kindly provided by Dr. S. Ambati, MSKCC. Cervical cancer cells HeLa (CCL-2, RRID: CVCL_0030) and colon fibroblasts CCD-18 (CRL-1459, RRID: CVCL_2379) were purchased from ATCC. HeLa cells expressing histone 2B-mCherry and α-tubulin EGFP were kindly provided by Dr. Daniel Gerlich, Institute of Molecular Biotechnology, Austria and were previously reported82
Authentication	Cell were authenticated using short tandem repeat profiling
Mycoplasma contamination	Cells were routinely tested for mycoplasma and were found to be negative
Commonly misidentified lines<br>(See ICLAC register)	None was used

Flow Cytometry

Plots

Confirm that:

☒ The axis labels state the marker and fluorochrome used (e.g. CD4-FITC).

☒ The axis scales are clearly visible. Include numbers along axes only for bottom left plot of group (a 'group' is an analysis of identical markers).

☒ All plots are contour plots with outliers or pseudocolor plots.

☒ A numerical value for number of cells or percentage (with statistics) is provided.

Methodology

Sample preparation	For the cell cycle experiments, cells dissociation was done by trypsinization. Cells were washed with PBS, followed by overnight fixation with 70% ethanol. For cell death analysis, cells were collected by pipetting up and down gently in 1 ml of media.
Instrument	LSRfortessa (BD)
Software	FlowJo version 10
Cell population abundance	After excluding debris and doublets, 100% of cells were relevant for the study, as we used cell culture condition (100% purity of cells of interest)
Gating strategy	The cells were first gated based on their unique forward (FSC) and side scatters (SSC) properties to exclude the cell debris. Subsequently, cells were gated using FSC-H vs. FSC-A and SSC- H vs. SSC-A plots to exclude the doublets and obtain single cells, which were then used for cell cycle and cell death analysis.

☒ Tick this box to confirm that a figure exemplifying the gating strategy is provided in the Supplementary Information.
